# Latent Membrane Protein 1 (LMP1) from Epstein–Barr Virus (EBV) Strains M81 and B95.8 Modulate miRNA Expression When Expressed in Immortalized Human Nasopharyngeal Cells

**DOI:** 10.3390/genes13020353

**Published:** 2022-02-16

**Authors:** Barbara G. Müller Coan, Ethel Cesarman, Marcio Luis Acencio, Deilson Elgui de Oliveira

**Affiliations:** 1 Biosciences Institute of Botucatu, São Paulo State University (UNESP), Botucatu 18618-689, SP, Brazil; barbaramcoan@gmail.com; 2 Department of Pathology and Laboratory Medicine, Weill Cornell Medicine, New York, NY 10065, USA; ecesarm@med.cornell.edu; 3 Luxembourg Centre for Systems Biomedicine (LCSB), University of Luxembourg, Belvaux, L-4367 Luxembourg, Luxembourg; marcio.acencio@uni.lu; 4 Department of Pathology, Medical School, São Paulo State University (UNESP), Botucatu, SP, 18618-687, Brazil; 5 ViriCan, Institute for Biotechnology (IBTEC), São Paulo State University (UNESP), Botucatu, SP, 18607-440, Brazil

**Keywords:** EBV, LMP1, microRNAs, nasopharyngeal cells, expression profiling

## Abstract

The Epstein–Barr virus (EBV) is a ubiquitous γ herpesvirus strongly associated with nasopharyngeal carcinomas, and the viral oncogenicity in part relies on cellular effects of the viral latent membrane protein 1 (LMP1). It was previously described that EBV strains B95.8 and M81 differ in cell tropism and the activation of the lytic cycle. Nonetheless, it is unknown whether LMP1 from these strains have different effects when expressed in nasopharyngeal cells. Thus, herein we evaluated the effects of EBV LMP1 derived from viral strains B95.8 and M81 and expressed in immortalized nasopharyngeal cells NP69^SV40T^ in the regulation of 91 selected cellular miRNAs. We found that cells expressing either LMP1 behave similarly in terms of NF-kB activation and cell migration. Nonetheless, the miRs 100-5p, 192-5p, and 574-3p were expressed at higher levels in cells expressing LMP1 B95.8 compared to M81. Additionally, results generated by in silico pathway enrichment analysis indicated that LMP1 M81 distinctly regulate genes involved in cell cycle (i.e., *RB1*), mRNA processing (i.e., *NUP50*), and mitochondrial biogenesis (i.e., *ATF2*). In conclusion, LMP1 M81 was found to distinctively regulate miRs 100-5p, 192-5p, and 574-3p, and the in silico analysis provided valuable clues to dissect the molecular effects of EBV LMP1 expressed in nasopharyngeal cells.

## 1. Introduction

Cancers are an important cause of human mortality and lethality in adults world-wide, being the second leading cause of global deaths in 2013 [1]. In 2008, 16% of all cancer cases were related to infection, and over 67% of these were viral agents [2,3,4]. Epstein–Barr virus (EBV) is a human γ herpesvirus associated with many cancers, notably Burkitt lymphoma and undifferentiated nasopharyngeal carcinoma (NPC) [2,5,6]. EBV is ubiquitous and causes lifelong latent infection in over 90% of adults worldwide [2]. The primary infection is usually asymptomatic but can be associated with clinical signs of infectious mononucleosis, mostly in cases of late exposure to EBV [7].

NPC is strongly associated with EBV infection, notably the undifferentiated form—in which EBV is detected within the neoplastic cells in virtually all cases. NPC is an aggressive epithelial cancer, prone to invade adjacent tissue and lymph nodes [8]. The disease has a poor prognosis, and it was responsible for over 86,000 new cases and 50,000 deaths worldwide in 2012, being more prevalent in men [2,9]. The incidence of NPC changes according to geographic localization, and the disease is endemic in southeast Asia, southwest China, and Micronesia. Besides EBV infection, risk factors for NPC include other environmental exposures (e.g., nitrosamides, tobacco, insufficient ventilation of dwellings) and genetic factors, such as polymorphisms in genes encoding leukocyte antigen (HLA) class I and class II molecules, the heat shock 70 kDa protein (HSP 70) or the polymeric immunoglobulin (Ig) A receptor. Furthermore, the NPC incidence may also be influenced by different EBV strains [10,11,12].

The progression of EBV-associated cancers can be affected by the activity of viral gene products [13,14]. In this regard, the Latent Membrane Protein 1 (LMP1)—one of the major EBV oncoproteins—induces a variety of changes in cell behavior, including proliferative and survival capabilities [15], altered cell adhesion, extracellular matrix (ECM) and vascular remodeling, and an increase in cell motility. Ultimately, these processes may lead to a more aggressive and metastatic carcinoma [16,17,18].

Different EBV viral strains can distinctly influence the behavior of infected cells. For instance, EBV strains GP202 (isolated from gastric cancer), B95-8, and AKATA (both from lymphomas) were reported to induce cell growth more efficiently than M81 (obtained from NPC), YCCEL1, or SNU719 (both from gastric cancer) [19]. M81 was also reported to behave differently from the viral prototype strain B95-8 (isolated from Burkitt lymphoma), SNU719, GP202, or YCCEL1, showing a higher affinity for epithelial cell infection (consistent with its epithelial cancer origin) and higher capability of lytic cycle induction [19,20]. Notably, chromosomal instability can be achieved by a high expression of the lytic gene BZLF1 and recurring induction of the lytic cycle, leading to increased transformation properties and higher risk for NPC [19].

LMP1 may affect endogenous microRNA (miRNA) expression in both B cells and epithelial cells, causing effects relevant to cancer progression [21,22,23,24]. Essentially all phenomena related to cancer progression can be regulated by microRNAs [25,26,27,28,29], and different EBV strains may show unique biological properties. Thus, it is plausible to assume that distinct EBV strains may regulate a unique set of human miRNAs, with possible consequences for EBV-induced carcinogenesis. Based on these premises, in this study, we investigated whether nasopharyngeal cells expressing LMP1 from M81 or B95.8 strains differ in miRNAs expression and examined the possible consequences regarding cell signaling pathways by in silico analysis.

## 2. Materials and Methods

### 2.1. Cell Culture

The cell lines HEK293 (RRID:CVCL_0045) and NP69^SV40T^ (hereafter referred to as NP69; RRID:CVCL_F755;) were used in this study. HEK293 is an immortalized human embryonic kidney cell line harboring DNA fragments from type 5 adenovirus [30]. NP69 cells were generated by immortalizing primary nasopharyngeal epithelial cells with the SV40 large T antigen, and they retain many characteristics of normal nasopharyngeal cells (e.g., the profile of keratin expression and responsiveness to TGFβ inhibition). Despite some genetic alterations also found in nasopharyngeal carcinomas, NP69 is non-tumorigenic and highly responsive to the EBV LMP1 expression [31]. Based on that, this cell line was used to assess the expression of human microRNAs and behavioral changes induced by expression of EBV LMP1 derived from strain B95.8 and M81.

Both cell lines had their genetic identity assured by short tandem repeats (STRs) profiling using the GenePrint 10 system (Promega, Madison, WI, USA), and they were verified to be free of mycoplasma contamination by a PCR-based assay. HEK293 cells were maintained in Dulbecco’s Modified Eagle Medium (DMEM), supplemented with 10% Fetal Bovine Serum (FBS) and 0.4% gentamicin, while NP69 cells were cultivated with Keratinocyte Serum-Free medium (K-SFM-Life Technologies, Carlsbad, CA, USA) supplemented with 5% FBS, 25 µg/mL of Bovine Pituitary Extract (BPE), 0.2 ng/mL of Epidermal Growth Factor (EGF), and 1% Gentamicin. Both were cultivated at 37 °C in a humid atmosphere with 5% CO_2_.

### 2.2. LMP1 Constructs and Cell Transfections

The open reading frames (ORF) for LMP1 derived from EBV strains B95.8 and M81 were retrieved from recombinant virus constructs kindly provided by Prof. Henri-Jacques Delecluse (German Cancer Research Center, Heidelberg, Germany). Recombinant DNA plasmids were assembled with the F factor-based prokaryotic replicon, pMBO131, with flanking regions for homologous recombination of the viral genome [32,33]. The obtained ORFs were transferred to the pEF1α-IRES-ZsGreen1 backbone vector (Clontech, Mountain View, CA, USA), allowing the detection of LMP1-expressing cells due to the simultaneous expression of a green fluorescent protein (GFP) reporter. The EBV LMP1 ORFs were amplified from the original vectors using primers harboring restriction sites for the enzymes EcoRI and BamHI, used for unidirectional cloning (see Additional File 1: Table S1).

The new LMP1-encoding constructs (Supplementary Figure S1), dubbed pZsG-LMP1-B95.8 and pZsG-LMP1-M81, were validated by Sanger DNA sequencing and the sequences were deposited in GenBank (Accession codes #MN062162 and #MN062163). For transient cellular transfections, 0.7 × 10^5^ cells were seeded in 24-well plates 24 h prior to the assay, carried out with the pZsGreen backbone, pZsG-LMP1B95.8 or pZsG-LMP1M81 constructs, and Lipofectamine 3000 (Thermo Fisher Scientific, Waltham, MA, USA) as the transfection reagent, following the manufacturer’s recommendations. Subsequent assays were performed 48 h post-transfection (Supplementary Figure S2).

### 2.3. Luciferase Assay and Cell Migration In Vitro

Both assays were performed with 0.7 × 10^5^ cells (HEK293 or NP69) seeded into 24-well plates 24 h prior to transfection, and the cells were used for experiments 48 h post-transfection. The luciferase reporter assay was performed with HEK293 cells (given its high susceptibility to liposomal transfection) to validate the EBV LMP1 activity using its effect on NF-κB activation as a proxy. Briefly, HEK293 cells were co-transfected with the pEF1α-IRES-ZsGreen1 backbone vector or the LMP1 B95.8 and M81 constructs, along with pcDNA3.1-NF-κBLuc, for the expression of firefly luciferase under the control of a NF-κB-responsive element) [34], and Promega pGL4.74 (for constitutive expression of *Renilla* luciferase, to normalize results for the transfection efficiency levels). Results were generated in 96-well white plates using the Dual-Luciferase^®^ Reporter Assay System and the GloMax Explorer device (both manufactured by Promega).

The rates of cell migration in vitro were assessed with the scratch wound healing assay. Images were taken at the time of the scratch and 24 h later (48 and 72 h post-transfection) were evaluated using the TScratch software [35] to estimate the percentage of closure of the wounded area in the cell monolayer.

For all assays, the results were obtained from three independent experiments performed with triplicates. The analysis of data was performed with Student’s *t*-test, taking a *p*-value ≤ 0.05 as statistically significant.

### 2.4. MicroRNA Expression Analysis

The analysis of miRNA expression was performed with a custom panel of 91 human miRNAs (see Supplementary Table S2), selected based on literature data showing changes in expression observed in NPC or other human cancers, associated or not with EBV. These experiments were performed with NP69 cells transfected with the pEF1α-IRES-ZsGreen1 backbone vector and the constructs pZsG-LMP1-B95.8 and pZsG-LMP1-M81. After transfection, the cells were subjected to FACS (Supplementary Figure S3 and Supplementary Table S3) to recover GFP-positive cells, aiming to enrich cells successfully transfected with the vectors for LMP1 expression. Briefly, the transfected cells were cultivated for 48 h post-transfection, then detached and flowed through a 70 μm filter. GFP-positive cells were sorted using a BD FACS Aria III (BD Bioscience, San Jose, CA, USA), pelleted by centrifugation, and cryopreserved in liquid nitrogen. Afterward, the enriched NP69 transfected cells were subjected to miRNAs-enriched RNA extraction with the miRNeasy^®^ Mini Kit, reverse-transcribed using miScript II RT Kit, then validated with the miScript^®^ QC PCR Array system (all Qiagen products), following the manufacturer’s instructions. Upon successful quality validation of samples, the qPCR assays were performed using a customized miScript miRNA PCR Array platform (Qiagen, Valencia, CA, USA) with the threshold and baselines determined by the manufacturer’s recommendations. Statistics analyses were performed using the online software provided by the GeneGlobe Data Analysis Center (Qiagen’s mirScript miRNA PCR Arrays & Assays), using the recommended parameters and three endogenous controls. The experiments were performed with biological triplicates (Supplementary Tables S4 and S5).

### 2.5. MiRNA’s Targets Prediction and Pathway Enrichment Analysis

The miRNAs showing differential expression (*p*-value ≤ 0.05) with a fold change of ± 1.2 were selected based on the following comparation sets: (1) EBV LMP1 B95.8 vs. control (backbone vector); (2) EBV LMP1 M81 vs. control; and (3) EBV LMP1 M81 vs. EBV LMP1 B95.8. The selection of cut-off points was performed as reported previously [36,37]. The differentially expressed miRNAs from sets 1, 2, and 3 were subjected to target prediction analysis using the mirDIP online tool [38,39], and the top 1% target genes were subjected to the pathway-enrichment analysis using the ReactomeFIViz [40] plugin in the Cytoscape software [41]. Further details are available in the Supplementary Material.

## 3. Results

### 3.1. Cells Expressing EBV LMP1 from Viral Strains B95.8 and M81 Behaves Similarly in Terms of NF-κB Activation and Cell Migration Rates In Vitro

**Figure 1:**
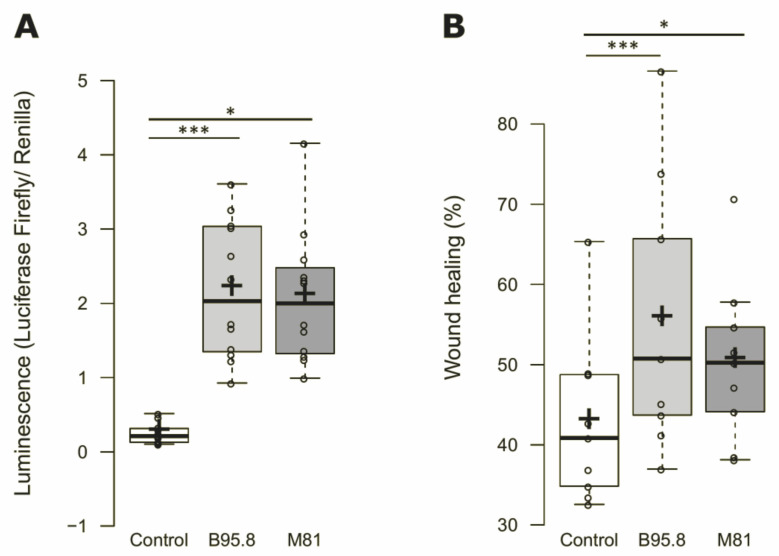
Effects of expression of EBV LMP1 variants M81 or B95.8 in transfected cells in vitro: Luciferase and migration assays with HEK293 (**A**) and NP69SV40T (**B**) cells transfected with pZsGreen (control), pZsG-LMP1-B95.8 or pZsG-LMP1-M81 vectors. (**A**) NF-κB activity assessed via Luciferase reporter assay showing a 9-fold increase in LMP1 expressing HEK293 cells. (**B**) In vitro migration assay showing an increase in migration of 26% and 15% of NP69SV40T cells transfected with LMP1 B95.8 or M81 respectively. The mean values for standard error obtained from at least three independent experiments are shown. *p* values < 0.05 and < 0.005 are indicated by * and ***, respectively.

EBV LMP1 is known to activate NF-κB, and this property can be used as a proxy to validate the functional integrity of LMP1 expressed ectopically. To verify whether the constructs generated to allow the expression of functional LMP1, the activation of NF-kB was measured in HEK293 cells using a luciferase reporter assay with luciferase being expressed in an NF-kB-dependent manner. HEK293 cells expressing EBV LMP1 showed a ninefold increase in NF-κB activation compared to the cells transfected with the empty, backbone vector, and a significant difference was observed considering the activation levels obtained by LMP1 from strains B95.8 and M81 (Figure 1A). Thus, the viral oncoprotein expressed by both LMP1 constructs is functional, and the LMP1 encoded by either of the viral strains activates NF-κB at similar levels.

Furthermore, transiently transfected NP69 cells expressing LMP1, as confirmed by RT-qPCR, showed higher migration rates compared to the control (15% and 26% for M81 or B95.8 variants, respectively, (see Figure 1B)). Additionally, both EBV LMP1-encoding constructs could induce increased cell migration in vitro, but no significant differences were found comparing the LMP1 derived from EBV strains M81 and B95.8.

### 3.2. EBV LMP1 Modulate miRNA Expression in NP69^SV40T^ Cells

Although it is known that the EBV strains B95.8 and M81 have distinct biological features [20], it remains to be better understood whether their differences also impact the oncogenic properties of the virus. We aimed to investigate this by interrogating whether LMP1 from different viral strains has different effects on miRNA expression, which may also give relevant clues on broad cellular effects of this major EBV oncoprotein. Changes in the levels of selected miRNAs were evaluated in NP69 cells expressing LMP1 derived from viral strains B95.8 or M81, enrichment for GFP-positive cells by FACS (Supplementary Figure S3 and Table S3). The customized qPCR array used allowed the evaluation of 91 selected human miRNAs (Supplementary Table S2), and the raw results were subjected to data normalization prior to the downstream analysis (Supplementary Tables S4 and S5, respectively).

**Figure 2:**
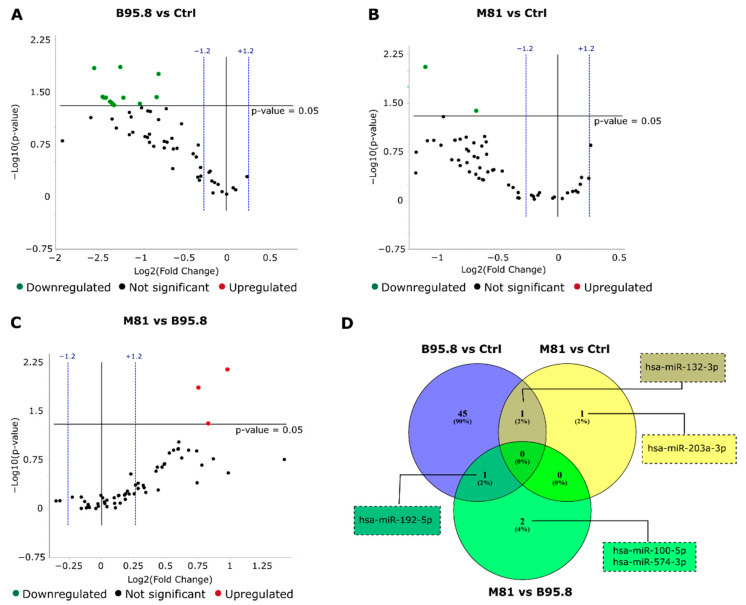
Effects of expression of EBV LMP1 derived from viral strains B95.8 and M81 on miRNA expression in immortalized nasopharyngeal cells NP69^SV40T^. miRNA RT-qPCR array with 91 selected miRNAs was performed on cells transfected with pZsGreen (control), pZsG-LMP1-B95.8 or pZsG-LMP1-M81 vectors. All results were obtained from three independent experiments. (**A**) A total of 47 miRNAs were downregulated in group LMP1 B95.8 vs. control vector (B95.8 vs. Ctrl), with fold regulation between −1.7 and −2.9. (**B**) 2 miRNAs were downregulated in group LMP1 M81 vs. control (M81 vs. Ctrl) with fold regulation of −1.6 and −2.1. (**C**) A total of 3 upregulated miRNAs were seen in group LMP1 M81 vs. LMP1 B95.8 (M81 vs. B95.8) with fold regulation between 1.7 and 2. (**D**) miRNAs exclusive or commonly altered between groups. MiR-132-3p was found for both B95.8 vs. Ctr or M81 vs. Ctrl and miR-192-5p was altered in groups B95.8 vs. Ctrl and M81 vs. B95.8. Selected miRNAs had *p*-value ≤ 0.05 and fold regulation of ± 1.2.

Three comparisons were performed: (1) EBV LMP1-B95.8 compared to control (dubbed B95.8 vs. Ctrl); (2) EBV LMP1-M81 compared to control (M81 vs. Ctrl); and (3) EBV LMP1 M81 compared to EBV LMP1 B95.8 (M81 vs. B95.8). We observed the downregulation of 47 miRNAs in B95.8 vs. Ctrl (fold regulation: −1.7 to −2.9; Figure 2A), 2 miRNAs in M81 vs. Ctrl (fold regulation: −1.6 to −2.1; Figure 2B), and upregulation of 3 miRNAs comparing M81 vs. B95.8 (fold regulation varying from 1.7 to 2; Figure 2C). The miR-132-3p was found downregulated in both B95.8 vs. Ctrl and M81 vs. Ctrl comparisons (fold regulation: −2.4 and −2.1, respectively). It is worth noting that the miRNA upregulation in M81 vs. B95.8 indicates that, compared to the control, the EBV LMP1 from B95.8 strain induced a more robust downregulation of miRNAs compared to LMP1 derived from strain M81.

**Figure 3:**
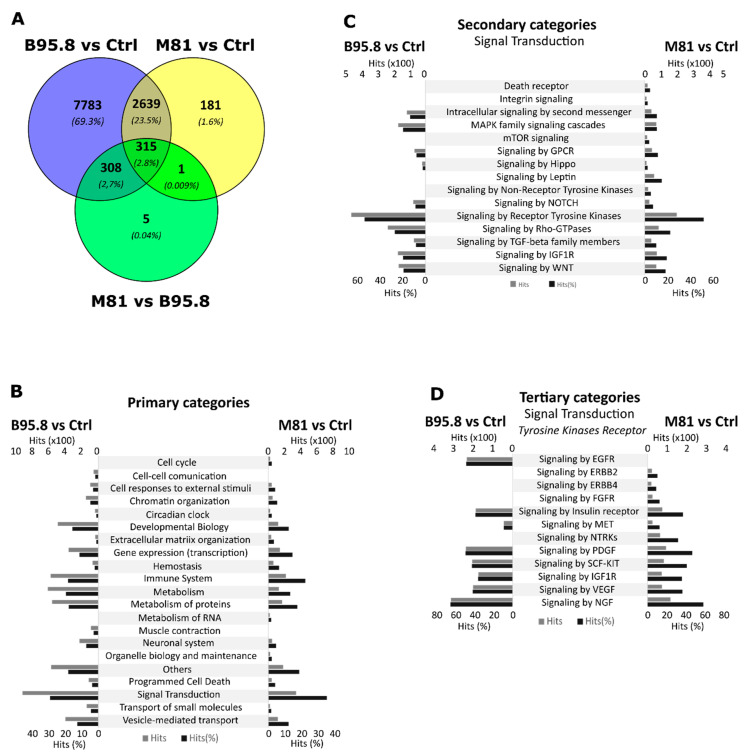
Analysis of target genes from differentially expressed miRNAs in NP69SV40T cells transfected with EBV LMP1 from B95.8 or M81 variants. (**A**) Number and percentage of unique or commonly target genes considering three comparison groups: EBV LMP1 B95.8 vs. Ctrl, EBV LMP1 M81 vs. Ctrl, LMP1 M81 vs. viral LMP1 B95.8. (**B**) Primary categories, (**C**) Secondary categories of “Signal Transduction” and (**D**) Tertiary categories of “Tyrosine kinase receptor signaling” altered by predicted gene targets of miRNA regulated by LMP1 variants B95.8 (**left**) or M81 (**right**). In (**B**–**D**) the light and dark grey indicate, respectively, the absolute number of hits or their percentage considering the respective higher category.

Next, we sought to perform pathway enrichment analysis in silico to extrapolate biological significance for the obtained miRNA expression profiles. For each given comparison (B95.8 vs. Ctrl, M81 vs. Ctrl, and M81 vs. B95.8), we investigated the number of predicted target genes of all differentially expressed miRNAs. We observed 11,045, 3136, and 629 predicted genes for B95.8 vs. Ctrl, M81 vs. Ctrl, and M81 vs. B95.8, respectively. Moreover, 315 (2.8%) targets were shared between all three comparison sets, whereas 2639 (23.5%) were shared only between B95.8 vs. Ctrl and M81 vs. Ctrl (Figure 3A).

**Figure 4:**
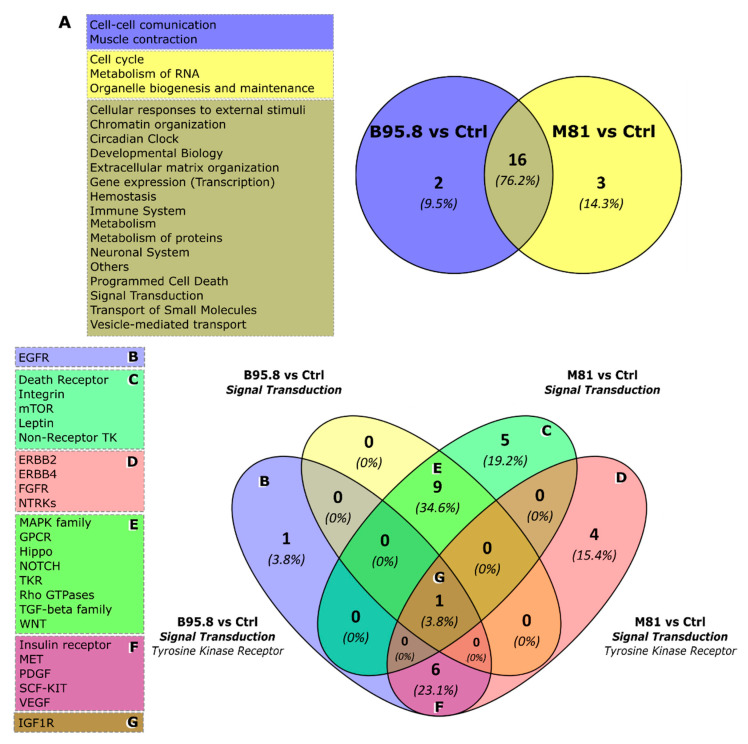
Results of in silico pathway enrichment analysis from predicted target genes of deregulated miRNAs in NP69SV40 cells transfected with LMP1 B95.8, LMP1 M81 or the control vector. (**A**) Unique primary categories for EBV LMP1 B95.8 vs. Ctrl (in blue), LMP1 M81 vs. Ctrl (yellow), and shared categories for by LMPs B95.8 and M81 (overlap). (**B**) Unique “Tyrosine kinase receptor” categories from B95.8 vs. Ctrl comparison. (**C**) Unique “Signal transduction” categories from M81 vs. Ctrl comparison. (**D**) Unique “Tyrosine kinase receptor” categories from M81 vs. Ctrl comparison. (**E**) “Signal transduction” categories shared between B95.8 vs. Ctrl and M81 vs. Ctrl comparisons. (**F**) “Tyrosine kinase receptor” categories shared between B95.8 vs. Ctrl and M81 vs. Ctrl comparisons. (**G**) Category shared between B95.8 vs. Ctrl and M81 vs. Ctrl comparisons, present in “Signal transduction” and Unique “Tyrosine kinase receptor” pathways.

**Figure 5:**
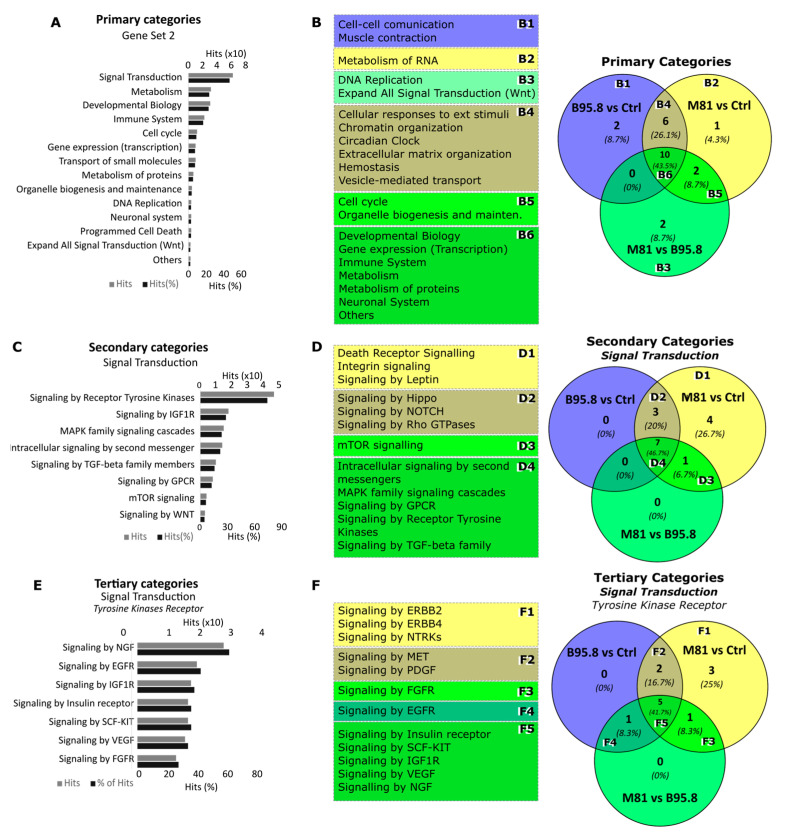
Pathway enrichment analysis of genes targeted by miRNAs deregulated by EBV LMP1 in NP69SV40T cells. Most important primary categories altered by predicted genes comparing LMP1 M81 vs. B95.8. (**B**) Unique and commonly deregulated primary categories between the three comparisons performed. (**C**) “Signal transduction” secondary categories found for LMP1 M81 vs. B95.8. (**D**) Unique and commonly deregulated secondary categories inside “Signal Transduction” for the three comparisons performed. (**E**) Subcategories (tertiary) for “Tyrosine Kinases Receptor” altered by predicted genes comparing LMP1 M81 vs. B95.8. (**F**) Unique and commonly deregulated tertiary categories for “Tyrosine Kinases Receptor” considering the three comparisons performed. In (**A**,**C**,**E**), in light and dark grey indicates, respectively, the absolute number of hits or its percentage considering the respective higher category (Gene set #2, in the case of primary categories depicted in (**A**)).

The pathway enrichment analysis was used to obtain insights on the most relevant cellular pathways disturbed by the regulation of miRNAs by EBV LMP1. We also evaluated whether a given pathway identified was unique to a given comparison, aiming to identify processes regulated specifically by LMP1 derived from viral strain B95.8 or M81. In the B95.8 vs. Ctrl comparison set, we found pathways for genes involved in cell–cell communication, such as integrins and cadherins (Figure 3B, Figure 4A and Figure 5A), while the M81 vs. Ctrl comparison showed pathways featuring genes involved in the metabolism of RNA (RNA processing), such as *NCBP1*, *NUP43*, *NUP58*, *POM121,* and *RANBP2* (gene IDs in Supplementary Table S8) (Figure 3B, Figure 4A and Figure 5A, and Supplementary Table S7). Furthermore, Death receptors, Integrin and Leptin signaling pathways were found for comparison M81 vs. Ctrl; those three pathways have the gene *SOS1* in common, which encodes a protein that promotes the exchange of Ras-bound GDP by GTP, favoring cell proliferation. Additionally, the M81 vs. B95.8 comparison showed some unique predicted pathways, including involvement in DNA replication through genes *CDC7*, *ORC4*, *MCM10*, and *MCM9* (Figure 5B).

Both comparison groups involving EBV LMP1 variant M81 (M81 vs. Ctrl/M81 vs. B95.8) were predicted to be uniquely involved in the cell cycle (via *RB1*) and organelle biogenesis and maintenance (via *GABPB1* and *PRKAA2*) (Figure 5A, Figure 5(B5) and Figure S7). Additionally, the LMP1 from the M81 strain was predicted to be involved in mTOR signaling (Figure 3D and Figure 5(D3)), via genes *PRKAA2* and *PPM1A,* for instance. All three comparison sets were predicted to regulate the Wnt signaling through different gene sets, but genes *FZD5* and *CAV1* appear to be regulated in all settings (Figure 4E and Figure 5(B3)). The comparisons M81 vs. Ctrl and M81 vs. B95.8 also were predicted to regulate pathways involved in chromatin organization, extracellular matrix organization, program cell death, vesicle-mediated transport, metabolism of proteins, gene transcription, immune system regulation, and important pathways in cancer, such as MAPK, TGF-β, WNT, VEGF, and IGF1R (Figure 3D, Figure 4E–G and Figure 5).

## 4. Discussion

There is compelling evidence showing that the viral LMP1 oncoprotein contributes to the progression of EBV-associated cancers [14]. For instance, LMP1 increases migration and invasion of epithelial cells through different mechanisms, such as changes in cell adhesion and motility due to regulation of N-cadherin and integrin-α5 expression, culminating in both individual and collective migration of immortalized nasopharyngeal cells [42]. LMP1 also directly increases the sphingosine kinase 1 (SPHK1) enzyme, which was implicated in a poor prognosis for NPC [43]. SPHK1 activates sphingosine-1-phosphate (S1P), causing increased migration of NPC cells associated with AKT activation [44]. Furthermore, LMP1 represses the Tissue Inhibitor of Metalloproteinase-3 (TIMP-3), leading to extracellular matrix degradation [45], and induces extracellular secretion of HIF1α in exosomes, which ultimately causes epithelial-to-mesenchymal transition (EMT), migration and invasion of EBV-negative nasopharyngeal cells, and NPC [46].

In this study, we found that the expression of LMP1 derived from EBV strains B95.8 and M81 in immortalized nasopharyngeal cells NP69^SV40T^ changes the expression of endogenous miRNAs. Cells expressing LMP1 variant M81 compared to variant B95.8 showed significant upregulation of the human miRNAs 100-5p, 192-3p, and 574-3p (Figure 2C). MiR-100-5p was previously described to behave either as a tumor suppressor or oncomir, in a context-dependent manner. The upregulation of miR-100-5p was implicated on better prognosis in esophageal cancer [47], a decrease in cisplatin resistance in lung cancer [48], and inhibition of tumorigenesis, cell migration, and invasion for human mammary epithelial cells [49]. However, it was also associated with effects expected to favor cancer development and progression, such as resistance against apoptosis in prostate cancer [50] and induction of EMT in human mammary cells [49]. MiR-192-5p seems to behave as a more typical oncomir: its expression stimulates migration, invasion, and proliferation of hepatocellular carcinoma cells in vitro [51], and it was associated with tamoxifen resistance in mammary carcinoma and even higher cancer recurrence and metastasis in both hepatocellular and mammary carcinoma [51,52]. Conversely, miR-574-3p was described with effects mimicking those of tumor suppressor genes. For instance, it was implicated in inhibition of gastric cancer cell proliferation, migration, and invasion [53]. However, its role in NPC still needs elucidation since miRNA effects can be tissue- and context-dependent.

The putative cellular effects can be appreciated by the results obtained by miRNA target prediction and the pathway-enrichment analysis, both performed in silico. The biological processes involved (Figure 4 and Figure 5) include gene expression (e.g., chromatin organization, RNA-pol II transcription, and post-translational protein modification), intracellular signaling pathways (e.g., programmed cell death, tyrosine kinase receptors, MAPK, TGF-β, and Rho GTPases signaling), cellular stress and senescence, modulation of the immune system, and phenomena associated to cell–cell communication (e.g., ECM organization and vesicle transport). This is consistent with previously published data about a range of effects associated with EBV LMP1 expression, including the induction of vasculogenic mimicry in vitro via VEGFA; induction of IGF1 expression and cell proliferation in vitro; MAPK pathway regulation, leading to cell motility and invasion; blocking of TGF1 cell-growth inhibition in vitro; and Wnt pathway regulation, both in mice and in human tumor samples of EBV-positive NPC [54,55,56,57,58,59].

Some common features were observed when evaluating the effects of LMP1 expression on cellular miRNAs, irrespective of the variant considered (B95.8 vs. Ctrl and M81 vs. Ctrl comparisons). For instance, EBV LMP1 can downregulate miRNAs that are implicated in epigenetic regulation by targeting DNA methyltransferases (DNMTs) genes. It was previously found that EBV-infected cells show downregulation of DNMT1 and upregulation of DNMT3a, admittedly due to LMP1 expression [60,61]. We found that the transcript for DNMT3a is targeted by 10 miRNAs in B95.8 vs. Ctrl, by miR-203a-3p in M81 vs. Ctrl, and by miR-192-5p in M81 vs. B95.8 comparison sets. The downregulation of DNMT3a reduces DNA methylation in specific genomic regions, increasing the expression of *FOXA2* and *HNF4A* (Gene IDs in Supplementary Table S8) [62]. We found that *FOXA2* is targeted by miR-141-3p, while *HNF4A* is targeted by miR-135b-5p and miR-34c-5p; these three human miRNAs were found to be downregulated in cells expressing the EBV LMP1 variant B95.8, compared to control (B95.8 vs. Ctrl). *FOXA2* was previously implicated in cell proliferation, cancer stem cell maintenance, and an increase in relapse in triple-negative breast cancer [63], while *HNF4A* was related to an increase in lymph node and distant metastasis in colon cancer [64,65]. Since all those miRNAs are downregulated in the presence of LMP1, it is expected that the above-described effects will increase, suggesting that LMP1 from both EBV strains B95.8 and M81 regulates the methylation status in cells using a different set of miRNAs.

Even though this study provides relevant clues on common and unique effects features of the EBV LMP1 derived from viral strains M81 and B95.8 in nasopharyngeal cells, some limitations must be carefully considered. First, the model used in this study was based on EBV LMP1 expression in NP69^SV40T^ cells under the control of a CMV promoter, which allows robust ectopic expression of the viral transgene. We initially sought to generate stably transfected cells expressing EBV LMP1, but only transient expression was possible because cells constitutively expressing LMP1 show a high level of cell death after a few weeks. Despite its known antiapoptotic effects, at unconstrained high levels, the EBV LMP1 may actually cause the cell to die by apoptosis [66,67,68]. To circumvent this issue and minimize the detrimental effects of suboptimal transfection efficiencies, we performed enrichment of LMP1-expressing cells by FACS prior to miRNA expression analysis. Another limitation is that our results based on in silico analysis were not validated experimentally in this study, but other ongoing studies in our laboratory aim to address this. Finally, we observed a much higher number of altered miRNAs when evaluating cells expressing LMP1 B95.8 compared to cells expressing LMP1 M81. As the miRNA panel in this study was defined based on previously published results and considering that there are much more data accumulated for EBV genotype B95.8 compared to M81, we cannot rule out some bias towards the results for miRNAs regulated by B95.8, which is the most studied EBV genotype. Nevertheless, we aimed to reduce any possible bias in this matter by also including in the panel miRNAs reported as altered in cancers in general, not only associated with EBV, and considering both in vitro and in vivo studies.

Despite these limitations, the results obtained consistently showed different profiles of miRNA expression induced by LMP1 derived from viral strains B95.8 and M81 in our model. This allowed us to identify the miRs 100-5p, 192-5p, and 574-3p, as microRNAs with putative roles in the EBV-induced transformation of nasopharyngeal epithelial cells. The LMP1 from EBV strains B95.8 and M81 regulate different miRNA sets, and the data obtained from the in silico analysis suggested putative biological consequences, either some unique for one of the LMP1 variants, but also commonalities, such as changes in cellular pathways involving MAPKs and VEGFA, modulation of the immune system, and apoptosis. Of note, it was previously reported that the EBV strain M81 has a higher capacity to induce the lytic cycle in infected cells [20]. Accordingly, the target gene prediction and pathway enrichment analysis performed in silico in this study indicated that, compared to B95.8, the LMP1 variant M81 had a higher number of genes involved in cell death and survival regulation, suggesting that, to some extent, the M81 biological behavior may be related to its EBV LMP1 variant and the effects of this viral oncoprotein on modulation of cellular miRNAs.

## 5. Conclusions

This study showed that LMP1 derived from EBV strains B95.8 and M81 can modulate different sets of miRNAs when expressed in NP69 nasopharyngeal cells. The results reported here contribute to a better understanding of how LMP1 from different viral strains may influence the behavior and phenotype of EBV-infected cells, and also indicate novel putative genes and cellular pathways that may play an important role in the pathogenesis of cancers associated with EBV. These differentially expressed miRNAs can also have a role in NPC diagnosis or management since these molecules are known to be found in plasma samples. However, the LMP1 effects on the regulation of endogenous miRNAs are still poorly explored; future studies may focus on how specific miRNAs deregulated by LMP1 affect the cell signaling pathways, which is key to further clarifying the biological and oncogenic properties of this major EBV oncoprotein.

## Data Availability

The authors confirm that the data supporting the findings of this study are available within the article and its Supplementary Materials.

## Supplementary Materials

The following supporting information can be downloaded at: https://www.mdpi.com/article/10.3390/genes13020353/s1, Figure S1: Comercial vector pZsGreen after the LMP1 gene insertion (dark green). The vector was assembled through conventional cloning to access differences between LMP1 variants from EBV in selected miRNAs expression after transient transfection in immortalized nasopharyngeal cells (NP69); Figure S2: Analysis of LMP1 expression and GFP positivity in HEK293 or NP69 cells after 48h of transfection with the assembled constructs pZsG-LMP1-B95.8 or pZsG-LMP1-M81. PCR amplification of cDNA from (A) HEK293 cells expressing LMP1 from the construct pZsG-LMP1-B95.8 and pZsG-LMP1-M81 and (B) transfected NP69 cells, indicating the 30bp deletion present in LMP1 variant from M81 strain. expressing LMP1 from the construct pZsG-LMP1-B95.8 and pZsG-LMP1-M81. (C) Example of transfection rates in HEK293 and NP69 cells transfected with pZsGreen vector; Figure S3: FACS of NP69SV40 cells after 48h of transient transfection with pZsGreen (control), pZsG-LMP1-B95.8 or pZsG-LMP1-M81 vectors; Figure S4: Schematic drawing of steps followed after transfection of NP69SV40 cells transfected with pZsGreen (control), pZsG-LMP1-B95.8 or pZsG-LMP1-M81 vectors. and sorted by FACS with pZsGreen vector as control or containing the coding region from LMP1 protein from EBV variants B95.8 or M81. First, 48 h after transfection, GFP positive cells were sorted by FACs, followed by RNA extraction, cDNA production and RT-qPCR array of 91 pre-selected miRNAs. Then, target prediction analysis of differentially expressed miRNAs and genes with high scores were selected for Pathway enrichment analysis; Figure S5: Analysis of target genes from differentially expressed miRNAs in NP69SV40T cells transfected with EBV LMP1 from B95.8 or M81 variants. Example of primary, secondary and tertiary categories organization. Panels C, D and E show results of in silico pathway enrichment analysis of predicted target genes from deregulated miRNAs in NP69SV40 cells transfected with LMP1 B95.8, LMP1 M81 or the control vector; Table S1: Primers sequences, reaction components and cycling utilized for checking LMP1 presence, sequencing and cloning; Table S2: MiRNAs related to NPC, LMP1, EBV, or cancer in general, selected from literature separated by its probable function as tumor suppressor, oncomiR or dual function. Those miRNAs were used in the qPCR miRNA array to analyze their expression in NP69 cells transfected with LMP1 from two distinct EBV strains, B95.8A or M81; Table S3: Results (Cell count and percentage) from FACS of NP69SV40 cells after 48h of transient transfection with pZsGreen (control), pZsG-LMP1-B95.8 or pZsG-LMP1-M81 vectors; Table S4: Raw data (CT) after qPCR array using cDNA from NP69SV40 cells transfected with pZsGreen (control), pZsG-LMP1-B95.8 or pZsG-LMP1-M81 vectors and sorted by FACS; Table S5: Results of RT-qPCR arrays analysis and verification of up or downregulated miRNAs of NP69SV40 cells transfected with pZsGreen (control), pZsG-LMP1-B95.8 or pZsG-LMP1-M81 vectors and sorted by FACS; Table S6: Analysis of pathway enrichment analysis using predicted genes from gene set #2 targeted by differentially expressed miRNAs from NP69SV40 cells transfected with pZsGreen (control), pZsG-LMP1-B95.8 or pZsG-LMP1-M81 vectors and sorted by FACS. Each group has pathways represented in hits (gene in each pathway) and % hits (percentage of hits compared to next higher category); Table S7: Descriptive list of predicted genes found by mirDIP encountered in each primary category after pathway enrichment analysis using differentially expressed miRNAs from NP69SV40 cells transfected with pZsGreen (control), pZsG-LMP1-B95.8 or pZsG-LMP1-M81 vectors and sorted by FACS; Table S8: List of Gene IDs cited on main text. Predicted genes were from differentially expressed miRNAs of transfected immortalized nasopharyngeal cells with LMP1 variant from EBV strain B95.8 or M81.

## References

[1] Global Burden of Disease Cancer Collaboration (2015). The Global Burden of Cancer 2013. JAMA Oncol..

[2] Oh J.-K., Weiderpass E. (2014). Infection and Cancer: Global Distribution and Burden of Diseases. Ann. Glob. Health.

[3] Schottenfeld D., Beebe-Dimmer J. (2015). The Cancer Burden Attributable to Biologic Agents. Ann. Epidemiol..

[4] Stewart B.W., Wild C.P. (2014). World Cancer Report 2014. http://www.iahm.org/journal/vol_15/num_3/text/vol15n3p40.pdf.

[5] De Martel C., Ferlay J., Franceschi S., Vignat J., Bray F., Forman D., Plummer M. (2012). Global Burden of Cancers Attributable to Infections in 2008: A Review and Synthetic Analysis. Lancet Oncol..

[6] Vineis P., Wild C.P. (2014). Global Cancer Patterns: Causes and Prevention. Lancet.

[7] Dunmire S.K., Hogquist K.A., Balfour H.H., Münz C. (2015). Infectious Mononucleosis. Epstein Barr Virus Volume 1.

[8] Elgui de Oliveira D. (2007). DNA Viruses in Human Cancer: An Integrated Overview on Fundamental Mechanisms of Viral Carcinogenesis. Cancer Lett..

[9] Tang L.-L., Chen W.-Q., Xue W.-Q., He Y.-Q., Zheng R.-S., Zeng Y.-X., Jia W.-H. (2016). Global Trends in Incidence and Mortality of Nasopharyngeal Carcinoma. Cancer Lett..

[10] Colditz G.A., Wolin K.Y., Gehlert S. (2012). Applying What We Know to Accelerate Cancer Prevention. Sci. Transl. Med..

[11] Brousset P., Keryer C., Ooca T., Corbex M. (2004). EBV-Associated Nasopharyngeal Carcinomas: From Epidemiology to Virus-Targeting Strategies. Trends Microbiol..

[12] The Enigmatic Epidemiology of Nasopharyngeal Carcinoma|Cancer Epidemiology, Biomarkers & Prevention. http://cebp.aacrjournals.org/content/15/10/1765.long.

[13] Tsao S.-W., Tsang C.M., To K.-F., Lo K.-W. (2015). The Role of Epstein–Barr Virus in Epithelial Malignancies: Role of EBV in Epithelial Malignancies. J. Pathol..

[14] De Oliveira D.E., Müller-Coan B.G., Pagano J.S. (2016). Viral Carcinogenesis Beyond Malignant Transformation: EBV in the Progression of Human Cancers. Trends Microbiol..

[15] Brinkmann M.M. (2006). Regulation of Intracellular Signalling by the Terminal Membrane Proteins of Members of the Gammaherpesvirinae. J. Gen. Virol..

[16] Lin X., Tang M., Tao Y., Li L., Liu S., Guo L., Li Z., Ma X., Xu J., Cao Y. (2012). Epstein–Barr Virus-Encoded LMP1 Triggers Regulation of the ERK-Mediated Op18/Stathmin Signaling Pathway in Association with Cell Cycle. Cancer Sci..

[17] Masoud G.N., Li W. (2015). HIF-1α Pathway: Role, Regulation and Intervention for Cancer Therapy. Acta Pharm. Sin. B.

[18] Wakisaka N., Kondo S., Yoshizaki T., Murono S., Furukawa M., Pagano J.S. (2004). Epstein–Barr Virus Latent Membrane Protein 1 Induces Synthesis of Hypoxia-Inducible Factor 1. Mol. Cell. Biol..

[19] Tsai M.-H., Lin X., Shumilov A., Bernhardt K., Feederle R., Poirey R., Kopp-Schneider A., Pereira B., Almeida R., Delecluse H.-J. (2016). The Biological Properties of Different Epstein–Barr Virus Strains Explain Their Association with Various Types of Cancers. Oncotarget.

[20] Tsai M.-H., Raykova A., Klinke O., Bernhardt K., Gärtner K., Leung C.S., Geletneky K., Sertel S., Münz C., Feederle R. (2013). Spontaneous Lytic Replication and Epitheliotropism Define an Epstein–Barr Virus Strain Found in Carcinomas. Cell Rep..

[21] Abba M., Patil N., Allgayer H. (2014). MicroRNAs in the Regulation of MMPs and Metastasis. Cancers.

[22] Acunzo M., Romano G., Wernicke D., Croce C.M. (2015). MicroRNA and Cancer—A Brief Overview. Adv. Biol. Regul..

[23] Abba M.L., Patil N., Leupold J.H., Allgayer H. (2016). MicroRNA Regulation of Epithelial to Mesenchymal Transition. J. Clin. Med..

[24] Chen H.-C., Chen G.-H., Chen Y.-H., Liao W.-L., Liu C.-Y., Chang K.-P., Chang Y.-S., Chen S.-J. (2009). MicroRNA Deregulation and Pathway Alterations in Nasopharyngeal Carcinoma. Br. J. Cancer.

[25] Yang G.-D., Huang T.-J., Peng L.-X., Yang C.-F., Liu R.-Y., Huang H.-B., Chu Q.-Q., Yang H.-J., Huang J.-L., Zhu Z.-Y. (2013). Epstein–Barr Virus_Encoded LMP1 Upregulates MicroRNA-21 to Promote the Resistance of Nasopharyngeal Carcinoma Cells to Cisplatin-Induced Apoptosis by Suppressing PDCD4 and Fas-L. PLoS ONE.

[26] Uhlmann S., Zhang J.D., Schwäger A., Mannsperger H., Riazalhosseini Y., Burmester S., Ward A., Korf U., Wiemann S., Sahin O. (2010). MiR-200bc/429 Cluster Targets PLCgamma1 and Differentially Regulates Proliferation and EGF-Driven Invasion than MiR-200a/141 in Breast Cancer. Oncogene.

[27] Gu X., Li J.-Y., Guo J., Li P.-S., Zhang W.-H. (2015). Influence of MiR-451 on Drug Resistances of Paclitaxel-Resistant Breast Cancer Cell Line. Med. Sci. Monit. Int. Med. J. Exp. Clin. Res..

[28] Raza U., Zhang J.D., Sahin O. (2014). MicroRNAs: Master Regulators of Drug Resistance, Stemness, and Metastasis. J. Mol. Med. Berl. Ger..

[29] Calin G.A., Dumitru C.D., Shimizu M., Bichi R., Zupo S., Noch E., Aldler H., Rattan S., Keating M., Rai K. (2002). Frequent Deletions and Down-Regulation of Micro- RNA Genes MiR15 and MiR16 at 13q14 in Chronic Lymphocytic Leukemia. Proc. Natl. Acad. Sci. USA.

[30] Graham F.L., Smiley J., Russell W.C., Nairn R. (1977). Characteristics of a Human Cell Line Transformed by DNA from Human Adenovirus Type 5. J. Gen. Virol..

[31] Tsao S.W., Wang X., Liu Y., Cheung Y.C., Feng H., Zheng Z., Wong N., Yuen P.W., Lo A.K., Wong Y.C. (2002). Establishment of Two Immortalized Nasopharyngeal Epithelial Cell Lines Using SV40 Large T and HPV16E6/E7 Viral Oncogenes. Biochim. Biophys. Acta BBA-Mol. Cell Res..

[32] Delecluse H.-J., Hilsendegen T., Pich D., Zeidler R., Hammerschmidt W. (1998). Propagation and Recovery of Intact, Infectious Epstein–Barr Virus from Prokaryotic to Human Cells. Proc. Natl. Acad. Sci. USA.

[33] Feederle R., Bartlett E.J., Delecluse H.-J. (2010). Epstein–Barr Virus Genetics: Talking about the BAC Generation. Herpesviridae.

[34] Keller S.A., Hernandez-Hopkins D., Vider J., Ponomarev V., Hyjek E., Schattner E.J., Cesarman E. (2006). NF-ΚB Is Essential for the Progression of KSHV- and EBV-Infected Lymphomas in Vivo. Blood.

[35] Gebäck T., Shultz M.M.P., Koumoutsakos P., Detmar M. (2009). TScratch: A Novel and Simple Software Tool for Automated Analysis of Monolayer Wound Healing Assays. BioTechniques.

[36] Marí-Alexandre J., Barceló-Molina M., Belmonte-López E., García-Oms J., Estellés A., Braza-Boïls A., Gilabert-Estellés J. (2018). Micro-RNA Profile and Proteins in Peritoneal Fluid from Women with Endometriosis: Their Relationship with Sterility. Fertil. Steril..

[37] Ferrante S.C., Nadler E.P., Pillai D.K., Hubal M.J., Wang Z., Wang J.M., Gordish-Dressman H., Koeck E., Sevilla S., Wiles A.A. (2015). Adipocyte-Derived Exosomal MiRNAs: A Novel Mechanism for Obesity-Related Disease. Pediatr. Res..

[38] Tokar T., Pastrello C., Rossos A.E.M., Abovsky M., Hauschild A.-C., Tsay M., Lu R., Jurisica I. (2018). MirDIP 4.1—Integrative Database of Human MicroRNA Target Predictions. Nucleic Acids Res..

[39] Shirdel E.A., Xie W., Mak T.W., Jurisica I. (2011). NAViGaTing the Micronome—Using Multiple MicroRNA Prediction Databases to Identify Signalling Pathway-Associated MicroRNAs. PLoS ONE.

[40] Croft D., Mundo A.F., Haw R., Milacic M., Weiser J., Wu G., Caudy M., Garapati P., Gillespie M., Kamdar M.R. (2014). The Reactome Pathway Knowledgebase. Nucleic Acids Res..

[41] Shannon P., Markiel A., Ozier O., Baliga N.S., Wang J.T., Ramage D., Amin N., Schwikowski B., Ideker T. (2003). Cytoscape: A Software Environment for Integrated Models of Biomolecular Interaction Networks. Genome Res..

[42] Wasil L.R., Shair K.H.Y. (2015). Epstein–Barr Virus LMP1 Induces Focal Adhesions and Epithelial Cell Migration through Effects on Integrin-A5 and N-Cadherin. Oncogenesis.

[43] Li W., Tian Z., Qin H., Li N., Zhou X., Li J., Ni B., Ruan Z. (2015). High Expression of Sphingosine Kinase 1 Is Associated with Poor Prognosis in Nasopharyngeal Carcinoma. Biochem. Biophys. Res. Commun..

[44] Lee H.M., Lo K.-W., Wei W., Tsao S.W., Chung G.T.Y., Ibrahim M.H., Dawson C.W., Murray P.G., Paterson I.C., Yap L.F. (2017). Oncogenic S1P Signalling in EBV-Associated Nasopharyngeal Carcinoma Activates AKT and Promotes Cell Migration through S1P Receptor 3. J. Pathol..

[45] Chang S.-H., Chang H.-C., Hung W.-C. (2008). Transcriptional Repression of Tissue Inhibitor of Metalloproteinase-3 by Epstein–Barr Virus Latent Membrane Protein 1 Enhances Invasiveness of Nasopharyngeal Carcinoma Cells. Oral Oncol..

[46] Aga M., Bentz G.L., Raffa S., Torrisi M.R., Kondo S., Wakisaka N., Yoshizaki T., Shackelford J. (2014). Exosomal HIF1α Supports Invasive Potential of Nasopharyngeal Carcinoma-Associated LMP1-Positive Exosomes. Oncogene.

[47] Zhang H.-C., Tang K.-F. (2017). Clinical Value of Integrated-Signature MiRNAs in Esophageal Cancer. Cancer Med..

[48] Qin X., Yu S., Zhou L., Shi M., Hu Y., Xu X., Shen B., Liu S., Yan D., Feng J. (2017). Cisplatin-Resistant Lung Cancer Cell–Derived Exosomes Increase Cisplatin Resistance of Recipient Cells in Exosomal MiR-100–5p-Dependent Manner. Int. J. Nanomed..

[49] Chen D., Sun Y., Yuan Y., Han Z., Zhang P., Zhang J., You M.J., Teruya-Feldstein J., Wang M., Gupta S. (2014). MiR-100 Induces Epithelial-Mesenchymal Transition but Suppresses Tumorigenesis, Migration and Invasion. PLoS Genet..

[50] Nabavi N., Saidy N.R.N., Venalainen E., Haegert A., Parolia A., Xue H., Wang Y., Wu R., Dong X., Collins C. (2017). MiR-100-5p Inhibition Induces Apoptosis in Dormant Prostate Cancer Cells and Prevents the Emergence of Castration-Resistant Prostate Cancer. Sci. Rep..

[51] Yan-Chun L., Hong-Mei Y., Zhi-Hong C., Qing H., Yan-Hong Z., Ji-Fang W. (2015). MicroRNA-192-5p Promote the Proliferation and Metastasis of Hepatocellular Carcinoma Cell by Targeting SEMA3A. Appl. Immunohistochem. Mol. Morphol..

[52] Kim Y.S., Park S.J., Lee Y.S., Kong H.K., Park J.H. (2016). MiRNAs Involved in LY6K and Estrogen Receptor α Contibute to Tamoxifen-Suceptibility in Breast Cancer. Oncotarget.

[53] Su Y., Ni Z., Wang G., Cui J., Wei C., Wang J., Yang Q., Xu Y., Li F. (2012). Aberrant Expression of MicroRNAs in Gastric Cancer and Biological Significance of MiR-574-3p. Int. Immunopharmacol..

[54] Xu S., Bai J., Zhuan Z., Li B., Zhang Z., Wu X., Luo X., Yang L. (2018). EBV-LMP1 Is Involved in Vasculogenic Mimicry Formation via VEGFA/VEGFR1 Signaling in Nasopharyngeal Carcinoma. Oncol. Rep..

[55] Tworkoski K., Raab-Traub N. (2014). LMP1 Promotes Expression of Insulin-Like Growth Factor 1 (IGF1) To Selectively Activate IGF1 Receptor and Drive Cell Proliferation. J. Virol..

[56] Dawson C.W., Laverick L., Morris M.A., Tramoutanis G., Young L.S. (2008). Epstein–Barr Virus-Encoded LMP1 Regulates Epithelial Cell Motility and Invasion via the ERK-MAPK Pathway. J. Virol..

[57] Fukuda M., Kurosaki W., Yanagihara K., Kuratsune H., Sairenji T. (2002). A Mechanism in Epstein–Barr Virus Oncogenesis: Inhibition of Transforming Growth Factor-Β1-Mediated Induction of MAPK/P21 by LMP1. Virology.

[58] QingLing Z., LiNa Y., Liu L., Shuang W., YuFang Y., Yi D., Divakaran J., Xin L., YanQing D. (2011). LMP1 Antagonizes WNT/β-Catenin Signalling through Inhibition of WTX and Promotes Nasopharyngeal Dysplasia but Not Tumourigenesis in LMP1B95−8 Transgenic Mice. J. Pathol..

[59] Zeng Z.-Y., Zhou Y.-H., Zhang W.-L., Xiong W., Fan S.-Q., Li X.-L., Luo X.-M., Wu M.-H., Yang Y.-X., Huang C. (2007). Gene Expression Profiling of Nasopharyngeal Carcinoma Reveals the Abnormally Regulated Wnt Signaling Pathway. Hum. Pathol..

[60] Leonard S., Wei W., Anderton J., Vockerodt M., Rowe M., Murray P.G., Woodman C.B. (2011). Epigenetic and Transcriptional Changes Which Follow Epstein–Barr Virus Infection of Germinal Center B Cells and Their Relevance to the Pathogenesis of Hodgkin’s Lymphoma. J. Virol..

[61] Seo S.Y., Kim E.-O., Jang K.L. (2008). Epstein–Barr Virus Latent Membrane Protein 1 Suppresses the Growth-Inhibitory Effect of Retinoic Acid by Inhibiting Retinoic Acid Receptor-Β2 Expression via DNA Methylation. Cancer Lett..

[62] Liao J., Karnik R., Gu H., Ziller M.J., Clement K., Tsankov A.M., Akopian V., Gifford C.A., Donaghey J., Galonska C. (2015). Targeted Disruption of DNMT1, DNMT3A and DNMT3B in Human Embryonic Stem Cells. Nat. Genet..

[63] Perez-Balaguer A., Ortiz-Martínez F., García-Martínez A., Pomares-Navarro C., Lerma E., Peiró G. (2015). FOXA2 MRNA Expression Is Associated with Relapse in Patients with Triple-Negative/Basal-like Breast Carcinoma. Breast Cancer Res. Treat..

[64] Yao H.S., Wang J., Zhang X.P., Wang L.Z., Wang Y., Li X.X., Jin K.Z., Hu Z.Q., Wang W.J. (2016). Hepatocyte Nuclear Factor 4α Suppresses the Aggravation of Colon Carcinoma. Mol. Carcinog..

[65] Zhao J., Adams A., Roberts B., O’Neil M., Vittal A., Schmitt T., Kumer S., Cox J., Li Z., Weinman S.A. (2018). Protein Arginine Methyl Transferase 1– and Jumonji C Domain-Containing Protein 6–Dependent Arginine Methylation Regulate Hepatocyte Nuclear Factor 4 α Expression and Hepatocyte Proliferation in Mice. Hepatology.

[66] Clorennec C.L., Ouk T.-S., Youlyouz-Marfak I., Panteix S., Martin C.-C., Rastelli J., Adriaenssens E., Zimber-Strobl U., Coll J., Feuillard J. (2008). Molecular Basis of Cytotoxicity of Epstein–Barr Virus (EBV) Latent Membrane Protein 1 (LMP1) in EBV Latency III B Cells: LMP1 Induces Type II Ligand-Independent Autoactivation of CD95/Fas with Caspase 8-Mediated Apoptosis. J. Virol..

[67] Clorennec C.L., Youlyouz-Marfak I., Adriaenssens E., Coll J., Bornkamm G.W., Feuillard J. (2006). EBV Latency III Immortalization Program Sensitizes B Cells to Induction of CD95-Mediated Apoptosis via LMP1: Role of NF-ΚB, STAT1, and P53. Blood.

[68] Zhang X., Uthaisang W., Hu L., Ernberg I.T., Fadeel B. (2005). Epstein–Barr Virus-Encoded Latent Membrane Protein 1 Promotes Stress-Induced Apoptosis Upstream of Caspase-2-Dependent Mitochondrial Perturbation. Int. J. Cancer.

