# Supplementary material for: Latent Membrane Protein 1 (LMP1) from Epstein–Barr Virus (EBV) Strains M81 and B95.8 Modulate miRNA Expression When Expressed in Immortalized Human Nasopharyngeal Cells
Source: Genes (Basel). 2022 Feb 16;13(2):353. doi: 10.3390/genes13020353 (PMC8871543; doi:10.3390/genes13020353)
Supplement: Supplementary file 1 [file genes-13-00353-s001.zip › genes-1531882-supplementary.pdf]

Latent Membrane Protein 1 (LMP1) from Epstein–Barr Virus (EBV) Strains M81 and B95.8 Modulate miRNA Expression When Expressed in Immortalized Human Nasopharyngeal Cells

Barbara G. Müller Coan [1], Ethel Cesarman [2], Marcio Luis Acencio [3] and Deilson Elgui de Oliveira [4,5,*]

[1] Biosciences Institute of Botucatu, São Paulo State University (UNESP), Botucatu 18618-689, SP, Brazil; barbaramcoan@gmail.com
[2] Department of Pathology and Laboratory Medicine, Weill Cornell Medicine, New York, NY 10065, USA; ecesarm@med.cornell.edu
[3] Luxembourg Centre for Systems Biomedicine (LCSB), University of Luxembourg, L-4367 Belvaux, Luxembourg; marcio.acencio@uni.lu
[4] Medical School, São Paulo State University (UNESP), Botucatu 18618-687, SP, Brazil
[5] Institute for Biotechnology (IBTEC), São Paulo State University (UNESP), Botucatu 18607-440, SP, Brazil
* Correspondence: deilson.elgui@unesp.br; Tel.: +55-14-3880-1573

Supplementary Table S1 – Primers sequences, reaction components and cycling utilized for checking LMP1 presence, sequencing and cloning.

Targets and primers	Reaction components	Cycling
**Primer for LMP1 DNA or cDNA distinction**<br>Sense: CGT TAT GAG TGA CTG GAC TGG A<br>Antisense: TGA ACA GCA CAA TTC CAA GG	PCR Buffer 1x (Tris-HCl100mM pH 8,5/ 500mM KCl), 2,5mM MgCl, 0,20mM dNTP, 0,30mM of each primer, 1U DNA Polimerase	96°C- 5min (1x); 94°C-30s, 57°C - 40s, 72°C-40s (30x); 72°C- 10min; 4°C - ∞
**LMP1 hole sequence**<br>Sense: ATG GAA CRC GAC CTT<br>Antisense: TTA GTC ATA GTA GCT TAG CT	PCR Buffer 1x (Tris-HCl100mM pH 8,5/ 500mM KCl), 2,5mM MgCl, 0,20mM dNTP, 0,30mM of each primer, 1U DNA Polimerase	96°C- 5min (1x); 94°C-30s, 55°C - 40s, 72°C-2min (37x); 72°C- 10min; 4°C - ∞
**LMP1 hole sequence (clonning purposes)**<br>EBVLMP1_EcoRI.S: GCG CGC **G↓AA TTC** CGT ACT GCC TCC GGC AGA<br>EBVLMP1_BamHI.A: GCG CGC **G↓GA TCC** AAT GTG GCT TTT CAG CCT A	PCR Buffer 1x (Tris-SO4 60mM pH 8,9/sulfato de amônio 18mM), 2,5mM MgSO4, 0,20mM dNTP, 0,30mM of each primer, 1U DNA polimerase (TaqDNA Polimerase High Fidelity).	96°C- 5min (1x); 94°C-30s, 55°C - 40s, 72°C-2min (37x); 72°C- 10min; 4°C - ∞
**Verification of insert in pZsGreen plasmid**<br>EF1a.S: TCA AGC CTC AGA CAG TGG TTC<br>IRES.A: ACG CAC ACC GGC CTT AT	PCR Buffer 1x (Tris-HCl100mM pH 8,5/ 500mM KCl), 2,5mM MgCl, 0,20mM dNTP, 0,30mM of each primer, 1U DNA Polimerase	96°C- 5min (1x); 94°C-30s, 55°C - 40s, 72°C-2min (37x); 72°C- 10min; 4°C - ∞
**Verification os insert in pGEM TEasy plasimd**<br>pGEM TF: GTT TTC CCA GTC ACG AC<br>PGEM TR: GTC ATA GCT GTT TCC TG	PCR Buffer 1x (Tris-HCl100mM pH 8,5/ 500mM KCl), 2,5mM MgCl, 0,20mM dNTP, 0,30mM of each primer, 1U DNA Polimerase	96°C- 5min (1x); 94°C-30s, 55°C - 40s, 72°C-2min (37x); 72°C- 10min; 4°C - ∞
**Verification of 30pb deletion in LMP1**<br>LMP1.A (S): CGG AAG AGG TGG AAA ACA AA<br>LMP1.S (A): GTG GGG GTC GTC ATC ATC TC	PCR Buffer 1x (Tris-HCl100mM pH 8,5/ 500mM KCl), 2,5mM MgCl, 0,20mM dNTP, 0,30mM of each primer, 1U DNA Polimerase	96°C- 5min (1x); 94°C-30s, 57°C - 40s, 72°C-40s (30x); 72°C- 10min; 4°C - ∞

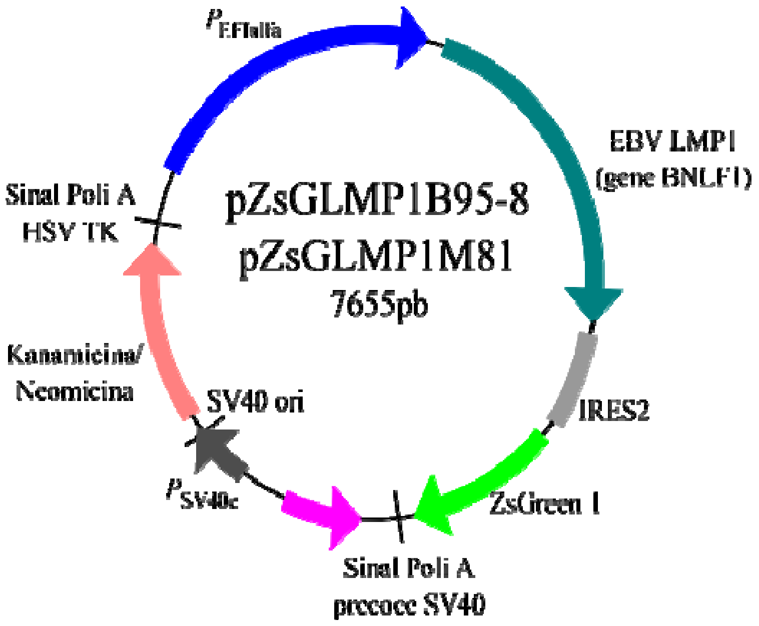

Supplementary Figure S1 - Comercial vector pZsGreen after the LMP1 gene insertion (dark green). The vector was assembled through conventional cloning to access differences between LMP1 variants from EBV in selected miRNAs expression after transient transfection in immortalized nasopharyngeal cells (NP69).

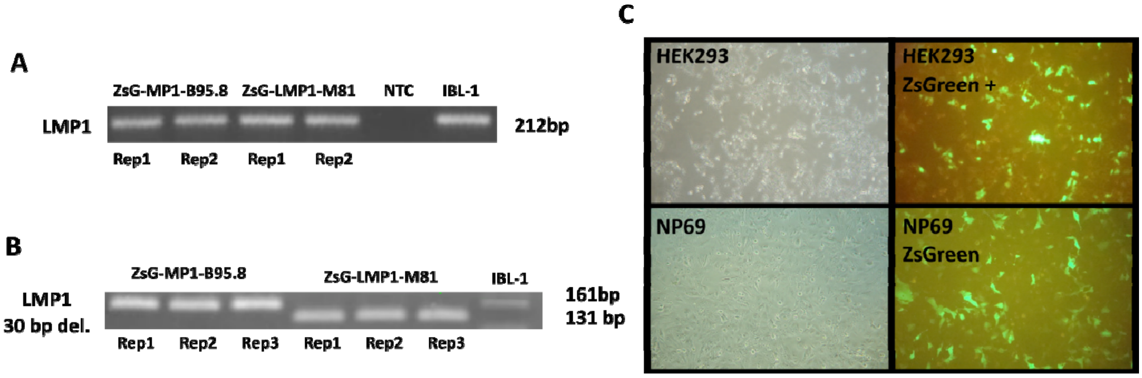

Supplementary Figure S2 - Analysis of LMP1 expression and GFP positivity in HEK293 or NP69 cells after 48h of transfection with the assembled constructs pZsG-LMP1-B95.8 or pZsG-LMP1-M81. PCR amplification of cDNA from (A) HEK293 cells expressing LMP1 from the construct pZsG-LMP1-B95.8 and pZsG-LMP1-M81 and (B) transfected NP69 cells, indicating the 30bp deletion present in LMP1 variant from M81 strain. expressing LMP1 from the construct pZsG-LMP1-B95.8 and pZsG-LMP1-M81. (C) Example of transfection rates in HEK293 and NP69 cells transfected with pZsGreen vector.

Supplementary Table S2 - MiRNAs related to NPC, LMP1, EBV, or cancer in general, selected from literature separated by its probable function as tumor suppressor, oncomiR or dual function. Those miRNAs were used in the qPCR miRNA array to analyze their expression in NP69 cells transfected with LMP1 from two distinct EBV strains, B95.8A or M81.

N°	Gene	Código NCBI	Ref.
miRNAs with tumor supressor properties			
1	hsa-miR-9-5p	MIMAT0000441	[1,2,2]
2	hsa-miR-15a-5p	MIMAT0000068	[3–5]
3	hsa-miR-16-5p	MIMAT0000069	[3,6,7,7]
4	hsa-miR-24-3p	MIMAT0000080	[8–11]
5	hsa-miR-26b-5p	MIMAT0000083	[12–14]
6	hsa-miR-29c-3p	MIMAT0000681	[12,15,16]
7	hsa-miR-30a-3p	MIMAT0000088	[17–19]
8	hsa-miR-34a-5p	MIMAT0000255	[20–22]
9	hsa-miR-34b-5p	MIMAT0000685	[12]
10	hsa-miR-34c-5p	MIMAT0000686	[12,23]
11	hsa-miR-100-5p	MIMAT0000098	[12,24–26]
12	hsa-miR-124-1-5p	MIMAT0004591	[27]
13	hsa-miR-125b-5p	MIMAT0000423	[12,28,28,29]
14	hsa-miR-135b-5p	MIMAT0000758	[30,31]
15	hsa-miR-138-5p	MIMAT0000430	[30,32,33]
16	hsa-miR-140-3p	MIMAT0004597	[12,34]
17	hsa-miR-140-5p	MIMAT0000431	[35–38]
18	hsa-miR-143-3p	MIMAT0000435	[12,39]
19	hsa-miR-145-5p	MIMAT0000437	[12,40,41]
20	hsa-miR-148a-3p	MIMAT0000243	[42]
21	hsa-miR-152-3p	MIMAT0000438	[43,44]
22	hsa-miR-188	MIMAT0000457	[45–47]
23	hsa-miR-199a-3p	MIMAT0000232	[12,48,49]
24	hsa-miR-200a-3p	MIMAT0004613	[50–57]
25	hsa-miR-200b-3p	MIMAT0000318	[53,56,58,59]
26	hsa-miR-200c-3p	MIMAT0000617	[60–62]
27	hsa-miR-141-3p	MIMAT0000432	[63–65]
28	hsa-miR-429	MIMAT0001536	[4,66,67]
29	hsa-miR-204-5p	MIMAT0000265	[68–70]
30	hsa-miR-223-3p	MIMAT0000280	[16,16,71]
31	hsa-miR-218-5p	MIMAT0000275	[72]
32	hsa-miR-320[a]	MIMAT0000510	[73]
33	hsa-miR-338-3p	MIMAT0000763	[74]
34	hsa-miR-363-3p	MIMAT0000707	[12,75–77]
35	hsa-miR-451a	MIMAT0001631	[12,78–80]
36	hsa-miR-494-3p	MIMAT0002816	[81–83]
37	hsa-miR-497-5p	MIMAT0002820	[84]
38	hsa-miR-506-5p	MIMAT0022701	[85]
39	hsa-miR-539-3p	MIMAT0022705	[86–88]
40	hsa-miR-574-3p	MIMAT0003239	[89,90]
41	hsa-miR-634	MIMAT0003304	[91]
42	hsa-let-7c-5p	MIMAT0000064	[92–94]
43	hsa-let-7d-5p	MIMAT0000064	[12,95,96]
44	hsa-let-7i-5p	MIMAT0000415	[97]
miRNAs with oncomir properties			
45	hsa-miR-10b-5p	MIMAT0000254	[98]
46	hsa-miR-17-5p	MIMAT0000070	[16,99,100]
47	hsa-miR-17-3p	MIMAT0000071	[99]
48	hsa-miR-18a-5p	MIMAT0000072	[101,102]
49	hsa-miR-19a-3p	MIMAT0000073	[103,104]
50	hsa-miR-20[a]	MIMAT0000075	[16,100]
51	hsa-miR-92a-3p	MIMAT0000092	[105]
52	hsa-miR-18b-5p	MIMAT0001412	[106–108]
53	hsa-miR-21-5p	MIMAT0000076	[109]
54	hsa-miR-23a-3p	MIMAT0000078	[110,111]
55	hsa-miR-23b-3p	MIMAT0000418	[112,113]
56	hsa-miR-27a-3p	MIMAT0000084	[114–116]
57	hsa-miR-29a-3p	MIMAT0000086	[117]
58	hsa-miR-31-3p	MIMAT0004504	[118]
59	hsa-miR-93-5p	MIMAT0000093	[31,119,120]
60	hsa-miR-142-3p	MIMAT0000434	[121]
61	hsa-miR-146a-5p	MIMAT0000449	[122–124]
62	hsa-miR-149-3p	MIMAT0004609	[125]
63	hsa-miR-151a-5p	MIMAT0004697	[126,127]
64	hsa-miR-155-5p[a]	MIMAT0000646	[128–130]
65	hsa-miR-181b-5p	MIMAT0000257	[131–133]
66	hsa-miR-183-5p	MIMAT0000261	[30,31,134,135]
67	hsa-miR-185-5p	MIMAT0000455	[136,137]
68	hsa-miR-191-5p	MIMAT0000440	[12,138]
69	hsa-miR-192-5p	MIMAT0000222	[139,140]
70	hsa-miR-205-5p	MIMAT0000266	[31,50,141]
71	hsa-miR-210-3p	MIMAT0000267	[142,143]
72	hsa-miR-214-3p	MIMAT0000271	[144–147]
73	hsa-miR-221-3p	MIMAT0000278	[148,149]
74	hsa-miR-222-3p	MIMAT0000279	[148–150]
75	hsa-miR-224-5p	MIMAT0000281	[151,152]
76	hsa-miR-373-3p	MIMAT0000726	[153]
77	hsa-miR-374a-5p	MIMAT0000727	[154–157]
78	hsa-miR-421	MIMAT0003339	[12,158]
79	hsa-miR-663b	MIMAT0005867	[159]
80	hsa-miR-744-5p	MIMAT0004945	[160–162]
81	hsa-miR-4792	MIMAT0019964	[163]
82	hsa-let-7e-5p	MIMAT0000066	[164,165]
miRNAs with tumor supressor and oncomir properties			
83	hsa-miR-10a-5p	MIMAT0000253	[166,167]
84	hsa-miR-26a-5p	MIMAT0000082	[12,168–170]
85	hsa-miR-132-3p	MIMAT0000426	[171–173]
86	hsa-miR-144-3p	MIMAT0000436	[174–176]
87	hsa-miR-181a-5p	MIMAT0000256	[177–180]

N°	Gene	Código NCBI	Ref.
88	hsa-miR-184	MIMAT0000454	[181–184]
89	hsa-miR-203a-3p	MIMAT0000264	[12,185,186]
90	hsa-miR-372	MIMAT0000724	[187–189]
91	hsa-miR-378a-3p	MIMAT0000732	[190–192]
92	PCR positive control (PPC)		
93	Reverse transcription miRNA control (miRTC)		
Endogenous RNAs (normalization purposes)			
94	U6	26827	[193]
95	RNU-44	26806	[193]
96	RNU-48	26801	[194]

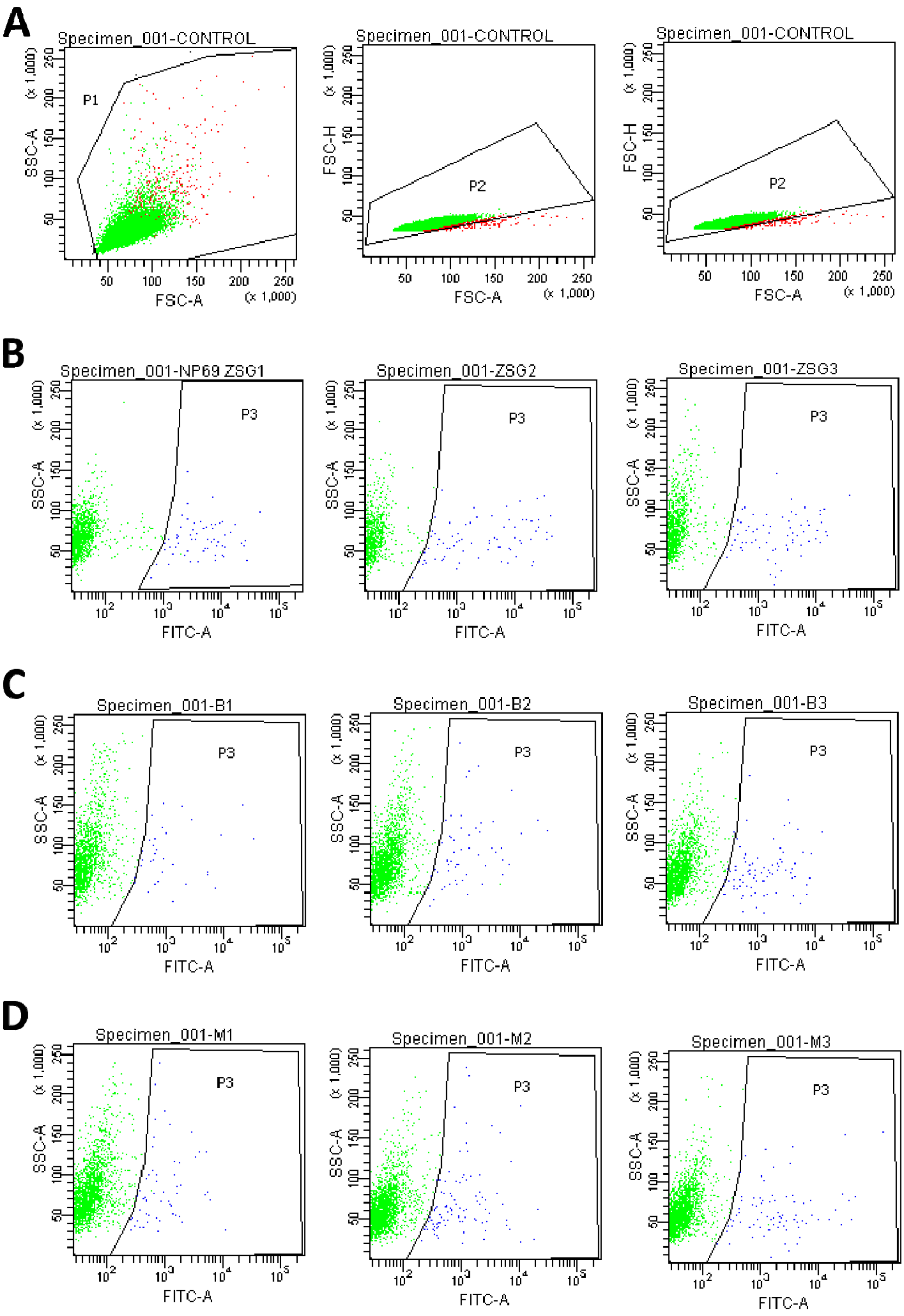

Supplementary Figure S3 - FACS of NP69^{SV40} cells after 48h of transient transfection with pZsGreen (control), pZsG-LMP1-B95.8 or pZsG-LMP1-M81 vectors.

Supplementary Table S3 – Results (Cell count and percentage) from FACS of NP69^{SV40} cells after 48h of transient transfection with pZsGreen (control), pZsG-LMP1-B95.8 or pZsG-LMP1-M81 vectors.

Sample	Populations	# colected cells	# Events	% Parent	% Total
Control (GFP negative)	All events	-	10,000	-	100
	P1	-	9,923	99.2	99.2
	P2	-	9,037	91.1	90.4
	P3	0	0	0	0
NP69 ZsG (Empty Vector)					
NP69-ZsG-Rep1	All events	-	10,000	-	100
	P1	-	9,934	99.3	99.3
	P2	-	7,964	80.2	79.6
	P3	16,000	191	2.4	1.9
NP69-ZsG-Rep2	All events	-	10,000	-	100
	P1	-	9,956	99.6	99.6
	P2	-	8,726	87.6	87.3
	P3	23,851	212	2.4	2.1
NP69-ZsG-Rep3	All events	-	10,000	-	100
	P1	-	9,927	99.3	99.3
	P2	-	8,940	90.1	89.4
	P3	23,700	225	2.5	2.2
NP69 ZsG-LMP1B95.8					
NP69 ZsG-LMP1B95.8 Rep1	All events	-	10,000	-	100
	P1	-	9,813	98.1	98.1
	P2	-	9,039	92.1	90.4
	P3	17,000	109	1.2	1.1
NP69 ZsG-LMP1B95.8 Rep2	All events	-	10,000	-	100
	P1	-	9,836	98.4	98.4
	P2	-	8,542	86.8	85.4
	P3	30,000	177	2.1	1.8
NP69 ZsG-LMP1B95.8 Rep3	All events	-	10,000	-	100
	P1	-	9,908	99.1	99.1
	P2	-	8,795	88.8	87.9
	P3	33,700	244	2.8	2.4
NP69 ZsG-LMP1M81		-			
NP69 ZsG-LMP1M81 Rep1	All events	-	10,000	-	100
	P1	-	9,827	98.3	98.3
	P2		8,340	84.9	83.4
	P3	33,163	175	2.1	1.8
	All events	-	10,000	-	100
	P1	-	9,918	99.2	99.2
	P2	-	9,646	97.3	96.5
	P3	41,220	313	3.2	3.1
	All events	-	10,000	-	100
	P1	-	9,941	99.4	99.4
	P2	-	8,660	87.1	86.6
	P3	33,130	294	3.4	2.9

Supplementary Table S4 - Raw data (CT) after qPCR array using cDNA from NP69^{SV40} cells transfected with pZsGreen (control), pZsG-LMP1-B95.8 or pZsG-LMP1-M81 vectors and sorted by FACS.

	pZsG Rep1	pZsG Rep2	pZsG Rep3	pZsG-LMP1B95.8 Rep1	pZsG-LMP1B95.8 Rep2	pZsG-LMP1B95.8 Rep3	pZsG-LMP1M81 Rep1	pZsG-LMP1M81 Rep2	pZsG-LMP1M81 Rep3
**hsa-miR-9-5p**	33.19	32.2	32.37	31.27	31.65	32.03	31.57	33.71	32.48
**hsa-miR-15a-5p**	30.91	31.12	31.47	31.25	31.23	31.44	30.51	31.29	31.57
**hsa-miR-16-5p**	27.05	27.87	26.89	26.47	26.52	26.55	26.17	27.13	27.06
**hsa-miR-24-3p**	28.38	29.18	27.45	27.1	27.18	27.54	26.99	28.36	27.7
**hsa-miR-26b-5p**	30.12	30.38	30.11	28.19	29.87	30.08	29.27	31.19	29.79
**hsa-miR-29c-3p**	30.97	32.86	31.04	29.98	30.98	30.45	30.66	31.85	31.76
**hsa-miR-30a-3p**	29.45	29.77	28.57	28.01	28.31	29.5	28.52	29.62	29.17
**hsa-miR-34a-5p**	32.74	34.35	32.31	31.04	32.29	30.83	31.53	31.55	31.84
**hsa-miR-34b-5p**	0.00	0.00	0.00	0.00	0.00	0.00	0.00	0.00	0.00
**hsa-miR-34c-5p**	34.23	0.00	0.00	0.00	0.00	0.00	34.32	0.00	0.00
**hsa-miR-100-5p**	30.98	31.33	31.6	30.84	31.38	31.21	30.69	31.48	30.56
**hsa-miR-124-5p**	37.62	35.97	0.00	37.02	36.69	37.75	0.00	37.22	36.9
**hsa-miR-125b-5p**	31.08	31.3	31.52	30.19	30.98	31.15	31.55	31.56	31.48
**hsa-miR-135b-5p**	0.00	0.00	0.00	0.00	0.00	0.00	0.00	36.32	0.00
**hsa-miR-138-5p**	0.00	0.00	0.00	0.00	0.00	32.68	0.00	0.00	0.00
**hsa-miR-140-3p**	0.00	0.00	33.43	32.89	34.74	0.00	34.4	0.00	33.19
**hsa-miR-140-5p**	0.00	35.95	0.00	32.06	34.92	0.00	36.67	0.00	0.00
**hsa-miR-143-3p**	0.00	0.00	35.58	0.00	34.52	0.00	34.2	0.00	0.00
**hsa-miR-145-5p**	0.00	0.00	0.00	0.00	0.00	38.52	37.53	0.00	0.00
**hsa-miR-148a-3p**	33.14	0.00	0.00	34.33	33.97	39.62	33.53	0.00	34.1
**hsa-miR-152-3p**	32.28	32.89	32.21	31.13	31.3	31.5	31.77	32.45	32.19
**hsa-miR-188-5p**	33.34	35.26	35.06	33.19	33.2	32.95	32.71	34.22	33.25
**hsa-miR-199a-3p**	34.31	0.00	35.53	34.39	0.00	0.00	0.00	34.33	0.00
**hsa-miR-188-3p**	31.16	30.56	30.35	29.03	29.62	29.63	29.73	30.08	29.58
**hsa-miR-200b-3p**	0.00	34.6	40	35.93	33.86	0.00	34.47	33.93	33.48
**hsa-miR-200c-3p**	34.86	37.34	0.00	33.43	35.84	32.74	32.66	34.68	34.29
**hsa-miR-141-3p**	34.85	0.00	0.00	0.00	36.66	0.00	0.00	0.00	0.00
**hsa-miR-429**	0.00	34.93	0.00	37.35	36.51	36.55	37.57	36.02	35.92
**hsa-miR-204-5p**	0.00	39.85	37.26	0.00	35.07	0.00	37.64	0.00	0.00
**hsa-miR-223-3p**	34.73	0.00	32.7	34.94	0.00	34.16	32.57	0.00	35.84
**hsa-miR-218-5p**	0.00	0.00	0.00	0.00	0.00	35.76	34.29	0.00	34.54
**hsa-miR-320[a]**	27.36	28.37	27.28	26.83	27.14	27.55	26.64	27.94	27.74
**hsa-miR-338-3p**	0.00	0.00	0.00	0.00	0.00	0.00	0.00	0.00	0.00
**hsa-miR-363-3p**	0.00	0.00	0.00	0.00	0.00	0.00	38.85	0.00	0.00
**hsa-miR-451[a]**	0.00	35.46	0.00	34.01	33.46	32.67	33.79	0.00	0.00
**hsa-miR-494-3p**	31.13	31.39	30.22	29.26	30.22	29.89	29.67	30.45	29.46
**hsa-miR-497-5p**	0.00	0.00	37.17	0.00	0.00	34.18	35.21	0.00	0.00
**hsa-miR-506-5p**	0.00	0.00	0.00	0.00	0.00	0.00	0.00	0.00	0.00
**hsa-miR-539-3p**	0.00	0.00	0.00	0.00	0.00	0.00	0.00	0.00	0.00
**hsa-miR-574-3p**	32.08	31.58	31.05	30.44	30.6	31.43	30.56	30.75	30.01

	pZsG Rep1	pZsG Rep2	pZsG Rep3	pZsG-LMP1B95.8 Rep1	pZsG-LMP1B95.8 Rep2	pZsG-LMP1B95.8 Rep3	pZsG-LMP1M81 Rep1	pZsG-LMP1M81 Rep2	pZsG-LMP1M81 Rep3
**hsa-miR-634**	0.00	0.00	0.00	0.00	0.00	0.00	0.00	0.00	0.00
**hsa-let-7c-5p**	29.67	30.29	29.84	28.6	29.99	29.56	29.25	30.12	29.81
**hsa-let-7c-5p**	29.6	30.33	29.77	28.62	29.52	29.39	29.31	30.39	29.7
**hsa-let-7i-5p**	29.83	30.87	29.09	28.09	28.87	28.76	28.56	29.3	28.86
**hsa-miR-10b-5p**	0.00	0.00	0.00	34.3	35.85	0.00	0.00	33.3	0.00
**hsa-miR-17-5p**	29.55	30.12	29.11	29.03	29.48	28.96	29.03	29.9	29.06
**hsa-miR-17-3p**	36.51	36.24	0.00	0.00	34.79	0.00	33.83	0.00	33.61
**hsa-miR-18a-5p**	30.97	32.5	31.68	31.89	32.93	31.75	30.79	33.31	32.34
**hsa-miR-19a-3p**	31.6	35.28	31.79	30.67	31.79	30.52	30.24	31.55	33.07
**hsa-miR-20a-5p**	28.67	29.62	28.52	28.08	28.59	28.49	27.91	29.12	29.07
**hsa-miR-92a-3p**	27.27	27.83	26.49	25.9	26.5	26.41	26.66	27.47	26.79
**hsa-miR-18b-5p**	31.83	30.94	30.84	28.83	30.16	29.93	31.03	30.27	29.29
**hsa-miR-21-5p**	24.41	24.96	23.36	22.04	22.81	23.51	22.83	24.03	23.43
**hsa-miR-23a-3p**	26.28	27.04	25.76	25.03	25.36	25.39	25.07	26.32	25.78
**hsa-miR-23b-3p**	27.81	28.36	27.44	27.38	27.37	27.27	27.12	28.53	28.04
**hsa-miR-27a-3p**	30.24	31.01	29.76	28.49	28.79	28.64	28.7	29.96	29.41
**hsa-miR-29a-3p**	30.96	31.55	30.96	29.37	30.9	29.85	30.15	30.67	30.54
**hsa-miR-31-3p**	33.15	0.00	34.38	0.00	34.08	34.42	37.4	0.00	34.59
**hsa-miR-93-5p**	29.86	30.42	29.65	28.67	29.35	29.47	29.23	29.97	29.79
**hsa-miR-142-3p**	35.88	0.00	39.94	0.00	0.00	34.7	34.47	0.00	0.00
**hsa-miR-146a-5p**	0.00	0.00	0.00	35.06	0.00	0.00	0.00	0.00	0.00
**hsa-miR-149-3p**	29.62	28.67	28.19	28.22	28.27	29.26	28.99	28.83	27.92
**hsa-miR-151a-5p**	29.18	30.22	28.62	28.29	28.26	28.98	28.23	29.5	28.95
**hsa-miR-155-5p**	34.54	36.6	34.19	31.48	31.88	31.89	31.66	32.36	33.05
**hsa-miR-181b-5p**	29.12	30.05	28.93	28.44	28.76	28.73	28.77	29.83	29.41
**hsa-miR-183-5p**	37.52	33.88	34.13	33.43	33.35	32.62	34.6	0.00	33.72
**hsa-miR-185-5p**	37.99	39.9	34.11	33.1	33.36	32.6	31.43	33.74	33.87
**hsa-miR-191-5p**	29.98	29.76	29.86	28.4	28.33	29.02	28.54	29.92	29.56
**hsa-miR-192-5p**	37.68	0.00	33.11	33.33	33.71	0.00	33.5	0.00	32.53
**hsa-miR-205-5p**	38.78	37.77	33.77	33.36	36.45	33.69	35.69	34.04	35.4
**hsa-miR-210-3p**	31.96	32.38	31.1	30.17	29.82	30.85	30.28	30.49	31.33
**hsa-miR-214-3p**	0.00	0.00	0.00	35.18	0.00	35.78	32.18	0.00	33.18
**hsa-miR-221-3p**	26.79	27.37	25.9	25.64	26.24	26.28	26.48	27.32	26.55
**hsa-miR-222-3p**	27.49	27.78	26.56	26.13	26.67	27.06	26.69	27.76	26.6
**hsa-miR-224-5p**	29.7	30.39	29.1	28.9	29.04	29.57	29.42	30.35	29.14
**hsa-miR-373-3p**	0.00	0.00	0.00	0.00	0.00	0.00	0.00	0.00	0.00
**hsa-miR-374a-5p**	35.49	0.00	0.00	33.89	35.16	33.87	34.57	0.00	0.00
**hsa-miR-421**	31.22	31.95	30.29	29.9	30.83	32.4	29.54	31.07	30.04
**hsa-miR-663b**	34.85	35.72	33.79	34.9	33.3	35.64	33.53	0.00	0.00
**hsa-miR-744-5p**	29.51	29.67	29.89	29.98	28.94	29.21	29.67	30.42	29.51
**hsa-miR-4792**	30.44	30.59	28.41	28.94	29.32	30.12	30	29.64	29.23
**hsa-let-7e-5p**	27.91	28.22	27.59	26.9	28.12	27.33	27.36	28.55	28.16
**hsa-miR-10a-5p**	0.00	0.00	0.00	34.2	34.18	32.99	0.00	0.00	34.49
**hsa-miR-26a-5p**	29.9	30.72	28.28	27.41	28.47	28.81	27.83	29.89	28.7

	pZsG Rep1	pZsG Rep2	pZsG Rep3	pZsG-LMP1B95.8 Rep1	pZsG-LMP1B95.8 Rep2	pZsG-LMP1B95.8 Rep3	pZsG-LMP1M81 Rep1	pZsG-LMP1M81 Rep2	pZsG-LMP1M81 Rep3
**hsa-miR-132-3p**	33.05	34.3	32.23	31.61	34.03	33.09	32.97	34.16	33.31
**hsa-miR-144-3p**	0.00	0.00	0.00	0.00	0.00	0.00	0.00	0.00	0.00
**hsa-miR-181a-5p**	30.94	31.97	30.65	29.64	29.79	30.18	30.49	30.18	30.82
**hsa-miR-184**	0.00	0.00	0.00	0.00	0.00	39.01	0.00	0.00	0.00
**hsa-miR-203a-3p**	0.00	37.16	32.54	32.77	35.28	31.77	32.82	0.00	32.19
**hsa-miR-372-3p**	0.00	0.00	0.00	0.00	0.00	0.00	0.00	0.00	0.00
**hsa-miR-378a-3p**	31.09	31.87	31.32	31.09	30.76	30.63	31.05	33.76	31.2
**RNU6-6P**	28.43	28.01	26.79	25.47	25.22	25.91	26.47	26.12	25.92
**SNORD68**	25.95	25.63	24.37	22.94	24.7	24.19	25.2	25.52	23.62
**SNORD72**	28.65	28.95	28.52	28.26	27.87	27.66	28.17	28.58	28
**miRTC**	32.15	31.24	29.9	27.83	29.87	29.97	30.34	30.41	29.12
**PPC**	18.16	17.9	17.9	18.29	17.75	17.97	17.93	17.87	17.97

Supplementary Table S5 – Results of RT-qPCR arrays analysis and verification of up or downregulated miRNAs of NP69^{SV40} cells transfected with pZsGreen (control), pZsG-LMP1-B95.8 or pZsG-LMP1-M81 vectors and sorted by FACS.

miRNA	pZsG-LMP1B95.8		pZsG-LMP1M81		pZsG-LMP1M81 x B95,8	
	p-value	Fold Reg	p-value	Fold Reg	p-value	Fold Reg
hsa-miR-9-5p	0.203	-1.49	0.395	-1.60	0.901	-1.07
hsa-miR-15a-5p	0.074	-3.02	0.290	-1.76	0.213	1.72
hsa-miR-16-5p	0.166	-1.62	0.631	-1.29	0.436	1.25
hsa-miR-24-3p	0.243	-1.31	0.862	-1.15	0.586	1.14
hsa-miR-26b-5p	0.396	-1.55	0.363	-1.67	0.933	-1.08
hsa-miR-29c-3p	0.507	-1.23	0.456	-1.58	0.755	-1.28
hsa-miR-30a-3p	0.079	-1.74	0.210	-1.62	0.822	1.07
hsa-miR-34a-5p	0.883	-1.11	0.563	1.14	0.560	1.27
hsa-miR-34b-5p	0.037	-2.74	0.118	-1.81	0.120	1.51
hsa-miR-34c-5p	0.037	-2.74	0.118	-1.81	0.120	1.51
hsa-miR-100-5p	0.104	-2.45	0.352	-1.38	0.049	1.78
hsa-miR-124-5p	0.037	-2.74	0.118	-1.81	0.120	1.51
hsa-miR-125b-5p	0.142	-1.90	0.120	-2.12	0.658	-1.12
hsa-miR-135b-5p	0.037	-2.74	0.118	-1.81	0.120	1.51
hsa-miR-138-5p	0.046	-2.54	0.118	-1.81	0.203	1.41
hsa-miR-140-3p	0.038	-2.67	0.118	-1.81	0.126	1.48
hsa-miR-140-5p	0.062	-2.20	0.118	-1.81	0.405	1.22
hsa-miR-143-3p	0.037	-2.74	0.118	-1.81	0.120	1.51
hsa-miR-145-5p	0.037	-2.74	0.118	-1.81	0.120	1.51
hsa-miR-148a-3p	0.037	-2.74	0.118	-1.81	0.120	1.51
hsa-miR-152-3p	0.381	-1.23	0.335	-1.45	0.661	-1.17
hsa-miR-188-5p	0.038	-2.71	0.162	-1.69	0.126	1.60
hsa-miR-199a-3p	0.037	-2.74	0.118	-1.81	0.120	1.51
hsa-miR-188-3p	0.432	-1.14	0.932	1.03	0.291	1.17
hsa-miR-200b-3p	0.037	-2.74	0.118	-1.81	0.120	1.51
hsa-miR-200c-3p	0.044	-2.58	0.173	-1.67	0.166	1.54
hsa-miR-141-3p	0.037	-2.74	0.118	-1.81	0.120	1.51
hsa-miR-429	0.037	-2.74	0.118	-1.81	0.120	1.51
hsa-miR-204-5p	0.037	-2.74	0.118	-1.81	0.120	1.51
hsa-miR-223-3p	0.014	-2.93	0.114	-1.76	0.129	1.67
hsa-miR-218-5p	0.037	-2.74	0.118	-1.81	0.120	1.51
hsa-miR-320a	0.138	-1.94	0.477	-1.54	0.473	1.26
hsa-miR-338-3p	0.037	-2.74	0.118	-1.81	0.120	1.51
hsa-miR-363-3p	0.037	-2.74	0.118	-1.81	0.120	1.51
hsa-miR-451a	0.046	-2.54	0.118	-1.81	0.207	1.40
hsa-miR-494-3p	0.182	-1.26	0.441	1.15	0.139	1.44
hsa-miR-497-5p	0.037	-2.74	0.118	-1.81	0.120	1.51
hsa-miR-506-5p	0.037	-2.74	0.118	-1.81	0.120	1.51

miRNA	pZsG-LMP1B95.8		pZsG-LMP1M81		pZsG-LMP1M81 x B95,8	
	p-value	Fold Reg	p-value	Fold Reg	p-value	Fold Reg
hsa-miR-539-3p	0.037	-2.74	0.118	-1.81	0.120	1.51
hsa-miR-574-3p	0.055	-1.63	0.141	1.21	0.007	1.97
hsa-miR-634	0.037	-2.74	0.118	-1.81	0.120	1.51
hsa-let-7c-5p	0.167	-1.87	0.313	-1.57	0.588	1.19
hsa-let-7c-5p	0.200	-1.66	0.262	-1.69	0.913	-1.02
hsa-let-7i-5p	0.667	-1.07	0.747	1.12	0.430	1.20
hsa-miR-10b-5p	0.037	-2.74	0.118	-1.81	0.120	1.51
hsa-miR-17-5p	0.047	-2.02	0.194	-1.51	0.264	1.34
hsa-miR-17-3p	0.037	-2.74	0.118	-1.81	0.120	1.51
hsa-miR-18a-5p	0.159	-3.80	0.377	-2.27	0.398	1.68
hsa-miR-19a-3p	0.582	-1.25	0.917	-1.25	0.621	-1.00
hsa-miR-20a-5p	0.128	-1.87	0.481	-1.54	0.486	1.22
hsa-miR-92a-3p	0.090	-1.44	0.145	-1.55	0.855	-1.08
hsa-miR-18b-5p	0.796	1.08	0.709	1.11	0.922	1.03
hsa-miR-21-5p	0.917	1.00	0.922	-1.03	0.984	-1.03
hsa-miR-23a-3p	0.269	-1.28	0.828	-1.16	0.621	1.10
hsa-miR-23b-3p	0.059	-1.90	0.236	-1.84	0.736	1.03
hsa-miR-27a-3p	0.516	1.18	0.725	1.09	0.964	-1.09
hsa-miR-29a-3p	0.525	-1.26	0.758	-1.11	0.714	1.14
hsa-miR-31-3p	0.037	-2.74	0.118	-1.81	0.120	1.51
hsa-miR-93-5p	0.146	-1.56	0.341	-1.46	0.676	1.07
hsa-miR-142-3p	0.037	-2.74	0.118	-1.81	0.120	1.51
hsa-miR-146a-5p	0.037	-2.74	0.118	-1.81	0.120	1.51
hsa-miR-149-3p	0.038	-2.31	0.103	-1.53	0.094	1.52
hsa-miR-151a-5p	0.207	-1.54	0.578	-1.33	0.592	1.16
hsa-miR-155-5p	0.448	-1.15	0.954	-1.15	0.679	1.01
hsa-miR-181b-5p	0.161	-1.66	0.239	-1.77	0.955	-1.07
hsa-miR-183-5p	0.048	-2.51	0.118	-1.81	0.229	1.39
hsa-miR-185-5p	0.049	-2.50	0.909	-1.26	0.281	1.98
hsa-miR-191-5p	0.595	-1.13	0.755	-1.26	0.983	-1.12
hsa-miR-192-5p	0.037	-2.74	0.129	-1.62	0.014	1.69
hsa-miR-205-5p	0.037	-2.74	0.118	-1.81	0.120	1.51
hsa-miR-210-3p	0.744	1.06	0.455	1.20	0.614	1.13
hsa-miR-214-3p	0.037	-2.74	0.363	-1.50	0.171	1.83
hsa-miR-221-3p	0.038	-1.77	0.051	-1.93	0.772	-1.10
hsa-miR-222-3p	0.018	-1.74	0.125	-1.51	0.535	1.15
hsa-miR-224-5p	0.060	-1.86	0.106	-1.70	0.626	1.10
hsa-miR-373-3p	0.037	-2.74	0.118	-1.81	0.120	1.51
hsa-miR-374a-5p	0.037	-2.74	0.118	-1.81	0.120	1.51
hsa-miR-421	0.077	-2.54	0.763	1.06	0.174	2.68
hsa-miR-663b	0.037	-2.74	0.118	-1.81	0.120	1.51
hsa-miR-744-5p	0.130	-2.20	0.119	-2.05	0.961	1.08

miRNA	pZsG-LMP1B95.8		pZsG-LMP1M81		pZsG-LMP1M81 x B95,8	
	p-value	Fold Reg	p-value	Fold Reg	p-value	Fold Reg
**hsa-miR-4792**	0.120	-2.14	0.219	-1.59	0.229	1.35
**hsa-let-7e-5p**	0.053	-2.00	0.141	-1.96	0.827	1.02
**hsa-miR-10a-5p**	0.038	-2.73	0.118	-1.81	0.121	1.51
**hsa-miR-26a-5p**	0.846	-1.04	0.884	-1.02	0.783	1.01
**hsa-miR-132-3p**	0.014	-2.37	0.009	-2.15	0.814	1.11
**hsa-miR-144-3p**	0.037	-2.74	0.118	-1.81	0.120	1.51
**hsa-miR-181a-5p**	0.626	-1.10	0.832	-1.12	0.837	-1.02
**hsa-miR-184**	0.037	-2.74	0.118	-1.81	0.120	1.51
**hsa-miR-203a-3p**	0.072	-2.17	0.042	-1.60	0.514	1.36
**hsa-miR-372-3p**	0.037	-2.74	0.118	-1.81	0.120	1.51
**hsa-miR-378a-3p**	0.190	-1.81	0.180	-2.26	0.749	-1.25

Pathway Enrichment Analysis

The differentially expressed miRNAs from each comparison were then subjected to target prediction analysis using the mirDIP [195,196], chosen because this platform uses an integrative score method, avoiding the miRNA-prediction bias (tendency to identify targets belonging to specific biological processes or pathways). The top 1% gene targets indicated by mirDIP (meaning scores above 0.38) as "*Very High*" scores were selected and subjected to pathway enrichment analysis using the ReactomeFIViz [197] plugin in Cytoscape [198], which is curated and updated manually [199].

To categorize genes present in pathways showing p-value ≤ 0.05, we counted all target genes present in each pathway once, avoiding duplicates, always considering its position on the presented hierarchical category (Additional file 1: Figure S3).

Gene hits inside each pathway found after the enrichment analysis (p-value ≤ 0.05) were dubbed "Gene Sets #2". Even considering the variable number of predicted targets in each Gene Set #1, the proportion of genes incorporated into the respective Gene Set #2 was similar among the comparisons performed: 28% for B95.8 vs Ctrl; 30% M81 vs Ctrl, and 20% for M81 vs B95.8 (Supplementary material figure S3). Then, a Gene Set #3 was generated with unique hits obtained from the respective Gene Set #2, comprising about 20% of target genes for B95.8 vs Ctrl, 3% for M81 vs Ctrl, 2.7% for M81 vs B95.8 (Figure S3). The pathway enrichment analysis was performed using gene sets #2, while gene sets #1 and #3 were used to analyze the overall distribution of target genes among comparisons.

The categories were retrieved from Reactome FIViz, so that pathways could be identified based on its hierarchical levels (Figure S4). They were classified as primary (highest level; broader), secondary, or tertiary (lower level; the most specific ones). The calculation of genes percentage in each class was based on the following upper category. For instance, the percentage of genes in tertiary level was based on the secondary and not on total gene count from gene set #2 (Table S6).

Common features due to LMP1 expression were found upon analysis of differences in miRNA-mediated pathway modulation by LMP1 B95.8 or M81. Compared to control, either LMP1 variant B95.8 or M81 modulated 76.2% of all primary categories identified (16 out of 21; Figure 3B and 4A). Primary categories uniquely regulated included, for instance, "Cell-cell communication" for B95.8 vs Ctrl, and

“Organelle biogenesis and maintenance”, “Cell cycle” and “Metabolism of RNA” for M81 vs Ctrl. For the “Signal transduction” category (comprising 26% and 36% of gene hits for B95.8 and M81, respectively, compared to control), 66.7% of secondary categories were shared for both LMP1 variants, including signaling pathways related to tyrosine-kinase receptors (TKR), MAPKs and WNT. On the other hand, five extra secondary categories (belonging to signal transduction) were identified for LMP1 M81: “Death receptor”, “Integrin signaling”, “mTOR signaling”, “Signaling by leptin” and “Signaling by non-receptor of tyrosine kinases” (Figure 3C and 4C). The subcategory “Signaling by Tyrosine Kinase Receptors” showed a high number of hits for both EBV LMP1 variants. Considering the 12 tertiary categories identified for “Signaling by Tyrosine Kinase Receptors”, 7 (58,3%) were commonly modulated, but LMP1 B95.8 was uniquely involved in EGFR signaling, while M81 was implicated in modulation of ERBB2, ERBB4, FGFR, and NTRKs signaling pathways (Figure 3D and 4D).

Figure 5 shows results obtained after pathway enrichment analysis of the predicted gene set targeted by cellular miRNAs deregulated in NP69^{SV40T} expressing EBV LMP1. As shown in Figure 5A, the Top 5 primary categories for LMP1 variant M81 vs B95.8 were “Signal transduction”, “Metabolism”, “Developmental biology”, “Immune system”, and “Cell cycle”. The “Signal transduction” category accounted for 61 (48%) of all 126 hits, mostly (77%) hits on TKR-mediated signaling. For comparisons involving either viral LMP1 variant considered, a set of 10 primary categories was found, including and “Developmental Biology”, “Gene Expression”, and “Immune System” (Figure 5B). Nonetheless, the two primary categories “Cell cycle” and “Organelle biogenesis and maintenance” showed up in both LMP1 M81 vs control and LMP1 M81 vs LMP1 B95.8 comparisons, while “DNA Replication” (genes involved in M/G1 transition) and “Expand All Signal Transduction” (mostly genes involved in WNT signaling) were found only when comparing LMP1 M81 vs B95.8 (Figure 5B).

For “Signal Transduction”, the top 5 secondary categories found were “Signaling by Receptor Tyrosine Kinases”, “Signaling by type-1 IGFR1”, “MAPK family signaling cascades”, “Intracellular Signaling by second messenger” and “Signaling by TGF-β family members” (Figure 5C). Even though “mTOR signaling” had a small number of hits, it was the only “Signal transduction” subcategory only associated to LMP1 M81, when against B95.8 or the control (Figure 5D). Finally, when analyzing results for

the tertiary category "Tyrosine Kinase Receptor" of "Signal transduction", most hits were obtained for signaling pathways featuring NGF, EGFR, and IGF1R (Figure 5E). Interestingly, FGFR was the only pathway deregulated by LMP1 M81 in both M81 vs Ctrl and M81 vs B95.9 comparisons, while B95.8 vs Ctrl had no exclusive deregulated pathways (Figure 5F).

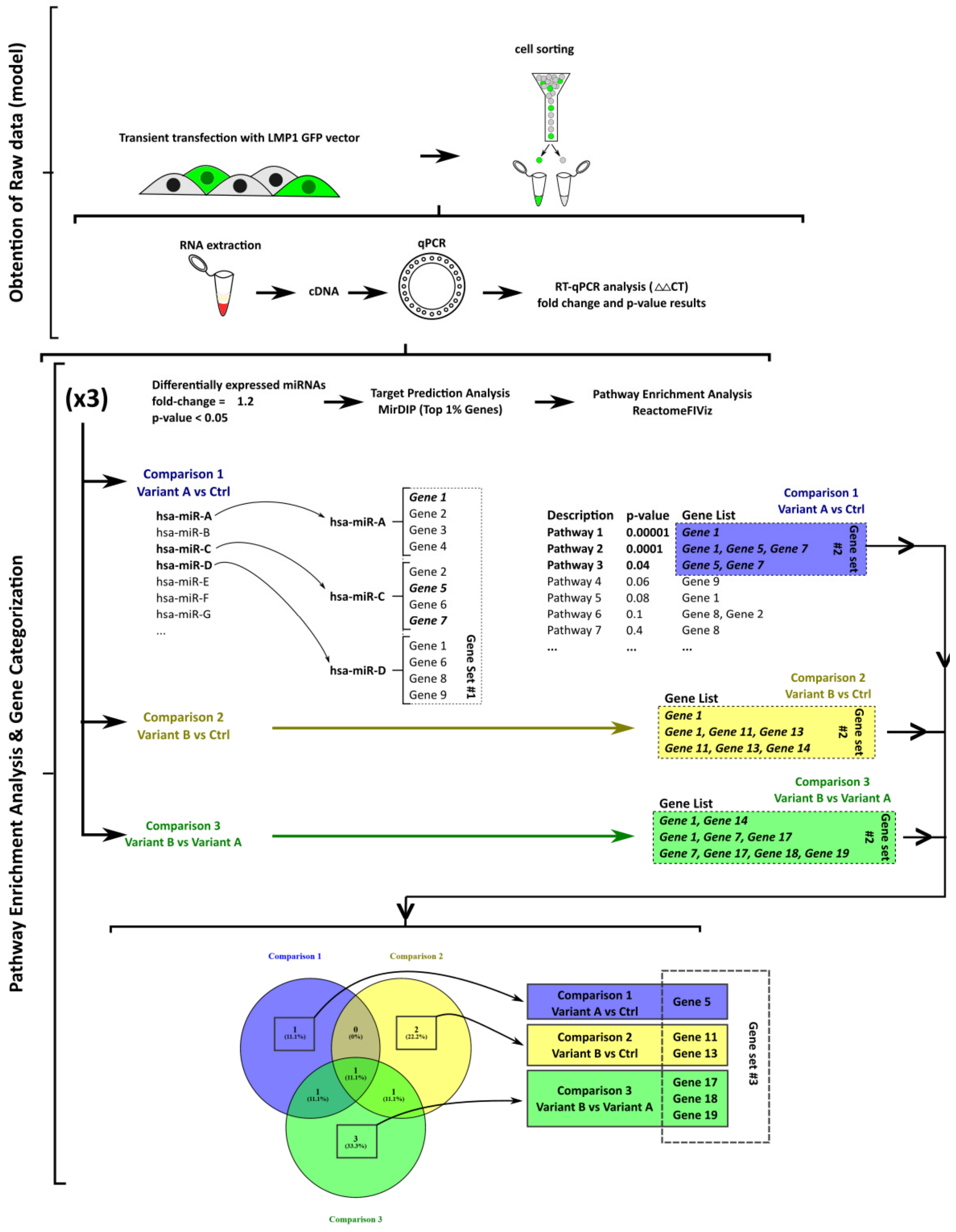

Supplementary Figure S4 – Schematic drawing of steps followed after transfection of NP69^{SV40} cells transfected with pZsGreen (control), pZsG-LMP1-B95.8 or pZsG-LMP1-M81 vectors. and sorted by FACS with pZsGreen vector as control or containing the coding region from LMP1 protein from EBV variants B95.8 or M81. First,48h after transfection, GFP positive cells were sorted by FACs, followed by RNA extraction, cDNA production and RT-qPCR array of 91 pre-selected miRNAs. Then, target prediction

analysis of differentially expressed miRNAs and genes with high scores were selected for Pathway enrichment analysis.

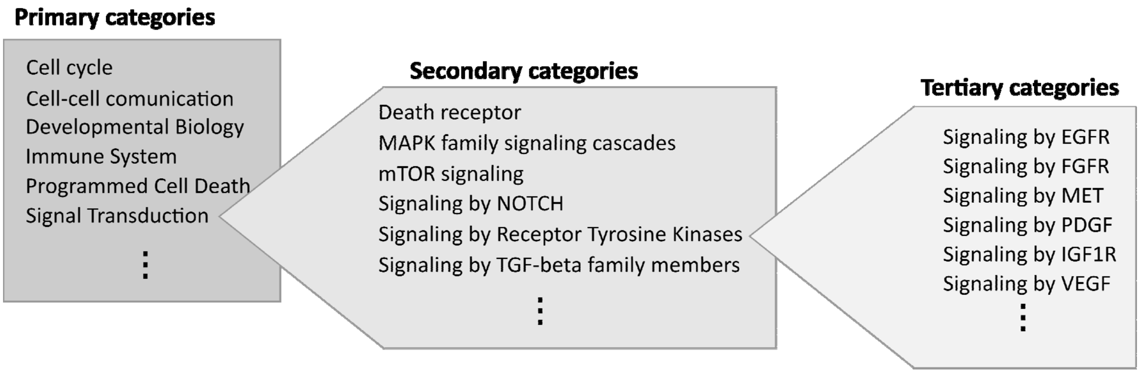

Supplementary Figure S5 - Analysis of target genes from differentially expressed miRNAs in $NP69^{SV40T}$ cells transfected with EBV LMP1 from B95.8 or M81 variants. Example of primary, secondary and tertiary categories organization. Panels C, D and E show results of in silico pathway enrichment analysis of predicted target genes from deregulated miRNAs in $NP69^{SV40}$ cells transfected with LMP1 B95.8, LMP1 M81 or the control vector.

Supplementary Table S6- Analysis of pathway enrichment analysis using predicted genes from gene set #2 targeted by differentially expressed miRNAs from NP69SV40 cells transfected with pZsGreen (control), pZsG-LMP1-B95.8 or pZsG-LMP1-M81 vectors and sorted by FACS. Each group has pathways represented in hits (gene in each pathway) and % hits (percentage of hits compared to next higher category).

	**B95.8 vs Control**	**M81 vs Control**	**M81 vs B95.8**
	**Hits (%)**	**Hits (%)**	**Hits (%)**
Number of genes included in the set:	3100	984	126
**Cell cycle**	-	19 (2)	12 (10)
Cell Cycle Checkpoints	-	- -	6 (50)
Cell Cycle/ Mitotic	-	19 (100)	10 (83)
**Cell-cell comunication**	56 (2)	-	-
Cell junction organization	56 (100)	-	-
**Cellular responses to external stimuli**	96 (3)	40 (4)	-
Cellular responses to stress	96 (100)	40 (100)	-
Cellular Senescence	96 (100)	35 (88)	-
Cellular response to hypoxia	-	5 (13)	-
**Chromatin organization**	148 (5)	51 (5)	-
Chromatin modifying enzymes	148 (100)	51 (5)	-
**Circadian Clock**	38 (1)	18 (2)	-
BMAL1:CLOCK/NPAS2 activates circadian gene expression	38 (100)	16 (89)	-

	B95.8 vs Control<br>Hits (%)	M81 vs Control<br>Hits (%)	M81 vs B95.8<br>Hits (%)
Number of genes included in the set:	3100	984	126
RORA activates gene expression	-	12 (67)	-
**Developmental Biology**	483 (16)	122 (12)	30 (24)
Activation of HOX genes during differentiation	51 (11)	-	-
Axon guidance	376 (78)	122 (100)	26 (87)
Signaling by NODAL	- -	-	4 (13)
Transcriptional regulation of white adipocyte differentiation	63 (13)	-	-
**DNA Replication**	-	-	4 (3)
M/G1 Transition	-	-	4 (100)
**Expand All Signal Transduction**	-	-	3 (2)
Signaling by WNT	-	-	3 (100)
**Extracellular matrix organization**	36 (1)	32 (3)	-
Collagen formation	36 (100)	18 (56)	-
Elastic fibre formation	-	12 (38)	-
Non-integrin membrane-ECM interactions	-	8 (25)	-
**Gene expression (Transcription)**	352 (11)	145 (15)	10 (8)
Epigenetic regulation of gene expression	-	8 (6)	-
PRC2 methylates histones and DNA	-	8 (100)	-
Gene Silencing by RNA	-	21 (14)	-
Post-transcriptional silencing by small RNAs	-	5 (24)	-
RNA Polymerase II Transcription	352 (100)	122 (84)	10 (100)
Generic Transcription Pathway	352 (100)	122 (100)	10 (100)
**Hemostasis**	68 (2)	63 (6)	-
Platelet activation/ signaling and aggregation	21 (31)	54 (86)	-
Platelet homeostasis	53 (78)	8 (13)	-
Cell surface interactions at the vascular wall	-	7 (11)	-
**Immune System**	568 (18)	222 (23)	22 (17)
Adaptive Immune System	141 (25)	61 (27)	16 (73)
Costimulation by the CD28 family	25 (18)	-	-
Rap1 signalling	-	-	4 (25)
Signaling by the B Cell Receptor (BCR)	130 (92)	61 (100)	12 (75)
Cytokine Signaling in Immune system	460 (81)	158 (71)	-
Growth hormone receptor signaling	-	12 (8)	-
Interferon Signaling	-	19 (12)	-
Signaling by Interleukins	331 (72)	120 (76)	-
Innate Immune System	306 (54)	146 (66)	19 (86)
C-type lectin receptors (CLRs)	-	35 (24)	-
DAP12 interactions	225 (74)	80 (55)	19 (100)
Fc epsilon receptor (FCERI) signaling	-	96 (66)	-
Toll Like Receptor 2 (TLR2) Cascade	71 (23)	33 (23)	-
Toll Like Receptor 3 (TLR3) Cascade	74 (24)	30 (21)	-

	B95.8 vs Control Hits (%)	M81 vs Control Hits (%)	M81 vs B95.8 Hits (%)
Number of genes included in the set:	3100	984	126
Toll Like Receptor 4 (TLR4) Cascade	87 (28)	38 (26)	-
Toll Like Receptor 5 (TLR5) Cascade	63 (21)	27 (18)	-
Toll Like Receptor 7/8 (TLR7/8) Cascade	67 (22)	29 (20)	-
Toll Like Receptor 9 (TLR9) Cascade	69 (23)	30 (21)	-
Toll-Like Receptors Cascades	91 (30)	55 (38)	-
**Metabolism**	605 (20)	130 (13)	31 (25)
Inositol phosphate metabolismo	-	5 (4)	-
Integration of energy metabolismo	72 (12)	-	9 (29)
Metabolism of amino acids and derivatives	-	-	6 (19)
Metabolism of carbohydrates	164 (27)	-	-
Metabolism of lipids	391 (65)	128 (98)	16 (52)
The citric acid (TCA) cycle and respiratory electron transport	-	-	3 (10)
**Metabolism of proteins**	553 (18)	174 (18)	7 (6)
Post-translational protein modification	553 (100)	174 (100)	7 (100)
Asparagine N-linked glycosylation	161 (29)	34 (20)	-
Deubiquitination	163 (29)	57 (168)	7 (100)
SUMOylation	85 (15)	- -	-
**Metabolism of RNA**	-	15 (2)	-
Insulin-like Growth Factor-2 mRNA Binding Proteins (IGF2BPs/IMPs/VICKZs) bind RNA	-	2 (13)	-
Processing of Capped Intron-Containing Pre-mRNA	-	13 (87)	-
**Muscle contraction**	87 (3)	-	-
Cardiac conduction	87 (100)	-	-
**Neuronal System**	225 (7)	44 (4)	4 (3)
Potassium Channels	67 (30)	-	-
Protein-protein interactions at synapses	51 (23)	-	-
Transmission across Chemical Synapses	143 (64)	44 (100)	4 (100)
**Organelle biogenesis and maintenance**	-	19 (2)	5 (4)
Mitochondrial biogenesis	-	19 (100)	5 (100)
**Others**	563 (18)	185 (19)	3 (2)
**Programmed Cell Death**	113 (4)	40 (4)	4 (3)
Apoptosis	113 (100)	40 (100)	4 (100)
Apoptotic execution phase	42 (37)	15 (38)	-
Caspase activation via extrinsic apoptotic signalling pathway	-	-	2 (50)
Intrinsic Pathway for Apoptosis	34 (30)	13 (33)	2 (50)
**Signal Transduction**	906 (29)	353 (36)	61 (48)
Death Receptor Signalling	-	16 (5)	-
Integrin signaling	-	9 (3)	-
Intracellular signaling by second messengers	117 (13)	38 (11)	14 (23)
DAG and IP3 signaling	28 (24)	-	5 (36)
PIP3 activates AKT signaling	90 (77)	38 (100)	9 (64)
MAPK family signaling cascades	172 (19)	66 (11)	15 (25)

	B95.8 vs Control Hits (%)	M81 vs Control Hits (%)	M81 vs B95.8 Hits (%)
Number of genes included in the set:	3100	984	126
mTOR signalling	-	13 (4)	4 (7)
Signaling by GPCR	69 (8)	41 (12)	8 (13)
Signaling by Hippo	19 (2)	9 (3)	-
Signaling by Leptin	-	54 (15)	-
Signaling by Non-Receptor Tyrosine Kinases	-	19 (5)	-
Signaling by NOTCH	77 (8)	26 (7)	-
Signaling by Receptor Tyrosine Kinases	474 (52)	188 (53)	47 (77)
Signaling by EGFR	237 (50)	-	20 (43)
Signaling by ERBB2	-	20 (11)	-
Signaling by ERBB4	-	17 (9)	-
Signaling by FGFR	-	24 (13)	13 (28)
Signaling by Insulin receptor	190 (40)	72 (38)	17 (36)
Signaling by MET	44 (9)	24 (13)	-
Signaling by NTRKs	-	62 (33)	-
Signaling by PDGF	241 (51)	91 (48)	-
Signaling by SCF-KIT	208 (44)	80 (43)	17 (36)
Signaling by Type 1 Insulin-like Growth Factor 1 Receptor (IGF1R)	176 (37)	70 (37)	18 (38)
Signaling by VEGF	204 (43)	71 (38)	16 (34)
Signalling by NGF	318 (67)	114 (61)	29 (62)
Signaling by Rho GTPases	239 (26)	81 (23)	-
Signaling by TGF-beta family members	73 (8)	36 (10)	10 (16)
Signaling by BMP	22 (30)	16 (44)	4 (40)
Signaling by TGF-beta Receptor Complex	58 (79)	26 (72)	7 (70)
Signaling by Type 1 Insulin-like Growth Factor 1 Receptor (IGF1R)	175 (19)	70 (20)	18 (30)
Signaling by WNT	171 (19)	66 (19)	3 (5)
Beta-catenin independent WNT signaling	-	37 (56)	-
TCF dependent signaling in response to WNT	116 (68)	41 (62)	3 (100)
**Transport of small molecules**	137 (4)	16 (2)	10 (8)
Aquaporin-mediated transport	0	-	5 (50)
Plasma lipoprotein assembly, remodeling, and clearance	0	-	6 (60)
SLC-mediated transmembrane transport	106 (77)	16 (100)	-
Ion channel transport	34 (25)	-	-
**Vesicle-mediated transport**	391 (13)	120 (12)	-
Membrane Trafficking	391 (100)	120 (100)	-

Supplementary Table S7– Descriptive list of predicted genes found by mirDIP encountered in each primary category after pathway enrichment analysis using differentially expressed miRNAs from NP69SV40 cells transfected with pZsGreen (control), pZsG-LMP1-B95.8 or pZsG-LMP1-M81 vectors and sorted by FACS.

**Primary categories**	**B95.8 vs Ctrl**	**M81 vs Ctrl**	**M81 vs B95.8**
	**(#) Genes**	**(#) Genes**	**(#) Genes**
Cell Cycle		*(19)* *SMC4; SMC2; PRKCB; PRKCA; ARPP19; POM121C; TPR; BANF1; GORASP2; ENSA; RAB1A; CNEP1R1; LPIN1; NUP50; NUP58; NUP43; RB1; POM121; RANBP2*	*(12)* *MCM10; MCM6; PSMD5; CDC25A; RB1; MCM7; TYMS; RAD1; DYRK1A; MCM8; ORC4; CDC7*
Cell-Cell communication	*(56)* *CLDN14; CLDN18; CTNND1; CDH9; PVR; NECTIN3; ARHGEF6; PARD6B; MPP5; NECTIN2; CDH8; NECTIN1; NECTIN4; CDH1; CDH2; CDH5; CADM3; CDH7; CADM1; LAMA3; CDH24; CRB3; FERMT2; RSU1; TESK1; PARVA; AFDN; CDH4; CDH6; CDH11; CDH10; CDH13; CDH12; CDH17; ITGA6; LIMS2; CD151; LIMS1; FBLIM1; LAMB3; PATJ; JUP; PXN; ITGB1; ITGB4; CLDN23; CLDN22; PLEC; LAMC2; VASP; CTNNA1; F11R; ACTN1; PRKCI; PARD6G; FLNC*		
Cellular responses to external stimuli	*(96)* *MOV10; ATM; POT1; MAPK14; TNRC6C; TNRC6B; TNRC6A; IL1A; CBX4; CBX2; EHMT1; EHMT2; STAT3; ANAPC16; ANAPC11; MAP3K5; MAPK3; MAPK1; PHC1; MAPK7; PHC3; PHC2; NFKB1; RELA; MAPK9; NBN; CDKN2B; CDKN2A; SUZ12; CDKN2D; SP1; MAP2K7; KDM6B; HMGA2; HMGA1; LMNB1; E2F3; MDM2; MDM4; MAPK8; HIRA; CXCL8;*	*(40)* *CDKN1A; ATM; HIF1A; MAPK14; TNRC6C; TNRC6B; TNRC6A; IL1A; STAT3; JUN; RB1; MAP3K5; ETS2; MAPK3; ETS1; PHC1; H3F3A; CITED2; MAPK9; ASF1A; SUZ12; SP1; PHC3; HMGA2; BMI1; EP400; VEGFA; POT1; EP300; RPS6KA3; E2F3; HIRA; ARNT; CXCL8; MAPK1; CDK6; MAPK10; AGO1; TFDP2; TFDP1*	

Primary categories	B95.8 vs Ctrl	M81 vs Ctrl	M81 vs B95.8
	(#) Genes	(#) Genes	(#) Genes
	FZR1; RBBP7; RBBP4; TERF2; UBA52; TERF1; CDK6; UBC; MAPK10; RNF2; CDKN1B; CDKN1A; MAP2K4; UBE2D1; MAP2K3; ERF; H1F0; EP400; MAP2K6; CBX6; EED; MAPKAPK2; UBN1; CEBPB; JUN; CDK2; RB1; ETS2; ETS1; H3F3A; ASF1A; FOS; RAD50; UBE2E1; BMI1; MINK1; IGFBP7; SCMH1; EZH2; ANAPC7; RPS6KA1; RPS6KA2; RPS6KA3; TP53; E2F2; E2F1; CDC27; UBE2C; CDC23; TFDP1; AGO4; AGO1; TFDP2; AGO3		
Chromatin organization	*(148)* *MBIP; MSL2; MSL3; MSL1; ATXN7L3; BRMS1; MTA1; MTA2; MTA3; PRDM16; SETD2; SETD3; SETD6; SETD7; ASH1L; ELP5; COPRS; HDAC2; HDAC1; ZZZ3; SAP30L; ACTL6A; DR1; BRPF1; UTY; NCOA1; NCOA2; CCND1; TAF5L; CHD3; EED; TAF12; TAF10; ENY2; TADA3; TADA1; DNMT3A; RBBP4; RBBP5; RBBP7; PRMT6; PRMT5; PRMT3; ARID4A; ARID4B; ARID5B; TBL1XR1; SUDS3; ATF2; SUPT20H; ACTB; EPC1; RELA; ATF7IP; REST; SETD1B; NCOR2; NCOR1; AEBP2; KAT8; KAT7; NFKB1; SAP18; JADE1; ING4; ING5; ING3; PAX3; SAP130; TBL1X; SUZ12; CLOCK; CREBBP; KDM8; JAK2; KMT5B; KMT5A; KMT5C; KMT2E; KMT2D; KMT2A; KMT2C; KMT2B; HCFC1; WDR77; PHF20; MORF4L1;*	*(51)* *CLOCK; KMT5B; KMT2E; KMT2A; MSL1; PHF20; SETD7; MORF4L1; MORF4L2; HDAC2; BRWD1; SAP30L; PADI2; MEAF6; ACTL6A; DR1; BRPF1; RCOR1; NCOA1; TADA1; DNMT3A; RBBP5; PRMT5; ARID4B; ARID5B; TBL1XR1; SUDS3; ATF2; MECOM; SMYD2; OGT; SMARCA4; AEBP2; KAT6B; KAT6A; KAT7; SETDB2; KDM2A; KDM5A; KDM5B; KDM5C; KDM6A; KDM7A; KDM3B; ATXN7; JADE1; EP300; TADA2B; PAX3; SUZ12; EP400*	

Primary categories	B95.8 vs Ctrl	M81 vs Ctrl	M81 vs B95.8
	(#) Genes	(#) Genes	(#) Genes
	MORF4L2; BRWD1; KANSL1; KANSL2; KANSL3; PADI2; MEAF6; HIST2H2BF; HIST2H2BE; RCOR1; SUV39H2; SUV39H1; DOT1L; TAF9; YEATS4; PHF21A; YEATS2; MRGBP; MECOM; SMYD2; OGT; SMARCC1; SMARCC2; SMARCA2; SMARCA4; DPY30; CARM1; KAT14; PHF2; PHF8; KAT2B; NSD1; KAT6B; KAT6A; KDM1B; KDM2A; KDM2B; KDM5A; KDM5B; KDM5C; KDM5D; KDM6A; KDM6B; KDM7A; KDM3A; KDM3B; KDM4A; KDM4B; KDM4C; EHMT2; EHMT1; EZH2; HAT1; ATXN7; EP300; SUPT7L; TRRAP; TADA2B; BRD8; EP400; USP22		
Circadian Clock	*(38)* *NPAS2; RBM4; NCOA1; NCOA2; NCOA6; CHD9; MED1; TBL1XR1; NRIP1; BHLHE41; NFIL3; BHLHE40; NCOR1; SIK1; RXRA; SERPINE1; HIF1A; TBL1X; CLOCK; CREBBP; NAMPT; PPARGC1A; PER2; PER1; ARNTL; PPARA; CREM; ARNTL2; CRTC1; NR3C1; CRY2; CRY1; CARM1; NR1D1; RORA; EP300; CREB1; KLF15*	*(18)* *SIRT1; CLOCK; CREB1; CRTC1; MED1; HIF1A; PPARA; EP300; ARNTL; NCOA1; SIK1; TBL1XR1; CHD9; PPARGC1A; RORA; SREBF1; CREM; NFIL3*	
Developmental Biology	*(484)* *AGRN; ACTG1; HSPA8; CD36; RAPGEF2; SEMA4A; SEMA4D; ADIPOQ; DLG1; CACNA1I; DLG3; NCAN; DLG4; CALM1; TYROBP; PIK3CA; PIK3CB; CACNA1C; PIK3CD; DCC; CACNA1G; DCX; JAK2; GRIN1; CACNA1S; LYPLA2; CUL3; ARHGEF11; ARHGEF12; NGEF; SHC1; ERBB4; SHC3;*	*(122)* *NRCAM; PSMF1; SPRED1; RAPGEF2; SEMA4A; RASAL2; DLG1; CALM1; ITSN1; PIK3CA; PIK3CD; DCC; DCX; LYN; CUL3; ARHGEF11; ARHGEF12; SHC1; ERBB4; SRGAP1; CREB1; ABLIM3; KITLG; SRGAP2; KRAS; ROBO1; ROBO2; GSK3B; GRB2; GFRA1; PAK1; RASGRP3;*	*(30)* *EFNB2; RASGRP3; ACVR2B; DPYSL2; DPYSL3; ACVR2A; PSMD5; LEFTY2; FGF7; CLTC; PRKCQ; SEMA4D; FGFR3; KRAS; SEMA3A; CACNA1I; NCAM1; RGMB; CACNA1C; PRNP; DCC; ITGAV; FGF2; MAPK1; EREG; DCX; ALCAM; FURIN; NEFL; CUL3*

Primary categories	B95.8 vs Ctrl	M81 vs Ctrl	M81 vs B95.8
	**(#) Genes**	**(#) Genes**	**(#) Genes**
	CAMK2D; CAMK2G; CAMK2A; CAMK2B; ABLIM1; ABLIM3; ABLIM2; FGFR2; FGFR1; ANGPT1; SOS1; GRB10; PTPN11; RBBP7; AKAP9; RBBP5; KIT; MED6; NTN1; RBX1; WNT1; CTCF; CSF2RB; ITGA9; RASGRP1; RASGRP4; MED21; ITGA1; ITGA2; MED22; GAB2; RASGEF1A; MED27; FRS2; ITGA10; PDLIM7; CHL1; PITPNA; SREBF2; AP2S1; GAP43; ARHGEF28; EGR2; JUN; PPARGC1A; MEIS1; TIAM1; SREBF1; CDK5R1; JAK3; NF1; JAK1; KSR1; EPHB2; EPHB3; EPHB1; EPHB6; EPHB4; HOXB2; HOXB3; UNC5A; KCNQ2; KCNQ3; UNC5D; PAXIP1; NFASC; COL4A4; VEGFA; HOXB4; COL4A1; PPARA; LAMC1; PKNOX1; PLXNC1; RPS6KA1; RPS6KA2; RPS6KA3; RPS6KA4; RPS6KA5; RPS6KA6; MED13L; PSMD14; ST8SIA4; PSMD11; PSMD10; PSMD13; PSMD12; MED26; PAX6; GIT1; COL4A3; CDC42; FGF23; FGF20; COL4A2; MYH10; YY1; FGF9; PRNP; SRGAP1; RET; SRGAP2; FGF7; FGF6; RGMB; FGF5; PBX1; RGMA; NCOA2; NCOA3; APH1A; NCOA1; NCOA6; FGF2; PSMA2; HBEGF; PSMA4; MARK3; SHB; KDR; SRC; THRAP3; PSMD1; EFNA5; EFNA3; RXRA; EFNA1; DNM1; MED17; RELA; IL2RA; IL2RB; ZNF638; MYL12B; MYL12A; PLXNA3; PLXNA2; PLXNA1; NTN4; PLCG1; PLXNA4; EREG; SDC2; MYO10; CCND3; PSMD9;	*ITGA1; ENAH; FRS2; CLTC; CHL1; AP2B1; PIK3R1; IL3; DPYSL3; ARPC5; COL4A4; VEGFA; UNC5D; COL4A1; FGF7; LAMC1; FGF2; RPS6KA3; RPS6KA5; RPS6KA6; SDC2; ST8SIA4; PSMD10; ITGAV; PSMD12; COL4A3; IL17RD; ACTR3; ACTR2; EFNB2; PDGFRA; ITGA9; EPHA7; EPHA3; RET; SDCBP; HGF; ABL1; ABL2; NCAM1; HBEGF; ALCAM; CACNB4; PSMA2; MAPK3; MAPK1; DUSP9; PSMA4; DUSP5; RANBP9; ITGA2; SRC; TRIO; PAQR3; GAB1; SEMA3A; SEMA5A; SOS1; IRS1; IRS2; MET; EREG; NRP1; VAV3; PSMD5; KALRN; PSMD1; MAP2K1; WASL; SEMA6D; SPTBN1; SEMA6A; NEFL; PEA15; NEO1; DOK4; NRG1; NRG2; CLASP2; PRKCA; PSME4; PSME3; PRKCQ; RASA1; NRP2; NF1; BRAP; SHTN1; ROCK1; COL6A3;*	

Primary categories	B95.8 vs Ctrl	M81 vs Ctrl	M81 vs B95.8
	(#) Genes	(#) Genes	(#) Genes
	PSMD8; MED31; ERBB3; PSMD5; PSMD7; LIMK1; EGFR; PSMD3; ST8SIA2; MAP2K1; WASL; SPTBN4; SPTBN2; SPTBN1; H3F3A; CDK19; RAP1GAP; NEFL; KIF4A; TLN1; PEA15; KLF5; NEO1; SLIT3; YES1; SLIT1; NRG1; FGF18; PSMC2; NRG2; AJUBA; PSMC5; PSMC6; ITGB1; ITGB3; KMT2D; HOXD1; CREB1; NUMB; EPHA10; HGF; NRP1; NRP2; SCN5A; TBL1X; NCK1; NCK2; CACNA1H; CEBPD; MAP3K11; ANK2; SCN1B; COL6A3; LAMTOR3; CCNC; PCK1; EFNB3; NRCAM; FYN; PSMF1; SPTB; SPRED1; ZNF335; ARAF; RASAL1; MAFB; RASAL2; PLIN1; RARA; PEBP1; RARB; RARG; ITSN1; ARHGEF7		
DNA Replication			*(4)* *CDC7; ORC4; MCM10; MCM9*
Expand All Signal Transduction (Wnt)			*(3)* *FZD5; AMER1; CAV1*
Extracellular matrix organization	*(36)* *PLEC; COL27A1; COL1A1; COL1A2; DST; COL15A1; CD151; TLL2; TLL1; COL7A1; LOX; COL6A3; COL9A1; COL9A2; BMP1; LAMC2; LAMA3; LAMB3; COL8A2; COL3A1; ITGB4; ITGA6; LOXL1; COL10A1; COL2A1; COL24A1; COL11A1; COL5A1; COL5A3; COL5A2; COL4A2; COL4A1; COL4A4; COL4A3; COL4A6; COL4A5*	*(32)* *COL8A2; TGFB2; COL11A1; PRKCA; ITGB8; ITGA2; FBN2; COL19A1; FBN1; COL5A2; MFAP5; COL4A4; COL4A3; ITGAV; COL4A1; FBLN2; TNC; COL3A1; MFAP1; LOX; COL17A1; FGF2; BMP2; CASK; SDC2; COL14A1; GDF5; THBS1; COL21A1; MFAP3; COL6A3; COL12A1*	
Gene expression (Transcription)	*(352)* *MOV10; SUMO1; PRKAG2;*	*(145)* *YWHAQ; PRKAG3; PRKAG2;*	*(10)* *HDAC2; ATM; PRKAA2;*

Primary categories	B95.8 vs Ctrl	M81 vs Ctrl	M81 vs B95.8
	**(#) Genes**	**(#) Genes**	**(#) Genes**
	TIGAR; NELFE; HIPK2; HIPK1; PMAIP1; CCNT2; EP300; NR0B2; CCNC; RORC; RORB; RORA; THRA; COX20; TAF4B; PERP; RNF111; RRM2B; NRBF2; BRCA1; NELFA; WRN; SP1; PRMT5; NR2C2; NR2C1; GATA4; MDM4; RBBP8; GTF2F1; RBBP7; KIT; RUNX2; PTEN; NR2E1; MED20; CDKN1A; NOP2; UBE2D3; MED27; UBE2D1; RAD1; NDRG1; RICTOR; SKI; SERPINE1; SMURF2; MAML3; PPM1A; MAML1; FOS; JUN; NR2C2AP; TRIAP1; CDK5R1; SURF1; CHEK2; CHEK1; PRKAB2; PRKAB1; RAD50; PARP1; BLM; PPARD; VEGFA; PPARA; ING5; PHF20; ING2; UBE2I; ESR2; ESR1; ATAD2; NRBP1; PRDM1; GADD45A; TP53INP1; TFDP2; TFDP1; PPP2R5C; YY1; SESN1; SESN2; SMAD4; THRB; MED12; SMAD7; POLR2D; SMAD2; SMAD3; STK11; TNFRSF10B; TAF11; DYRK2; NR4A2; TNFRSF10D; TP53RK; TSC1; NCOA2; NCOA1; NCOA6; ZNF385A; NR2F6; NDUFA4; BNIP3L; RXRA; RXRB; TCEA1; COX4I1; TNKS1BP1; JMY; TP53; YAP1; TBL1XR1; SGK1; TP73; TAF1L; KMT5A; RHEB; GLS2; COX5A; BIRC5; EGFR; CYCS; USP9X; MAP2K6; TEAD3; TEAD1; RNF34; DNA2; CDK12; CDK13; TFAP2C; TFAP2B; MAPKAP1; TMEM55B; CNOT8; PGR; CNOT2; CNOT1; CNOT7; CNOT6; PIN1; CNOT4; ESRRG; ESRRA; BANP; LAMTOR5; E2F7; SETD9; E2F5; E2F4;	*CASP10; HIPK1; TNRC6C; TNRC6B; TNRC6A; CCNT2; DGCR8; RARB; RAN; THRB; RORB; RORA; COX20; TAF4B; MED13; RNF111; BRCA1; PLK2; BARD1; SP1; TDRD6; PRMT5; NR2C2; TPR; KCTD15; CNOT6; RUNX2; PTEN; CCNC; KDM5B; TSN; POM121C; NUP58; TRIM33; NUAK1; CDKN1A; UBE2D3; MED1; RAD1; PRKAB2; RICTOR; TGFA; SMURF2; PPM1A; CHD9; JUN; RRM2B; NR5A2; H3F3A; TXNRD1; POM121; PRKAB1; NCOA1; VEGFA; SMYD2; PPARA; PHF20; BTG2; UBE2I; TAF5; TAF4; ESR1; PRDM1; IPO8; COX11; AGO1; TFDP2; TFDP1; AGO2; HDAC2; SMAD8; SESN1; SMAD4; ATM; SMAD2; SMAD3; MAPK14; CCNG1; DYRK2; TBX5; GLS; TSC1; TP63; DROSHA; NR6A1; CITED2; MSH2; APAF1; RANBP2; NDUFA4; BNIP3L; PRKAA2; SMAD7; EP300; YAP1; CNOT6L; NUP50; DICER1; TBL1XR1; MTF2; CTGF; TAF9B; BIRC5; JARID2; PHF19; PRKAA1; PITX2; USP9X; AEBP2; MYBL1; RNF34; NR1D2; DNA2; CDK12; CDK13; TFAP2B; NUP43; MAPKAP1; BRPF1; CNOT8; MED14; SUZ12; CNOT2; YWHAG; DNMT3B; ESRRG; DNMT3A; NR4A2; NR4A3; GTF2H1; CHM; POU4F2; JMY; E2F5; MEAF6; NOTCH2; NEDD4L; FAS; KAT6A; PPP2R5C; TEAD1; ATF2*	*DYRK2; MTOR; PRDM1; RAD1; RICTOR; PHF20; PIP4K2B*

Primary categories	B95.8 vs Ctrl	M81 vs Ctrl	M81 vs B95.8
	**(#) Genes**	**(#) Genes**	**(#) Genes**
	TBL1X; E2F1; RPA1; RPA2; RABGGTB; TFAP2A; COX16; FAS; BCL6; RAD17; LRPPRC; PLK2; CASP10; AKT1; TNRC6C; TNRC6B; TNRC6A; MDM2; NKX2-5; RARA; RARB; RARG; RBPJ; BRPF1; TP53AIP1; MYC; PIP4K2A; PIP4K2B; CDKN2B; CDKN2A; DDIT4; CENPJ; RFFL; CHM; POLR2C; CSNK2A1; KCTD15; TAF12; AGO4; NR1I3; UBA52; TAF10; YEATS4; UBC; PLAGL1; KDM5B; CCNK; VDR; ATP1B4; NR3C2; TRIM33; TBX5; MED1; MED6; LAMTOR1; MED8; RBBP4; CRADD; TGFA; L3MBTL1; CHD3; GLS; CHD9; EXO1; SUPT4H1; ATRIP; NR5A2; SFN; TXNRD1; TTC5; EHMT1; CTGF; CDC25C; MED31; IGFBP3; POLR2G; POLR2F; POLR2A; SMYD2; POLR2H; NR6A1; TAF7; TAF5; TAF4; TAF3; TAF2; TAF1; TBP; HNF4G; G6PD; HNF4A; TAF9; AIFM2; COX11; AGO1; CREBBP; AGO2; HDAC1; AGO3		
Hemostasis	*(68)*	*(63)*	
	MAPK14; TRPC3; TRPC6; TRPC7; CALM1; PDE3A; GUCY1A3; FGR; PDE3B; GUCY1A2; SLC8A1; SLC8A3; SLC8A2; MRVI1; SRI; GNB1; ATP2B1; ATP2B3; ATP2B2; ATP2B4; KCNMB1; KCNMB2; KCNMB4; LRP8; NOS1; GNG2; PDE5A; GUCY1B3; PTPN11; STIM1; RASGRP1; ATP2A2; ATP2A3; MGLL; NOS3; P2RX1; P2RX5; P2RX4; P2RX7; P2RX6; DGKZ; APOB; PDE1B; PDE1A; DGKQ; KCNMA1;	*TGFB2; TIMP3; NOS1; PRKCQ; PROS1; RAP1A; RAPGEF4; HGF; STXBP3; ADRA2B; F13A1; CRK; APLP2; PDE3B; GNAI3; GNAI1; DGKZ; GUCY1A3; CALM1; PIK3CA; PIK3CD; KCNMA1; PDPN; PIK3CG; MAPK3; SPARC; DGKI; PRKG1; TTN; PIK3R1; DGKB; LYN; FGG; PDE5A; DGKE; SRC; MRVI1; SHC1; PRKCA; PRKCB; GNB1; ANGPT2; OLA1; VEGFA; CD36; ITPR2; LCP2; PTPN1; ITPR1;*	

Primary categories	B95.8 vs Ctrl	M81 vs Ctrl	M81 vs B95.8
	**(#) Genes**	**(#) Genes**	**(#) Genes**
	ITPR2; DGKH; DGKI; PRKG1; PRKG2; DGKB; DGKA; DGKG; DGKD; DGKE; PRKCH; NOS2; ORAI1; PRKCE; PRKCD; PTGIR; PRKCQ; ITPR3; PLA2G4A; ITPR1; PAFAH2; GNAS	*KRAS; MAPK14; SOS1; THBS1; CD109; PCDH7; F2R; GRB2; GNA12; IL3; MAPK1; GNA13; VAV3; LAMP2*	
Immune System	*(568)*	*(222)*	*(22)*
	MOV10; CRKL; SUMO1; HSPA8; IL6ST; IFNAR2; IFNAR1; LIFR; DLG4; TYROBP; PIK3CA; PIK3CB; PIK3CD; LAMTOR3; IRAK2; IRAK3; GRIN1; IRAK1; IFNA16; IRAK4; IFNA14; DHX9; NUP98; CAMK2D; CAMK2G; CAMK2A; CAMK2B; CSF3R; CXCL10; GATA3; CCND1; RBSN; AKAP9; PTEN; IL24; IL7R; TNFSF11; FBXW11; RASGRP4; CDKN1B; CDKN1A; MX2; UBE2D3; HSP90B1; MX1; IFIT3; IFIT2; GRAP2; JAK2; JAK3; NF1; JAK1; KSR1; CCL22; EDARADD; TNFSF13; CHUK; VEGFA; CNTFR; ZEB1; RPS6KA1; RPS6KA2; RPS6KA3; RPS6KA5; PSMD14; PSMD11; PSMD10; PSMD13; PSMD12; SIRPB1; FLNB; IL15RA; EIF4A2; IL12RB2; CD40LG; NUP133; RET; ITPR2; NDC1; ITPR3; MT2A; PIK3AP1; TNFSF13B; CLEC5A; ITPR1; PELI1; PELI3; PELI2; CIITA; NEDD4; BRWD1; PSMD7; IL17A; CTF1; EGFR; LTB; OPRM1; FGF23; PRKAR1A; PRKAR1B; SH2B1; UBE2V1; IL2RA; IL2RB; EDA; FOXO1; EBI3; CDC42; PSMD9; PSMD8; HLA-DPB1; PSMD5; HMGB1; MAP2K4; PSMD1; PSMD3; PLCG1; VRK3; TWIST1; PDE1B; PDE1A; PEA15;	*PTGS2; GHR; PSMF1; SPRED1; CD36; IL6ST; IKBKB; MUC20; IL22; TNRC6C; TNRC6B; TNRC6A; RASAL2; MS4A2; MAP3K8; EP300; PPP3R1; CALM1; PIK3CA; MAP3K1; PIK3CD; RORA; CUL8; CUL9; IRAK2; CUL5; LYN; CUL7; CUL1; CUL2; CUL3; SHC1; ERBB4; CREB1; TPR; MUC19; KITLG; CTSS; MUC13; KRAS; SOCS5; SOCS3; SOCS2; CXCL8; ADCY3; ADCY9; GSK3B; PTEN; GRB2; GFRA1; KPNA1; PAK1; RAPGEF1; RASGRP3; POM121C; FBXW11; RAPGEF2; POU2F1; CDKN1A; UBE2D2; UBE2D3; HIF1A; CSF1; BCL2; CRK; RICTOR; IFIT3; IFIT1; PPM1B; CLEC7A; PRLR; EGR1; JUN; MAPK19; TNFRSF8; PIK3R1; EREG; FGG; PRKCB; POM121; EDAR; CHUK; IL18R1; RAG1; VEGFA; IL7; FGF7; PTPN2; PTPN1; ITPR1; ZEB1; FGF2; CUL10; PTPN4; EDA2R; IRF1; RPS6KA3; RPS6KA5; UBE2N; MAPK21; MAPK20; VAV3; MAPK22; PSMD10; CBL; PSMD12; MEF2C; MEF2A; MAP3K3; FLNB; IL17RD; AGO1; PPP2R5C; IFNAR1; PDGFRA; NFATC2; RET; MAPK14; ITPR2; MAPK16; MAPK17; MAPK10; CCR5;*	*RASGRP3; PSMD5; TNRC6B; RAP1B; MTOR; PRKAR1A; FGF7; RICTOR; RAP1GAP2; FGFR3; KRAS; ADCY7; NCAM1; ADCY1; NEFL; FGF2; MAPK1; PRKG1; EREG; PRKACB; CUL3; PIP4K2B*

Primary categories	B95.8 vs Ctrl	M81 vs Ctrl	M81 vs B95.8
	(#) Genes	(#) Genes	(#) Genes
	SPTBN1; PSMC2; PSMC5; PSMC6; ITGB1; CNTF; NOS2; ARIH1; CD44; KPNB1; CD40; IL33; HGF; LCP2; EIF4E3; EIF4E2; KLRK1; BTRC; NUP35; IER3; MAP3K11; MAP3K14; LIF; IL1RL1; GHR; SPTB; SPRED1; IKBKB; PTK2B; TNRC6C; TNRC6B; TNRC6A; IKBKE; RASAL1; RASAL2; PEBP1; IFNLR1; PRL; FADD; RELA; PIP4K2A; PIP4K2C; PIP4K2B; ADCY6; CLCF1; ADCY7; TPR; KITLG; NRG2; FN1; APP; CSF1; VIM; UBA52; ELK1; PHB; PAK1; PAK2; PAK3; CCL2; POM121C; IL10RA; IL10RB; CCL5; DHX36; RSAD2; TIRAP; ADAR; ANGPT1; RASGEF1A; EDAR; MAP2K6; UBE2E1; RAG1; EIF4E; SARM1; IRF2; IRF1; IRF6; IRF5; IRF4; IRF9; IRF8; MEF2C; TRIM8; MEF2A; TRIM5; PDGFRB; PDGFRA; PRKAR2A; RASGRP1; FLT3; NUP62; TNFSF15; SKP1; S1PR1; CSF1R; TNFSF9; MAPK1; RANBP9; TNFRSF6B; TNFSF4; UBE2D2; DAB2IP; IL6R; PML; MTOR; DUSP4; TRIM62; FRS2; DUSP7; MAPK9; PIP5K1C; PIP5K1B; STX3; IRS1; IRS2; KL; RANBP2; PTK2; MCL1; SOX2; F13A1; SMARCA4; YWHAZ; INPPL1; CEBPD; FYN; NOD1; NOD2; YWHAB; RAC1	*MAPK12; MAPK13; IL1A; MAPK18; NCAM1; PIK3AP1; AGO2; STAT3; STAT1; PELI2; CUL12; S1PR1; TEC; NEDD4; PSMA2; MAPK3; MAPK1; DUSP9; PSMA4; IL15; DUSP5; RANBP9; CUL11; MAPK15; RANBP2; SRC; PAQR3; TLR4; FOXO1; CUL4; LTA; GAB1; CNTFR; CUL6; NUP50; SOS1; MAPK9; IFNA1; NUP58; IRS1; IRS2; IFNA4; MET; IL3; PRKACB; BRWD1; IFNA14; LIFR; MAPK23; PSMD5; BIRC5; PRKCQ; PSMD1; BIRC2; MAPK24; FOXO3; MAP2K1; IL24; F13A1; SMARCA4; SPTBN1; MID1; TBK1; EEA1; NEFL; FRS2; HBEGF; EIF4E; MAPKAP1; PEA15; MAPK11; NRG1; NRG2; NUP43; ARIH1; PRKCA; CD44; PSME4; KPNB1; PSME3; TRIM22; IL1RAP; HGF; LCP2; RASA1; NF1; BRAP; CASP3; TAB3; TRIM5; BTRC; CCR1; IRF4; PPP3CA; PPP3CB; EIF4A2; PIP4K2C; HAVCR2; ATF2*	
Metabolism	*(605)*	*(130)*	*(31)*
	AGL; AGK; PRKAG2; PCSK6; PCSK5; PCSK9; FDXR; HPSE2; SQLE; ADIPOQ; PIP4K2A; SGPL1; NCAN; NPC2; PIK3CA; PIK3CB; CCNC; PIK3CD; PIK3CG; SPTLC2; SPTLC3;	*PTGS2; PTGS1; AGK; PRKAG2; CD36; OSBPL1A; DAB1; CAV1; CERS6; SGPL1; GPAM; CERS2; RAN; PIK3CA; PIK3CD; INPP4B; RORA; PIK3CG; MTMR7; MED14;*	*SLC44A1; SOAT1; VLDLR; PIP4K2B; DLD; PRKACB; PRKAA2; BCAT1; PLEKHA1; DHTKD1; PDP1; PCSK9; PRKAR1A; CLTC; ARL2BP; CAV1; ADCY7; PPP1CB;*

Primary categories	B95.8 vs Ctrl	M81 vs Ctrl	M81 vs B95.8
	(#) Genes	(#) Genes	(#) Genes
	GLP1R; ABCD1; SLC27A2; VAC14; B3GALT6; STK11; NUP98; SP1; PIK3C2B; PIK3C2A; AP2B1; PIK3C2G; CD36; GK; AKAP5; GM2A; AHR; PTEN; PLTP; ACLY; TXNRD1; HSD17B12; GPCPD1; UGCG; ALDH3A2; LIPE; APOA5; SPTSSA; CHKA; SPTSSB; ARNTL; APOB; DGAT1; DGAT2; LSS; CAV1; HMMR; CERS6; CHST9; PPARD; CERS5; INSIG2; PGM2; PPARA; CHST1; PPP1R3C; CHST3; CHST2; CSGALNACT2; CHST7; CSGALNACT1; ZDHHC8; CERS1; PSAP; ABCB11; LDLRAP1; GPX1; SLC35B2; OSBP; HS3ST3B1; SOAT1; NUP133; CROT; VAPB; VAPA; SC5D; FDX1; RORA; CDIPT; NCOA2; NCOA3; NCOA1; NCOA6; HS3ST1; ARF3; ARF1; SPTLC1; RXRA; LDLR; PRKAR1A; PRKAR1B; MCAT; SAR1B; SLC27A1; YAP1; ALB; ABCA1; PRKD1; PRKD2; PRKD3; SPHK2; G6PD; KCNC2; HS2ST1; CHP1; LTA4H; SGMS1; GYG1; GYG2; ACER2; ACER3; PGD; CDK19; LPIN2; LPIN1; ESYT2; ESYT3; HSD17B8; TMEM55B; HSD17B2; HSD17B4; IDH1; PFKM; ALDH3B1; CD44; KPNB1; STS; PLA2G4C; PLA2G4A; PLA2G4F; CRAT; TBL1X; ARSB; HEXA; HEXB; NUP35; ARSJ; ARSK; OGN; FAR1; CYP11B1; PCK2; PCK1; GNPDA2; GNPDA1; OSBPL1A; DAB1; ARL2BP; SLC25A11; LBR; B3GNT2; RAN; ADRA2A; GNG12; RELN; MTMR7;	*MTMR4; MTMR2; MTMR1; MTM1; SYNJ2; SEC24D; PIK3C2A; SEC24A; GK; ABHD5; OSBPL10; PCYT1B; PLD1; PLD2; MBTPS1; ACSL3; ACSL1; ACSL4; AHR; PTEN; PLEKHA1; CCNC; MMAA; FDX1; HSD17B12; INPP5K; MED21; MED1; CLTC; MED9; SPTSSA; CHKA; ARNTL; CHD9; PPARGC1A; PCTP; PIK3R3; PIK3R1; PNPLA4; TXNRD1; AP2B1; PRKAB2; PIKFYVE; LBR; INSIG1; HACD2; MED13L; CYP8B1; ELOVL6; ELOVL5; ELOVL2; MSMO1; AGPS; CLOCK; PPARA; VAPA; INPP4A; FABP1; HPGD; NCOA1; ARF3; ALDH3A2; SRD5A3; SRD5A2; RAB14; GPCPD1; PLPP3; THRAP3; PDSS2; PDSS1; FADS1; EP300; STARD3NL; YAP1; TBL1XR1; ARNT; MTF1; CTGF; SREBF1; PIP4K2C; PRKACB; PRKD1; PRKD3; SCP2; PRKAA2; NFYA; GPD2; TEAD1; CDK19; LPIN1; DDHD1; ESYT2; ACADM; MED13; SYNJ1; SP1; MED18; HSD11B1; SLC44A1; KPNB1; STS; INPP1; PPP1CB; ARSB; IMPA1; HEXB; CEPT1; ARSJ; HADHB; FAR1*	*ADCY1; CACNA1C; INPP4A; MTMR4; GNG3; SYNJ2; GLP1R; PDHB; FURIN; MTMR3; DBT; ABCG5; FABP3*

Primary categories	B95.8 vs Ctrl	M81 vs Ctrl	M81 vs B95.8
	(#) Genes	(#) Genes	(#) Genes
	MTMR6; MTMR4; MTMR3; MTMR2; PIP4K2C; PIP4K2B; LIPA; RAB5A; EXT2; EXT1; SNAP25; SYNJ2; XYLT2; SCD5; SEC24D; TNFRSF21; PCYT1B; SEC24A; TPR; SEC24C; SEC24B; ORMDL2; PLD1; DBI; PRKAR2A; PLD2; PTGES3; SCARB1; SLC2A4; SLC2A2; SLC2A1; GNG7; GNG4; GNG5; GNG2; GNG3; PLEKHA2; PLEKHA3; MMAA; PLEKHA8; POM121C; VLDLR; ACOX2; MED1; MED6; PLA2G1B; MED8; MED9; NUP153; PLA2G12A; HPGD; TKT; BMX; PHKG2; PKM; MED28; GCK; CYP1B1; HACD2; CYP27A1; HS6ST1; HS6ST2; CREBBP; ORMDL3; PI4KB; PI4KA; CLOCK; COQ9; STXBP1; PRKAR2B; SMPD1; FABP3; SMPD3; PGAM1; AKR1C3; NUP62; PNPLA2; CHPT1; PRELP; ORMDL1; RAB14; HAS3; PLPP1; PLPP3; MTMR14; PLA2R1; GNB4		
Metabolism of proteins	*(553)*	*(174)*	*(7)*
	PGAP1; UBE2Q2; CPM; SUMO1; SUMO3; SUMO2; SEC23IP; OTUD7B; NTNG1; CDC73; CTAGE5; NAPA; PRND; THY1; HSPA8; NUP98; WRN; SP3; MUC7; MUC4; GALNT15; MUC19; MUC17; MUC13; GATA3; COL7A1; TRAPPC1; TRAPPC3; PTEN; EIF5A2; TNKS; OS9; UBE2D2; UBE2D3; UBE2D1; GPLD1; WDR48; TADA2B; CETN2; GMDS; OPCML; FURIN; B3GLCT; SENP5; BRCC3; SENP2; SENP1; BLM; CHST8; CHST4; PSMD14; PSMD11; PSMD10; PSMD13; PSMD12;	*SCFD1; PGAP1; DYNLL2; PSMF1; UCHL5; MUC20; SEC23IP; USP25; TMED10; ADAMTS5; B3GNT5; NTNG1; CALM1; TUSC3; CTAGE5; PHC1; PHC3; BRCA1; SMAD7; BARD1; CTSC; CHM; NEGR1; SEC24D; SEC24A; TPR; MUC19; EDEM1; SMAD3; MUC13; MAN1A1; USP37; ARCN1; PTEN; CYLD; APC; EIF5A2; TNKS; POM121C; ALG9; FOXK2; FOXK1; ARSB; UBE2D2; UBE2D3; HIF1A; ALG5; CNTN3; ARFGAP2; CNTN4; SEC16A; STAM; SUDS3; TGFA; SMURF2;*	*PSMD5; YY1; INO80D; OGT; ASXL2; KDM1B; SMAD7*

Primary categories	B95.8 vs Ctrl	M81 vs Ctrl	M81 vs B95.8
	(#) Genes	(#) Genes	(#) Genes
	GOSR2; ATXN7; GFPT2; GOSR1; ATXN3; DAG1; GANAB; DYNC1LI2; NUP133; NRN1; SMAD4; SMAD7; OTUD3; SMAD1; SMAD2; SMAD3; TAF10; DCTN3; DCTN6; RHOA; DCTN4; DCTN5; NEDD8; SMC3; SMC5; ARF4; RNF152; NUS1; ALG11; SAR1B; ALG13; NFRKB; RNF20; F8; F9; C1GALT1C1; UBE2L3; NTM; IFIH1; PSMD9; PSMD8; PSMD5; EEF1A1; PSMD7; PSMD1; PSMD3; RAB2A; MITF; CCNA2; CCNA1; DYNC1I1; DYNC1I2; XPC; PRMT3; PSMC2; PSMC5; PSMC6; SPON1; MDC1; HGS; IL33; STS; MCFD2; ST6GALNAC4; ARSB; OGT; TFG; NUP35; ARSJ; ARSK; MBD5; ANK1; ANK2; MBD6; TFAP2C; EEF2; FUT8; TRIM13; TUSC3; SPTB; MARCH6; USP28; USP25; USP24; USP22; USP20; ADAMTS5; B3GNT5; B3GNT2; MDM4; CDKN2A; RAB5A; CHM; UBE2R2; SEC24D; SEC24A; TPR; SEC24C; EDEM1; UBA52; CYLD; EID3; RNF103; APC; ACTB; MAVS; MAN1C1; SNX3; CALR; POM121C; ALG9; ALG2; PROS1; SUDS3; CNTN3; ARFGAP2; ARFGAP3; MDGA2; ARFGAP1; STAM; COPZ1; DDX58; ADAMTSL1; POM121; RAB11A; DERL1; DERL2; RAB11B; CEACAM5; CEACAM7; TNKS2; INCENP; VNN3; VNN1; UBE2E1; UBE2E3; BMI1; SCMH1; GALNT4; F10; GALNT7; GALNT1; GALNT2; GALNT3; PROC; SUMF1; HDAC7; PIGW;	*USP48; WDR48; TADA2B; ASXL2; THBS1; DERL1; CEACAM5; B4GALT5; B4GALT6; SMC1A; POM121; LARGE1; SENP5; MAN2A2; SENP2; UBA6; HERC2; DAG1; DYNC1LI2; GALNT4; RAD21; GALNT7; GALNT3; UBE2I; UBE2K; UBE2N; UBE2B; PSMD10; TOP1; PSMD12; ATXN7; GOSR1; UBE2W; PPP6R3; RAD23B; MIA3; SMAD4; USP15; OTUD3; SMAD1; SMAD2; PRKDC; RHOT1; TMED7; BET1; TMED2; SMC5; SMC6; PSMA2; PSMA4; SRD5A3; ANKRD28; DPM1; RANBP2; TGFBR2; SEL1L; CD59; RAB1A; PROS1; WAC; AMFR; JOSD1; EP300; NUP50; B3GALNT2; GALNT10; CD109; ESR1; YOD1; NTM; RAD52; RNF144A; STAMBPL1; TAF9B; USP8; RECK; ST8SIA4; MDGA2; STS; CD55; PSMD5; BIRC5; USP3; PSMD1; BIRC2; USP9X; MITF; BMI1; SPTBN1; CNIH1; DYNC1I1; STAM2; TFAP2B; NUP43; UBE2G2; UBE2G1; OTULIN; NUP58; SP3; GALNT13; PSME4; PIGK; PDIA3; PSME3; USP10; MGAT3; LMAN1; RTF1; LMAN2; INO80D; DPH6; OGT; CALR; ARSJ; ASXL1; MBD6; ACTL6A; USP47; FUT8*	

Primary categories	B95.8 vs Ctrl	M81 vs Ctrl	M81 vs B95.8
	(#) Genes	(#) Genes	(#) Genes
	MAGT1; CBX4; CBX2; AREG; BET1; NUP62; NLRP3; RNF128; TRAPPC10; WDR20; RAB13; RANBP2; TGFBR2; TGFBR1; DPAGT1; RAB1A; PAF1; RAB1B; YKT6; PML; ASGR2; JOSD1; EP300; CD109; STX5; YOD1; KEAP1; TRAPPC6B; RAD52; NSMCE2; NSMCE3; TAF9B; RCE1; NEU1; RNF146; USP8; ST6GAL1; USP3; USP4; USP7; ETFB; OTUB1; UIMC1; CNIH1; RABGGTB; CNIH3; CNIH2; NSMCE4A; AXIN2; ANK3; NOD1		
Metabolism of RNA		*(15)* *POM121C; SLBP; POM121; NCBP1; NUP58; NUP43; EIF4E; NUP50; TPR; WDR33; IGF2BP1; FIP1L1; IGF2BP3; CPSF2; RANBP2*	
Muscle contraction	*(87)* *KCNE1; FXYD2; FXYD3; HIPK2; HIPK1; GATA4; NKX2-5; CACNA2D1; AHCYL1; CALM1; RYR3; RYR2; CACNA1D; DMPK; CACNA1S; NPR2; CAMK2D; CAMK2G; ATP1B2; CAMK2A; CAMK2B; ATP1B1; CORIN; ATP2B1; CACNA2D3; ATP2B3; ATP2B2; ATP2B4; CACNA2D4; KCNJ12; AKAP9; NPPC; FKBP1B; ATP2A2; ATP2A3; KAT2B; KCND3; CACNA2D2; PRKACA; KCNIP4; KCNIP3; KCNIP2; KCNIP1; ORAI1; ITPR2; ITPR3; ITPR1; KCNH2; SCN5A; CACNA1C; TRPC1; TBX5; CACNB3; CACNB2; CACNB1; ASPH; CACNB4; SLC8A1; FGF12; SLC8A3; SLC8A2; FGF14; CLIC2; SRI; FGF13; FGF11;*		

Primary categories	B95.8 vs Ctrl	M81 vs Ctrl	M81 vs B95.8
	(#) Genes	(#) Genes	(#) Genes
	KCNJ2; WWTR1; ATP1A4; ATP1A3; ATP1A2; ATP1A1; PLN; STIM1; TRDN; KCNK3; KCND1; NOS1; KCNJ11; KCND2; KCNJ14; ATP1B3; SLN; SCN1B; ABCC9; CACNG2; CACNG4		
Neuronal System	*(225)*	*(44)*	*(4)*
	SLC6A3; LIN7C; LIN7B; SLC6A4; HSPA8; LRFN2; LRFN1; KCNK2; GABRB2; DLG1; DLG3; DLG2; GNAI3; DLG4; ARHGEF9; CACNA1A; CACNA1E; GNG12; DLGAP2; DLGAP1; DLGAP4; GRIN1; SYT9; CAMK2D; CAMK2G; KCNAB1; CAMK2A; CAMK2B; ADCY7; KCNN3; TSPAN7; MDM2; ADCY1; KCNMB1; AKAP5; ADCY6; KCNMB2; DBNL; KCNMB4; ADCY2; ADCY3; HCN1; SYT1; HCN3; HCN2; NRXN3; HCN4; NRXN1; SYT7; GNG7; GNG4; GNG5; GNG2; GNG3; GRIP1; ALDH5A1; SYT2; KCNV1; KCNJ8; GRIK3; PLCB1; GRIA2; GRIA3; KCNG3; KCND3; GRIA4; EPB41; RRAS; GABRA5; KCND2; TSPOAP1; GABBR1; GABBR2; AP2B1; SLC22A2; AP2S1; GABRA6; NCALD; RAF1; KCNMA1; GAD1; SYT12; SYT10; GRM5; GNAI1; GRM1; PDLIM5; NLGN4X; KCNQ4; KCNQ5; KCNQ2; KCNQ3; NRXN2; KCNH5; KCNH4; KCNH7; RPS6KA3; KCNH1; RPS6KA6; KCNH2; DNAJC5; CALM1; KCNH8; CAMKK1; FLOT2; GNAL; SLC18A2; STX1A; GABRR1; SLC32A1; AKAP9; STXBP1; KCNA6; KCNA7; KCNA4; GLS; GNAI2; KCNA3;	*SLC6A3; LIN7C; PLCB1; GRIA2; GABRG2; MYO6; CASK; GABRA5; GABRA4; GABBR2; GLS; GABRA1; GNAI3; GNAI1; DLG1; CALM1; NEFL; CACNA1E; GNG12; GAD1; MAPK1; GRIA3; AP2B1; KCNJ12; KCNJ15; PRKCA; PRKCB; GNB1; CREB1; GABRB2; GABRB3; KCNJ2; CACNB4; RPS6KA3; RPS6KA6; ADCY3; NCALD; ADCY9; GNG4; PRKACB; ALDH5A1; SLC1A1; SLC1A2; SLC1A3*	*ADCY1; NEFL; MAPK1; PRKACB*

Primary categories	B95.8 vs Ctrl	M81 vs Ctrl	M81 vs B95.8
	(#) Genes	(#) Genes	(#) Genes
	GABRA3; KCNA1; GABRA1; SNAP25; CACNB3; ADCY5; CHRNB2; CASK; CACNB4; AP2M1; MAPK1; RASGRF2; GABRG2; SLC6A12; RIMS1; EPB41L1; EPB41L3; EPB41L2; EPB41L5; NLGN3; NLGN1; ARL6IP5; GABRA4; GNB1; RPS6KA2; GNB3; GNB2; CHRNA4; CHRNA5; GRIA1; UNC13B; CHRNA2; ACHE; KCNB1; GABRB3; KCNJ3; KCNJ2; KCNJ1; APBA1; KCNJ6; KCNJ5; GRIK1; GRIK2; SLC17A7; PICK1; ADCY9; PRKACB; GLS2; KCNC4; KCNC1; KCNC2; ABAT; SLC5A7; NLGN2; PPFIA4; PPFIA2; KCNK9; PPFIA1; NEFL; NSF; KCNK3; PTPRD; KCNS1; KCNS2; KCNS3; HOMER1; HOMER2; CREB1; GNGT2; KCND1; KCNJ12; KCNJ11; KCNJ10; KCNJ16; KCNJ15; KCNJ14; PRKCA; NLGN4Y; PRKCB; RTN3; CPLX1; AP2A1; KCNAB3; RAB3A; AP2A2; NPTN; RPS6KA1; SHANK3; SHANK2; PPFIA3; ACTN2; VAMP2; MYO6; GRIN2B; PTPRF; GRIN2A; ABCC8; ABCC9; GRIN2D; CHRNE; PDPK1; CACNG2; SLC1A7; CACNG4; SLC1A1; SLC1A2; SLC1A3		
Organelle biogenesis and maintenance		*(19)*<br>*PPRC1; PRKAB2; PRKAB1; PRKAG3; PRKAG2; PRKAA2; CREB1; MAPK14; MED1; PPARA; GABPB1; CRTC1; NCOA1; TBL1XR1; CHD9; PPARGC1A; MEF2C; GABPA; ATF2*	*(5)*<br>*GABPB1; MEF2C; PRKAA2; CRTC2; CYCS*
Others	*(563)*	*(185)*	*(3)*

Primary categories	B95.8 vs Ctrl	M81 vs Ctrl	M81 vs B95.8
	(#) Genes	(#) Genes	(#) Genes
	MEF2C; PCSK9; AGK; CHRNA2; RAB4A; PRKAG2; PCSK6; AGER; CD36; PCSK5; NR1D1; PPP1CB; GGPS1; FDXR; SQLE; CAV1; CERS6; SGPL1; CERS5; CERS2; CERS3; CERS1; PIK3CA; PIK3CB; MAP3K1; PIK3CD; MAP3K7; RORA; PIK3CG; PLA2G6; SPTLC2; SPTLC3; SPTLC1; IRAK2; IRAK3; GRIN1; IRAK1; VAC14; CUL1; IRAK4; DHX9; ADCY7; VRK3; CAMK2D; SP1; IDH1; CAMK2G; CAMK2A; CREB1; AHRR; TNIP2; PIK3C2B; PIK3C2A; PIK3C2G; HMGCR; ACOT7; MDM2; GK; BMX; MTMR3; MAPKAPK2; ADCY5; ADCY6; PCYT1B; ORMDL2; PLD1; PLD2; ORMDL1; PTPN13; DUSP6; INPP5J; GM2A; AKAP9; AHR; PTEN; PLTP; KCNJ6; ACOX3; ACLY; FDX1; GABRB2; GRIK2; ADCY1; HSD17B12; MED28; UGCG; FBXW11; GBA; ALDH3A2; MED20; MED23; MED22; UBE2D2; UBE2D3; MED27; MED26; ACSBG1; ACSBG2; APOA5; GABBR2; SPTSSA; CHKA; SPTSSB; ARNTL; AP2S1; SUMF1; APOB; NCALD; DGAT1; DGAT2; JUN; PPARGC1A; PPARGC1B; GRIK1; GNG4; PNPLA2; LSS; PNPLA4; PRKACA; FURIN; DLG1; PRKAB2; ACADM; FOS; CHUK; PPARD; GPAM; INSIG2; PPARA; ARNT2; DLG4; RPS6KA1; RPS6KA2; RPS6KA3; RPS6KA5; RPS6KA6; UBE2N; MED13L; ZDHHC8; CALM1; PSAP; GRIA2; LDLRAP1; GPX1;	*PTGS2; PTGS1; AGK; PRKAG2; CD36; IKBKB; OSBPL1A; DAB1; CAV1; DLG1; MAP3K8; LBR; CERS2; CALM1; RAN; PIK3CA; MAP3K1; PIK3CD; INPP4B; RORA; PIK3CG; IRAK2; SYNJ1; MTMR4; CUL1; MTMR1; MTM1; CREB1; SEC24D; PIK3C2A; SEC24A; GK; ABHD5; OSBPL10; PCYT1B; PLD1; PLD2; MBTPS1; ACSL3; ACSL1; ADCY9; ACSL4; AHR; PTEN; GNG4; LPIN1; PLEKHA1; TLR4; CCNC; MMAA; FDX1; HSD17B12; FBXW11; KCNJ12; ALDH3A2; GRIA3; UBE2D2; UBE2D3; MED1; CLTC; GABBR2; MED9; SPTSSA; ACADM; ARNTL; NCALD; CHD9; JUN; PPARGC1A; GNAI3; CHKA; PCTP; PIK3R3; PIK3R1; PNPLA4; ADCY3; TXNRD1; PLCB1; PRKCB; PRKAB2; PIKFYVE; SGPL1; CHUK; GPAM; PPARA; RPS6KA3; RPS6KA5; RPS6KA6; UBE2N; MED13L; CYP8B1; MEF2C; MEF2A; INPP5K; ELOVL2; MSMO1; AGPS; CLOCK; INSIG1; VAPA; MAPK14; MAPK10; INPP4A; FABP1; GABRA5; GABRA4; GABRA1; HPGD; GNAI1; NCOA1; TBK1; PELI2; ARF3; MAPK3; MAPK1; GABRG2; SRD5A3; SRD5A2; MAPK9; RAB14; GPCPD1; PLPP3; THRAP3; GNB1; PDSS2; PDSS1; PRKAA2; MTMR7; HACD2; FADS1; GABRB2; EP300; STARD3NL; YAP1; KCNJ2; GABRB3; TBL1XR1; ARNT; MTF1; NFYA; MTMR2; CTGF; SREBF1;*	*CLTC; SOAT1; PCSK9*

Primary categories	B95.8 vs Ctrl	M81 vs Ctrl	M81 vs B95.8
	(#) Genes	(#) Genes	(#) Genes
	MBTPS2; CAMKK1; GPX4; GNAL; GPD1L; NRF1; ACSM3; MSMO1; SOAT1; LCLAT1; CROT; INSIG1; VAPB; VAPA; SC5D; GNG12; ME1; RAF1; CDIPT; NCOA2; NCOA3; NCOA1; NCOA6; SEC23A; PELI2; ARF3; ARF1; CHRNB2; FHL2; SRD5A1; CYP7A1; PLA2G3; EPB41L1; HMGCS1; ACACA; THRAP3; ACACB; MGLL; GRIA1; RXRA; ABCD1; LDLR; PLA2G2D; MCAT; UBE2V1; AKR1B1; GABRB3; SLC27A1; YAP1; KCNJ3; KCNJ2; TBL1XR1; KCNJ5; CDS1; CDS2; ALB; NFYA; ADCY9; MTMR1; AGPAT1; ABCA1; MAP2K7; CHPT1; PRKD1; PRKD2; PRKD3; OCRL; SPHK2; HMGB1; MAP2K4; BIRC3; BIRC2; MAP2K3; LTA4H; MAP2K1; GPD1; MAP2K6; SGMS1; GPD2; TEAD3; LIPE; TEAD1; ACER2; ELOVL6; CDK19; LPIN2; LPIN1; CBR4; NPAS2; ESYT2; ESYT3; HSD17B8; TMEM55B; SYNJ1; SYNJ2; HSD17B2; HSD17B4; SLC25A1; MID1IP1; CPT1A; SLC44A1; ALDH3B1; ESRRA; SLC44A5; ACER3; KPNB1; PIP5K1C; CAMK2B; STS; PLA2G4C	*PIP4K2C; PRKACB; PRKD1; PRKD3; HEXB; SCP2; BIRC2; MAP2K1; GPD2; GRIA2; TEAD1; ELOVL6; CDK19; CERS6; HADHB; NEFL; ESYT2; AP2B1; MED13; MED14; SP1; SYNJ2; MED18; HSD11B1; SLC44A1; KCNJ15; PRKCA; KPNB1; STS; MED21; PPP1CB; ARSB; TAB3; BTRC; CEPT1; MYO6; ARSJ; DDHD1; FAR1; ELOVL5; GNG12; ATF2*	
Programmed Cell Death	*(113)*	*(40)*	*(4)*
	BCL2L1; PMAIP1; ADD1; DYNLL2; PSMF1; AKT1; PKP1; DYNLL1; GSN; PPP3R1; DCC; FADD; CDH1; FNTA; ACIN1; DFFB; FASLG; NMT1; OPA1; DBNL; BAK1; SFN; UBA52; PSMC6; KPNA1; UBC; APC; PAK2; TNFSF10; CLSPN; VIM; BMF; PSMC5; STK26; STK24;	*PPP3R1; DYNLL2; PSMD5; PSMF1; PSMD1; BIRC2; PSMD10; XIAP; TP63; BCL2L11; FAS; YWHAQ; DCC; PSMA2; PSMA4; DSG3; YWHAG; APAF1; MAPT; APC; PSME4; KPNB1; PSME3; APPL1; SATB1; OPA1; PRKCQ; KPNA1; TJP2; CASP7; TJP1;*	*APPL1; XIAP; DCC; CYCS*

Primary categories	B95.8 vs Ctrl	M81 vs Ctrl	M81 vs B95.8
	(#) Genes	(#) Genes	(#) Genes
	BMX; ARHGAP10; UNC5B; UNC5A; DSP; PSME3; PLEC; LMNB1; TP53; PSMD14; PSMD11; PSMD10; PSMD13; PSMD12; GAS2; TFDP2; TFDP1; TNFRSF10B; XIAP; TP63; TICAM2; PSMA2; CFLAR; PSMA4; PSMA5; DSG2; DSG3; MAPK8; PSMD7; APAF1; H1F0; TLR4; APPL1; PSMD3; SATB1; OCLN; TJP2; TJP1; TP73; PSMB8; PSMB5; PTK2; PSMD9; PSMD8; PSMD5; HMGB1; PSMD1; BIRC2; CYCS; CTNNB1; BBC3; YWHAZ; BCL2L11; FAS; YWHAQ; YWHAH; YWHAB; PSMC2; YWHAG; DAPK1; YWHAE; PSME4; KPNB1; PRKCD; PSME1; TP53BP2; PRKCQ; LMNA; CASP6; CASP7; CASP3; E2F1; ROCK1; CASP8; CASP9; MAPT; DNM1L; BCL2	*CASP3; ROCK1; PSMD12; TLR4; DNM1L; GAS2; TFDP2; TFDP1; BCL2*	
Signal Transduction	*(906)*	*(353)*	*(61)*
	BMPR1B; MOV10; RYK; CRKL; BUB1B; PRKAG2; PCSK6; IL6ST; BRK1; EEF2K; DLG4; CDC73; PIK3CA; PIK3CB; WNT16; PIK3CD; MIB1; RNF111; CLASP1; GRIN1; IRAK1; ARHGEF11; ARHGEF12; STK11; DIAPH1; DIAPH2; ARHGEF17; CAMK2D; SP1; CAMK2G; CAMK2A; CAMK2B; STK3; STK4; DEPDC1B; PCSK5; TBL1XR1; ADRA1A; ADRA1B; ADRA1D; CCND1; GRB10; CCND3; AKAP9; USP34; PTEN; ARHGAP15; ARHGAP12; MAG; WNT2; KLC1; KLC2; ARHGAP18; ARHGAP19; NSL1; ATP6V1D; RASGRP1; ATP6V1A; RASGRP4; ATP2A3;	*RALB; NCBP1; PPP3R1; PRKAG3; PRKAG2; USP9X; IL6ST; RAPGEF4; POGLUT1; RAPGEF1; RAPGEF2; CCNT2; KIF2A; CAV1; EP300; CALM1; FMNL2; PIK3CA; SOX6; PIK3CD; MIB1; GOPC; RNF111; MAPRE1; CUL1; CUL3; ARHGEF11; IGF1R; SHC1; ERBB4; SP1; CREB1; FLRT2; STK4; CXCL12; ADRA1A; RSPO1; ADCY3; PTPN12; MYLK; TCF7L2; PTEN; ARHGAP12; CSN2; SEL1L; TNKS; RASGRP3; ATP6V1A; GNAO1; CDKN1A; ATP6V1H; HIF1A; FRS2; FAM13B; CRK; RICTOR; ARHGAP1; SMURF1; SMURF2; PPM1A; SOX13; JUN; H3F3A;*	*RASGRP3; HDAC2; ACVR2A; PSMD5; SMAD7; PRKAA2; IL6ST; ATP6V1C2; TNRC6B; CLTC; CRK; RICTOR; ACVR2B; NCAM1; FURIN; NCOA3; CCNT2; BCL2L11; PPM1A; NEFL; NTRK2; FGFR3; MAPK2; MAPK3; MAPK1; MAPK4; MAPK5; CAV1; CUL3; RRAGD; IGF1R; IGF1; KIDINS220; MTMR4; OPRM1; SOCS6; MTOR; PRKAR1A; FGF9; FGF8; FGF7; FGFR4; FGFR5; FGF2; KRAS; ADCY7; PPP1CB; ADCY1; PPP2CB; ADCY8; ADCY9; CBL; MEF2C; ITGAV; BMPR2; EREG; PRKACB; PPP3CA; PIP4K2B; ATF1; RALB*

Primary categories	B95.8 vs Ctrl	M81 vs Ctrl	M81 vs B95.8
	(#) Genes	(#) Genes	(#) Genes
	CDKN1B; ITGA2; ITGA3; ATP6V1H; RIT1; UBE2D1; PDGFD; SERPINE1; XPO1; ARHGAP11A; BUB3; SOX13; SOX17; GRAP2; JAK2; JAK3; NF1; JAK1; KSR1; CAV1; CHUK; VEGFD; COL4A5; COL4A4; VEGFA; COL4A2; VEGFC; GDI2; GDI1; CBY1; RPS6KA1; RPS6KA2; RPS6KA3; RPS6KA5; ZNRF3; SPDL1; PSMD14; NOG; PSMD11; PSMD10; CBL; PSMD12; PRDM4; FRAT1; FRAT2; HES1; MYH10; SCAI; SMAD9; RALBP1; TLE3; PAG1; TLE1; SMAD7; SRGAP1; RAP1B; SRGAP3; TLE4; FGF7; XIAP; CTTN; AKAP13; ADCYAP1; NPHP4; RSPO1; SMAD2; FGF5; PIK3AP1; TSC1; NCOA2; MDK; ITPR1; KREMEN2; WNT9B; KNTC1; ARF6; F11R; PSMD1; CHRDL1; FGF23; PRKAR1A; PRKAR1B; LEF1; YAP1; IL2RA; IL2RB; WWC1; ATP6V1E1; FOXO1; ZFYVE9; CXXC4; CDC42; NCKAP1L; PSMD9; PSMD8; ITCH; PSMD5; PSMD7; EGFR; IGF1R; PSMD3; CTNNB1; WASF2; PLCG1; LCK; NGFR; NDE1; MOB1B; PRC1; H3F3A; PGF; EPS15; ADCYAP1R1; VRK3; PDE1C; PDE1B; PDE1A; NUP98; OPHN1; ITPR2; SKA1; PEA15; CLIP1; SKA2; YES1; JAG2; PSMC2; JAG1; PSMC5; PSMC6; STAT5B; ITGB1; ITGB3; NOS3; RTN4; HGS; ABR; AGO4; HGF; NRP1; NRP2; E2F5; E2F4; TBL1X; NCK1; NCK2; ACTR3; BTRC; IER3; CILP; MAP3K11; RALA; CTNNBIP1; ARHGEF18;	EREG; PLCB1; PRKAB2; PRKAB1; CHUK; COL4A4; VEGFA; RNF41; COL4A1; PTPN3; PTPN2; PTPN1; RPS6KA3; CSNK1A1; ZNRF3; ESR1; ERBIN; MAPK25; PSMD10; CBL; PSMD12; COL4A3; TFDP2; TFDP1; PPP2R5C; USP8; SMAD9; SMAD4; PAG1; SMAD7; SRGAP1; SMAD1; SRGAP3; SRGAP2; UBE2D3; FGF7; XIAP; RHOT1; ADCYAP1; PIK3AP1; TSC1; STAT3; STAT1; NEDD4; PDE3B; PSMA2; HBEGF; PSMA4; FGD4; SRC; LEPR; WIPF2; UCHL5; LYN; MEMO1; YAP1; SFRP1; TBL1XR1; MAP2K1; ADCY9; IL3; CXXC4; ITGAV; PDE4D; ITCH; PSMD5; BIRC5; CIT; PSMD1; ARHGEF12; FOXO3; BMPR1A; FOXO1; WASL; MOB1B; PRC1; SPTBN1; DIAPH1; DIAPH2; ECT2; NEFL; STAM2; OPHN1; MAPKAP1; PEA15; SKA2; NRG1; NRG2; NET1; RTN4; PKN2; KIF18A; ABR; RHOBTB1; HGF; NRP1; NRP2; E2F5; PPP1CB; CASP3; AGO1; BTRC; TIA1; CILP; PPP3CA; COL6A3; PSMF1; SPRED1; SPRED2; IKBKB; AKT2; TNRC6C; TNRC6B; TNRC6A; RASAL2; CENPU; GNAI3; ITSN1; ARHGEF3; NTRK2; FSTL1; ARHGAP24; ARHGAP29; ARHGAP28; PIP4K2C; CENPO; CENPL; DAAM1; FGG; RET; KITLG; SMAD3; CENPQ; KRAS; CAB39L; SOCS6; BMP2; SOCS3; PPP2CB; GSK3B; GRB2; GFRA1; TIAL1; CCNC;	

Primary categories	B95.8 vs Ctrl	M81 vs Ctrl	M81 vs B95.8
	(#) Genes	(#) Genes	(#) Genes
	ATP6V0E1; ATP6V0E2; WNT3A; LIN7B; AHCTF1; MCF2; SPTB; SPRED1; SAV1; IKBKB; PTK2B; TNRC6C; TNRC6B; TNRC6A; RASAL1; RASAL2; GDNF; PEBP1; ARHGEF9; ARHGEF6; ARHGEF7; ARHGEF4; ARHGEF5; ARHGEF2; ARHGEF3; ARHGEF1; CDH1; RELA; MTMR4; CDH5; PIP4K2C; PIP4K2B; CENPO; CENPN; CENPM; CENPL; NCKIPSD; RSPO2; DAAM1; ATP1B4	*APC; PAK1; AKAP13; STARD13; TRIM33; KHDRBS1; KHDRBS2; MUC20; WIF1; RBBP5; CLTC; MIS12; STAM; AP2B1; CLASP2; PREX1; ATP6V1C1; CHD8; THBS2; THBS1; PPP1R12A; PIK3R1; BMPR2; NLK; TGFBR2; BUB3; RHOQ; ARPC5; RAG1; EIF4E; ZWILCH; ITPR1; FGF2; GALNT3; VAV3; MEF2C; MEF2A; WNT5A; IL17RD; ACTR3; ACTR2; AGO2; PDGFRA; HDAC2; LGR4; HDAC9; BMPR1B*	
Transport of small molecules	*(137)*	*(16)*	*(10)*
	SLC6A3; SLC6A2; FXYD2; FXYD3; SLC15A2; ATP2C1; SLC30A3; SLC6A6; CALM1; NDC1; SLC12A5; SLC34A2; SLC12A1; SLC12A2; NUP214; NUP210; SLCO3A1; NUP98; CAMK2D; CAMK2G; ATP1B2; CAMK2A; CAMK2B; ATP1B1; RAE1; ATP2B1; SLC15A3; ATP2B3; ATP2B2; ATP2B4; SLC15A4; SLC2A8; SLC2A4; SLC2A2; SLC2A1; ATP8B2; ATP8B1; RUNX1; SLC4A2; POM121C; SLC17A7; ATP2A2; ATP2A3; SLC7A6; SLC7A7; SLC7A5; G6PC3; G6PC2; ATP7A; SLC11A2; SLC9A1; SLC11A1; RHAG; SLC35A2; SLC35A3; HK1; POM121; GCK; SLC26A1; SLC26A2; SLC26A4; SLC26A7; SLC26A9; SLC39A2; SLC24A1; AHCYL2; G6PC; SLC8A2; NUP153; SLC22A5; SLC35B2; SLC22A2; SLC22A3; SLC18A2; NUP133; SLC32A1; SLC4A4; SLC39A1; NUP62; SLC7A8; SLC8A1; SLC6A12; SLC8A3; BSG; RANBP2; SRI; TPR; ATP12A; SLCO1A2;	*SLC4A10; SLC6A6; SLC7A6; SLC24A1; SLC4A4; SLC26A7; SLC15A4; SLC20A1; CALM1; SLC7A11; SLC12A5; SLC12A1; SLC12A2; SLC1A1; SLC1A2; SLC1A3*	*PCSK9; ADCY1; PRKAR1A; SOAT1; ADCY7; CLTC; PRKACB; MYO5B; FURIN; VLDLR*

Primary categories	B95.8 vs Ctrl	M81 vs Ctrl	M81 vs B95.8
	(#) Genes	(#) Genes	(#) Genes
	SLC27A4; CP; SLC27A1; NUP50; NUP54; NUP58; ALB; SLC17A1; ATP1A4; ATP1A3; ATP1A2; ATP1A1; PLN; SLC4A10; SLC33A1; SLC5A1; ATP11B; SLC5A6; SLC5A7; ATP13A2; PDZD11; NUP43; NUP188; HK2; SLC44A1; SLC44A2; ATP8A1; SLC44A5; NUP160; ATP10D; ATP1B3; SLN; HEPH; SEH1L; SLC20A2; SLC20A1; SLC40A1; ATP4B; SLC16A1; SLC16A3; NUP35; SLC7A11; SLC16A7; SLC1A5; SLC1A7; SLC1A1; SLC1A2; SLC1A3		
Vesicle-mediated transport	*(391)*	*(120)*	
	HSPA8; RIC1; SBF1; SBF2; SEC23IP; KIAA0319; GAPVD1; RAB3IP; SYS1; CTAGE5; GABARAP; NAPA; LRP2; PRKAG2; C2CD5; PIK3C2A; ALS2CL; TRAPPC8; COL7A1; SNAP23; TRAPPC1; TRAPPC3; SNAP29; KLC1; KLC2; TSG101; RAB6A; PUM1; DENND4A; DENND4B; DENND4C; HIP1R; APOB; TXNDC5; TBC1D20; TBC1D25; TBC1D24; MAP1LC3B; VTA1; BLOC1S6; CUX1; EPS15L1; GDI2; GDI1; SH3D19; DNAJC6; CBL; SGIP1; GOSR2; GOSR1; DENND5B; MIA3; DCTN3; IGF2R; DCTN6; DCTN4; TSC1; NEDD8; ARF1; ARF6; ARF4; ANKRD28; SH3GL2; LDLR; SAR1B; RAB33B; F8; NAA35; NAA30; LDLRAP1; CTTN; CHMP4B; CHMP4C; EGFR; AP1S1; AP1S2; AP1S3; BIN1; EPS15; VPS25; DYNC1I1; DYNC1I2; KIF4A; ARFIP2; HGS; TRIP10; TRIP11; PLA2G4A; COPS7B; COPS7A; EXOC2; RAB21;	*SCFD1; RAB3IP; PRKAG3; PRKAG2; FCHO2; AKT2; SBF2; SEC23IP; DAB2; KIF2A; TMED10; GAPVD1; CALM1; ITSN1; DYNLL2; CTAGE5; RALGAPA2; TRAPPC8; TMF1; CTSC; CHM; SEC24D; PIK3C2A; SEC24A; RAB27A; SCARB2; ARCN1; GRB2; MYO5A; RAB6A; COPS4; PUM1; ARFGAP2; CLTC; DENND4C; RAB3GAP2; STAM; AP2B1; FNBP1L; TGFA; VPS37A; CCZ1B; TSC1; VTA1; TMED7; PRKAB2; PRKAB1; KIF18A; RHOQ; ARPC5; EPN2; DENND5B; MIA3; KIF3A; AP1G1; AP4E1; CBL; WNT5A; GOSR1; ACTR3; ACTR2; AGFG1; DYNC1LI2; STXBP3; CHMP3; IGF2R; CHMP7; DENND6A; RAB8B; BET1; FZD4; TMED2; REPS2; RAB18; TRAPPC13; RAB10; ANKRD28; RAB14; SRC; AP1S3; RAB1A; RABGEF1; TJP1; RAB33B; NAA30; GJC1; STX16; TBC1D15; CD55; CD59;*	

Primary categories	B95.8 vs Ctrl	M81 vs Ctrl	M81 vs B95.8
	(#) Genes	(#) Genes	(#) Genes
	EXOC6; EXOC7; EXOC4; EXOC5; TFG; GOLIM4; ACBD3; ANK1; ANK2; ANK3; SPTB; HPS4; DAB2; COPG2; RAB5A; RAB5C; RAB5B; SYNJ2; CHM; SEC24D; RINT1; SEC24A; SEC24C; SEC24B; PAFAH1B1; PAFAH1B2; SYT9; SLC2A8; SCARB2; SYT2; SLC2A4; SFN; UBA52; COPS8; COPS2; SNX9; COPS4; COPS5; ARFGAP2; ARFGAP3; ARFGAP1; STAM; AP2B1; COPZ1; MADD; RAB11A; RAB11B; GCC2; GCC1; TMED7; FZD4; GALNT1; GALNT2; GJA1; RGP1; VPS53; VPS52; RALA; VPS54; REPS1; STXBP3; MCFD2; AREG; BET1; RACGAP1; RAB18; TRAPPC11; TRAPPC10; TRAPPC13; RAB10; RAB13; RAB12; RAB14; RAB1A; RAB1B; YKT6; TJP1; PIP5K1C; STX5; STX6; DCTN5; TRAPPC6B; EXOC8; RABGAP1; PRKAA2; MYO6; AP1M1; HIP1; YWHAZ; CNIH3; CNIH2; GABARAPL2; RALGAPB; YWHAQ; DVL2; YWHAH; TOR1A; YWHAB; AP1B1; YWHAG; YWHAE; COPA; RAC1; CHRM2; LMAN1; AP2A2; CYTH1; CYTH3; VAMP3; VAMP2; VAMP4; VAMP7; VAMP8; RHOBTB3; VTI1A; MON1A; DYNLL1; DYNLL2; FCHO2; FCHO1; KIF2A; TMED10; CALM1; PLA2G6; CLINT1; CTSC; KIF5A; ARCN1; RAB43; MYO5A; GBF1; CLVS2; APP; GORASP1; GRIA1; KIF26A; SEC16A; SEC16B; GJC1; FNBP1L; AP2S1; VPS37C; VPS37B; VPS37A; TFRC;	*PRKAA2; WASL; AP1S2; SEC16A; SPTBN1; CNIH1; STX6; RAB30; RALGAPB; STAM2; YWHAQ; TGOLN2; SYNJ1; SYNJ2; HIP1R; YWHAG; ALS2; AAK1; LMAN1; PPP6R3; LMAN3; DENND1B; COPS7B; EXOC2; VAMP4; EXOC8; MYO6; ACBD3; DYNC1I1; VTI1A*	

Primary categories	B95.8 vs Ctrl	M81 vs Ctrl	M81 vs B95.8
	(#) Genes	(#) Genes	(#) Genes
	PPP6C; SURF4; PRKAB2; PRKAB1; EPN1; EPN2; CCZ1B; AGFG1; MYO1C; CD4; ARRB2; ARRB1; CHMP3; CHMP7; CHMP5; DENND6A; VPS45		

Supplementary Table S8 – List of Gene IDs cited on main text. Predicted genes were from differentially expressed miRNAs of transfected immortalized nasopharyngeal cells with LMP1 variant from EBV strain B95.8 or M81.

Gene name	Gene ID	Gene name	Gene ID
NCBP1	4686	*MCM9*	254394
NUP43	348995	*RB1*	5925
NUP58	9818	*GABPB1*	2553
POM121	9883	*PRKAA2*	5563
RANBP2	903	*PPM1A*	5494
SOS1	6654	*FZD5*	7855
CDC7	851545	*CAV1*	857
ORC4	5000	*FOXA2*	3170
MCM10	55388	*HNF4A*	3172

References

1. Gao, F.; Zhao, Z.-L.; Zhao, W.-T.; Fan, Q.-R.; Wang, S.-C.; Li, J.; Zhang, Y.-Q.; Shi, J.-W.; Lin, X.-L.; Yang, S.; et al. MiR-9 Modulates the Expression of Interferon-Regulated Genes and MHC Class I Molecules in Human Nasopharyngeal Carcinoma Cells. *Biochem. Biophys. Res. Commun.* **2013**, *431*, 610–616, doi:10.1016/j.bbrc.2012.12.097.
2. Lu, J.; Xu, X.; Liu, X.; Peng, Y.; Zhang, B.; Wang, L.; Luo, H.; Peng, X.; Li, G.; Tian, W.; et al. Predictive Value of MiR-9 as a Potential Biomarker for Nasopharyngeal Carcinoma Metastasis. *Br. J. Cancer* **2014**, *110*, 392–398, doi:10.1038/bjc.2013.751.
3. Bandi, N.; Zbinden, S.; Gugger, M.; Arnold, M.; Kocher, V.; Hasan, L.; Kappeler, A.; Brunner, T.; Vassella, E. MiR-15a and MiR-16 Are Implicated in Cell Cycle Regulation in a Rb-Dependent Manner and Are Frequently Deleted or down-Regulated in Non-Small Cell Lung Cancer. *Cancer Res.* **2009**, *69*, 5553–5559, doi:10.1158/0008-5472.CAN-08-4277.
4. Zhu, P.; Zhang, J.; Zhu, J.; Shi, J.; Zhu, Q.; Gao, Y. MiR-429 Induces Gastric Carcinoma Cell Apoptosis Through Bcl-2. *Cell. Physiol. Biochem. Int. J. Exp. Cell. Physiol. Biochem. Pharmacol.* **2015**, *37*, 1572–1580, doi:10.1159/000438524.
5. Zhang, C.; Fang, X.; Li, W.; Shi, Q.; Wu, L.; Chen, X.; Huang, Z.; Wu, P.; Wang, Z.; Liao, Z. Influence of Recombinant Lentiviral Vector Encoding MiR-15a/16-1 in Biological Features of Human Nasopharyngeal Carcinoma CNE-2Z Cells. *Cancer Biother. Radiopharm.* **2014**, *29*, 422–427, doi:10.1089/cbr.2013.1596.

6. Cimmino, A.; Calin, G.A.; Fabbri, M.; Iorio, M.V.; Ferracin, M.; Shimizu, M.; Wojcik, S.E.; Aqeilan, R.I.; Zupo, S.; Dono, M.; et al. MiR-15 and MiR-16 Induce Apoptosis by Targeting BCL2. *Proc. Natl. Acad. Sci. U. S. A.* **2005**, *102*, 13944–13949, doi:10.1073/pnas.0506654102.
7. He, Q.; Ren, X.; Chen, J.; Li, Y.; Tang, X.; Wen, X.; Yang, X.; Zhang, J.; Wang, Y.; Ma, J.; et al. MiR-16 Targets Fibroblast Growth Factor 2 to Inhibit NPC Cell Proliferation and Invasion via PI3K/AKT and MAPK Signaling Pathways. *Oncotarget* **2015**, doi:10.18632/oncotarget.6504.
8. Li, Y.-Q.; Lu, J.-H.; Bao, X.-M.; Wang, X.-F.; Wu, J.-H.; Hong, W.-Q. MiR-24 Functions as a Tumor Suppressor in Nasopharyngeal Carcinoma through Targeting FSCN1. *J. Exp. Clin. Cancer Res. CR* **2015**, *34*, 130, doi:10.1186/s13046-015-0242-6.
9. Wang, S.; Zhang, R.; Claret, F.X.; Yang, H. Involvement of MicroRNA-24 and DNA Methylation in Resistance of Nasopharyngeal Carcinoma to Ionizing Radiation. *Mol. Cancer Ther.* **2014**, *13*, 3163–3174, doi:10.1158/1535-7163.MCT-14-0317.
10. Wu, J.-B.; Shen, L.; Qiu, L.; Duan, Q.-W.; Luo, Z.-G.; Dong, X.-X. Reversal Effect of GnT-V on the Radioresistance of Human Nasopharyngeal Carcinoma Cells by Alteration B1, 6-GlcNAc Branched N-Glycans. *Int. J. Clin. Exp. Pathol.* **2015**, *8*, 9901–9911.
11. Xu, L.; Chen, Z.; Xue, F.; Chen, W.; Ma, R.; Cheng, S.; Cui, P. MicroRNA-24 Inhibits Growth, Induces Apoptosis, and Reverses Radioresistance in Laryngeal Squamous Cell Carcinoma by Targeting X-Linked Inhibitor of Apoptosis Protein. *Cancer Cell Int.* **2015**, *15*, 61, doi:10.1186/s12935-015-0217-x.
12. Chen, J.; Yang, R.; Zhang, W.; Wang, Y. Candidate Pathways and Genes for Nasopharyngeal Carcinoma Based on Bioinformatics Study. *Int. J. Clin. Exp. Pathol.* **2015**, *8*, 2026.
13. Li, J.; Kong, X.; Zhang, J.; Luo, Q.; Li, X.; Fang, L. MiRNA-26b Inhibits Proliferation by Targeting PTGS2 in Breast Cancer. *Cancer Cell Int.* **2013**, *13*, 7, doi:10.1186/1475-2867-13-7.
14. Li, J.; Kong, X.; Zhang, J.; Luo, Q.; Li, X.; Fang, L. Correction: MiRNA-26b Inhibits Proliferation by Targeting PTGS2 in Breast Cancer. *Cancer Cell Int.* **2013**, *13*, 17, doi:10.1186/1475-2867-13-17.
15. Liu, N.; Tang, L.-L.; Sun, Y.; Cui, R.-X.; Wang, H.-Y.; Huang, B.-J.; He, Q.-M.; Jiang, W.; Ma, J. MiR-29c Suppresses Invasion and Metastasis by Targeting TIAM1 in Nasopharyngeal Carcinoma. *Cancer Lett.* **2013**, *329*, 181–188, doi:10.1016/j.canlet.2012.10.032.
16. Zeng, X.; Xiang, J.; Wu, M.; Xiong, W.; Tang, H.; Deng, M.; Li, X.; Liao, Q.; Su, B.; Luo, Z.; et al. Circulating MiR-17, MiR-20a, MiR-29c, and MiR-223 Combined as Non-Invasive Biomarkers in Nasopharyngeal Carcinoma. *PloS One* **2012**, *7*, e46367, doi:10.1371/journal.pone.0046367.
17. Baraniskin, A.; Birkenkamp-Demtroder, K.; Maghnouj, A.; Zöllner, H.; Munding, J.; Klein-Scory, S.; Reinacher-Schick, A.; Schwarte-Waldhoff, I.; Schmiegel, W.; Hahn, S.A. MiR-30a-5p Suppresses Tumor Growth in Colon Carcinoma by Targeting DTL. *Carcinogenesis* **2012**, *33*, 732–739, doi:10.1093/carcin/bgs020.
18. Wang, H.-Y.; Li, Y.-Y.; Fu, S.; Wang, X.-P.; Huang, M.-Y.; Zhang, X.; Shao, Q.; Deng, L.; Zeng, M.-S.; Zeng, Y.-X.; et al. MicroRNA-30a Promotes Invasiveness and Metastasis in Vitro and in Vivo through Epithelial-Mesenchymal Transition and Results in Poor Survival of Nasopharyngeal Carcinoma Patients. *Exp. Biol. Med. Maywood NJ* **2014**, *239*, 891–898, doi:10.1177/1535370214532758.
19. Zhu, H.; Wu, H.; Liu, X.; Li, B.; Chen, Y.; Ren, X.; Liu, C.-G.; Yang, J.-M. Regulation of Autophagy by a Beclin 1-Targeted MicroRNA, MiR-30a, in Cancer Cells. *Autophagy* **2009**, *5*, 816–823.
20. Adams, B.D.; Parsons, C.; Slack, F.J. The Tumor-Suppressive and Potential Therapeutic Functions of MiR-34a in Epithelial Carcinomas. *Expert Opin. Ther. Targets* **2015**, 1–17, doi:10.1517/14728222.2016.1114102.
21. Long, Z.; Wang, B.; Tao, D.; Huang, Y.; Tao, Z. Hypofractionated Radiotherapy Induces MiR-34a Expression and Enhances Apoptosis in Human Nasopharyngeal Carcinoma Cells. *Int. J. Mol. Med.* **2014**, *34*, 1388–1394, doi:10.3892/ijmm.2014.1937.
22. Wang, L.; Yu, J.; Xu, J.; Zheng, C.; Li, X.; Du, J. The Analysis of MicroRNA-34 Family Expression in Human Cancer Studies Comparing Cancer Tissues with Corresponding Pericarcinous Tissues. *Gene* **2015**, *554*, 1–8, doi:10.1016/j.gene.2014.10.032.

23. Li, Y.-Q.; Ren, X.-Y.; He, Q.-M.; Xu, Y.-F.; Tang, X.-R.; Sun, Y.; Zeng, M.-S.; Kang, T.-B.; Liu, N.; Ma, J. MiR-34c Suppresses Tumor Growth and Metastasis in Nasopharyngeal Carcinoma by Targeting MET. *Cell Death Dis.* **2015**, *6*, e1618, doi:10.1038/cddis.2014.582.

24. Feng, B.; Wang, R.; Chen, L.-B. MiR-100 Resensitizes Docetaxel-Resistant Human Lung Adenocarcinoma Cells (SPC-A1) to Docetaxel by Targeting Plk1. *Cancer Lett.* **2012**, *317*, 184–191, doi:10.1016/j.canlet.2011.11.024.

25. Jiang, Q.; He, M.; Guan, S.; Ma, M.; Wu, H.; Yu, Z.; Jiang, L.; Wang, Y.; Zong, X.; Jin, F.; et al. MicroRNA-100 Suppresses the Migration and Invasion of Breast Cancer Cells by Targeting FZD-8 and Inhibiting Wnt/β-Catenin Signaling Pathway. *Tumour Biol. J. Int. Soc. Oncodevelopmental Biol. Med.* **2015**, doi:10.1007/s13277-015-4342-x.

26. Shi, W.; Alajez, N.M.; Bastianutto, C.; Hui, A.B.Y.; Mocanu, J.D.; Ito, E.; Busson, P.; Lo, K.-W.; Ng, R.; Waldron, J.; et al. Significance of Plk1 Regulation by MiR-100 in Human Nasopharyngeal Cancer. *Int. J. Cancer J. Int. Cancer* **2010**, *126*, 2036–2048, doi:10.1002/ijc.24880.

27. Peng, X.H.; Huang, H.R.; Lu, J.; Liu, X.; Zhao, F.P.; Zhang, B.; Lin, S.X.; Wang, L.; Chen, H.H.; Xu, X.; et al. MiR-124 Suppresses Tumor Growth and Metastasis by Targeting Foxq1 in Nasopharyngeal Carcinoma. *Mol Cancer* **2014**, *13*, 186.

28. Chen, J.J.; Liu, S.X.; Chen, M.Z.; Zhao, Z.Y. Has-miR-125a and 125b Are Induced by Treatment with Cisplatin in Nasopharyngeal Carcinoma and Inhibit Apoptosis in a P53-dependent Manner by Targeting P53 MRNA. *Mol. Med. Rep.* **2015**, *12*, 3569–3574, doi:10.3892/mmr.2015.3863.

29. Zhang, J.; Na, S.; Liu, C.; Pan, S.; Cai, J.; Qiu, J. MicroRNA-125b Suppresses the Epithelial-Mesenchymal Transition and Cell Invasion by Targeting ITGA9 in Melanoma. *Tumour Biol. J. Int. Soc. Oncodevelopmental Biol. Med.* **2015**, doi:10.1007/s13277-015-4409-8.

30. Golubovskaya, V.M.; Sumbler, B.; Ho, B.; Yemma, M.; Cance, W.G. MiR-138 and MiR-135 Directly Target Focal Adhesion Kinase, Inhibit Cell Invasion, and Increase Sensitivity to Chemotherapy in Cancer Cells. *Anticancer Agents Med. Chem.* **2014**, *14*, 18–28.

31. Tang, J.-F.; Yu, Z.-H.; Liu, T.; Lin, Z.-Y.; Wang, Y.-H.; Yang, L.-W.; He, H.-J.; Cao, J.; Huang, H.-L.; Liu, G. Five MiRNAs as Novel Diagnostic Biomarker Candidates for Primary Nasopharyngeal Carcinoma. *Asian Pac. J. Cancer Prev.* **2014**, *15*, 7575–7581, doi:10.7314/APJCP.2014.15.18.7575.

32. Li, J.; Wang, Q.; Wen, R.; Liang, J.; Zhong, X.; Yang, W.; Su, D.; Tang, J. MiR-138 Inhibits Cell Proliferation and Reverses Epithelial-Mesenchymal Transition in Non-Small Cell Lung Cancer Cells by Targeting GIT1 and SEMA4C. *J. Cell. Mol. Med.* **2015**, *19*, 2793–2805, doi:10.1111/jcmm.12666.

33. Liu, X.; Lv, X.-B.; Wang, X.-P.; Sang, Y.; Xu, S.; Hu, K.; Wu, M.; Liang, Y.; Liu, P.; Tang, J.; et al. MiR-138 Suppressed Nasopharyngeal Carcinoma Growth and Tumorigenesis by Targeting the CCND1 Oncogene. *Cell Cycle Georget. Tex* **2012**, *11*, 2495–2506, doi:10.4161/cc.20898.

34. Dong, W.; Yao, C.; Teng, X.; Chai, J.; Yang, X.; Li, B. MiR-140-3p Suppressed Cell Growth and Invasion by Downregulating the Expression of ATP8A1 in Non-Small Cell Lung Cancer. *Tumour Biol. J. Int. Soc. Oncodevelopmental Biol. Med.* **2015**, doi:10.1007/s13277-015-3452-9.

35. Lan, H.; Chen, W.; He, G.; Yang, S. MiR-140-5p Inhibits Ovarian Cancer Growth Partially by Repression of PDGFRA. *Biomed. Pharmacother. Biomédecine Pharmacothérapie* **2015**, *75*, 117–122, doi:10.1016/j.biopha.2015.07.035.

36. Yang, H.; Fang, F.; Chang, R.; Yang, L. MicroRNA-140-5p Suppresses Tumor Growth and Metastasis by Targeting Transforming Growth Factor β Receptor 1 and Fibroblast Growth Factor 9 in Hepatocellular Carcinoma. *Hepatol. Baltim. Md* **2013**, *58*, 205–217, doi:10.1002/hep.26315.

37. Zhai, H.; Fesler, A.; Ba, Y.; Wu, S.; Ju, J. Inhibition of Colorectal Cancer Stem Cell Survival and Invasive Potential by Hsa-MiR-140-5p Mediated Suppression of Smad2 and Autophagy. *Oncotarget* **2015**, *6*, 19735–19746, doi:10.18632/oncotarget.3771.

38. Zhang, W.; Zou, C.; Pan, L.; Xu, Y.; Qi, W.; Ma, G.; Hou, Y.; Jiang, P. MicroRNA-140-5p Inhibits the Progression of Colorectal Cancer by Targeting VEGFA. *Cell. Physiol. Biochem. Int. J. Exp. Cell. Physiol. Biochem. Pharmacol.* **2015**, *37*, 1123–1133, doi:10.1159/000430237.

39. Liu, L.; Yu, X.; Guo, X.; Tian, Z.; Su, M.; Long, Y.; Huang, C.; Zhou, F.; Liu, M.; Wu, X.; et al. MiR-143 Is Downregulated in Cervical Cancer and Promotes Apoptosis and Inhibits Tumor Formation by Targeting Bcl-2. *Mol. Med. Rep.* **2012**, *5*, 753–760, doi:10.3892/mmr.2011.696.

40. Huang, H.; Sun, P.; Lei, Z.; Li, M.; Wang, Y.; Zhang, H.-T.; Liu, J. MiR-145 Inhibits Invasion and Metastasis by Directly Targeting Smad3 in Nasopharyngeal Cancer. *Tumor Biol.* **2015**, *36*, 4123–4131, doi:10.1007/s13277-015-3046-6.

41. Li, Y.-Q.; He, Q.-M.; Ren, X.-Y.; Tang, X.-R.; Xu, Y.-F.; Wen, X.; Yang, X.-J.; Ma, J.; Liu, N. MiR-145 Inhibits Metastasis by Targeting Fascin Actin-Bundling Protein 1 in Nasopharyngeal Carcinoma. *PLOS ONE* **2015**, *10*, e0122228, doi:10.1371/journal.pone.0122228.

42. Li, H.-P.; Huang, H.-Y.; Lai, Y.-R.; Huang, J.-X.; Chang, K.-P.; Hsueh, C.; Chang, Y.-S. Silencing of MiRNA-148a by Hypermethylation Activates the Integrin-Mediated Signaling Pathway in Nasopharyngeal Carcinoma. *Oncotarget* **2014**, *5*, 7610–7624, doi:10.18632/oncotarget.2282.

43. Guan, Z.; Song, B.; Liu, F.; Sun, D.; Wang, K.; Qu, H. TGF-β Induces HLA-G Expression through Inhibiting MiR-152 in Gastric Cancer Cells. *J. Biomed. Sci.* **2015**, *22*, 107, doi:10.1186/s12929-015-0177-4.

44. Huang, H.; Hu, M.; Li, P.; Lu, C.; Li, M. Mir-152 Inhibits Cell Proliferation and Colony Formation of CD133(+) Liver Cancer Stem Cells by Targeting KIT. *Tumour Biol. J. Int. Soc. Oncodevelopmental Biol. Med.* **2015**, *36*, 921–928, doi:10.1007/s13277-014-2719-x.

45. Fang, F.; Chang, R.; Yu, L.; Lei, X.; Xiao, S.; Yang, H.; Yang, L.-Y. MicroRNA-188-5p Suppresses Tumor Cell Proliferation and Metastasis by Directly Targeting FGF5 in Hepatocellular Carcinoma. *J. Hepatol.* **2015**, *63*, 874–885, doi:10.1016/j.jhep.2015.05.008.

46. Wang, L.; Liu, H. MicroRNA-188 Is Downregulated in Oral Squamous Cell Carcinoma and Inhibits Proliferation and Invasion by Targeting SIX1. *Tumour Biol. J. Int. Soc. Oncodevelopmental Biol. Med.* **2015**, doi:10.1007/s13277-015-4246-9.

47. Wu, J.; Lv, Q.; He, J.; Zhang, H.; Mei, X.; Cui, K.; Huang, N.; Xie, W.; Xu, N.; Zhang, Y. MicroRNA-188 Suppresses G1/S Transition by Targeting Multiple Cyclin/CDK Complexes. *Cell Commun. Signal. CCS* **2014**, *12*, 66, doi:10.1186/s12964-014-0066-6.

48. Kinose, Y.; Sawada, K.; Nakamura, K.; Sawada, I.; Toda, A.; Nakatsuka, E.; Hashimoto, K.; Mabuchi, S.; Takahashi, K.; Kurachi, H.; et al. The Hypoxia-Related MicroRNA MiR-199a-3p Displays Tumor Suppressor Functions in Ovarian Carcinoma. *Oncotarget* **2015**, *6*, 11342–11356, doi:10.18632/oncotarget.3604.

49. Shatseva, T.; Lee, D.Y.; Deng, Z.; Yang, B.B. MicroRNA MiR-199a-3p Regulates Cell Proliferation and Survival by Targeting Caveolin-2. *J. Cell Sci.* **2011**, *124*, 2826–2836, doi:10.1242/jcs.077529.

50. Gregory, P.A.; Bert, A.G.; Paterson, E.L.; Barry, S.C.; Tsykin, A.; Farshid, G.; Vadas, M.A.; Khew-Goodall, Y.; Goodall, G.J. The MiR-200 Family and MiR-205 Regulate Epithelial to Mesenchymal Transition by Targeting ZEB1 and SIP1. *Nat. Cell Biol.* **2008**, *10*, 593–601, doi:10.1038/ncb1722.

51. Korpal, M.; Lee, E.S.; Hu, G.; Kang, Y. The MiR-200 Family Inhibits Epithelial-Mesenchymal Transition and Cancer Cell Migration by Direct Targeting of E-Cadherin Transcriptional Repressors ZEB1 and ZEB2. *J. Biol. Chem.* **2008**, *283*, 14910–14914, doi:10.1074/jbc.C800074200.

52. Pecot, C.V.; Rupaimoole, R.; Yang, D.; Akbani, R.; Ivan, C.; Lu, C.; Wu, S.; Han, H.-D.; Shah, M.Y.; Rodriguez-Aguayo, C.; et al. Tumour Angiogenesis Regulation by the MiR-200 Family. *Nat. Commun.* **2013**, *4*, 2427–2427, doi:10.1038/ncomms3427.

53. Uhlmann, S.; Zhang, J.D.; Schwäger, A.; Mannsperger, H.; Riazalhosseini, Y.; Burmester, S.; Ward, A.; Korf, U.; Wiemann, S.; Sahin, O. MiR-200bc/429 Cluster Targets PLCgamma1 and Differentially Regulates Proliferation and EGF-Driven Invasion than MiR-200a/141 in Breast Cancer. *Oncogene* **2010**, *29*, 4297–4306, doi:10.1038/onc.2010.201.

54. Xia, H.; Ng, S.S.; Jiang, S.; Cheung, W.K.C.; Sze, J.; Bian, X.-W.; Kung, H.-F.; Lin, M.C. MiR-200a-Mediated Downregulation of ZEB2 and CTNNB1 Differentially Inhibits Nasopharyngeal Carcinoma Cell Growth, Migration and Invasion. *Biochem. Biophys. Res. Commun.* **2010**, *391*, 535–541, doi:10.1016/j.bbrc.2009.11.093.

55. Zuberi, M.; Mir, R.; Das, J.; Ahmad, I.; Javid, J.; Yadav, P.; Masroor, M.; Ahmad, S.; Ray, P.C.; Saxena, A. Expression of Serum MiR-200a, MiR-200b, and MiR-200c as Candidate Biomarkers in Epithelial Ovarian Cancer and Their Association with Clinicopathological Features. *Clin. Transl. Oncol. Off. Publ. Fed. Span. Oncol. Soc. Natl. Cancer Inst. Mex.* **2015**, *17*, 779–787, doi:10.1007/s12094-015-1303-1.

56. Zuberi, M.; Mir, R.; Das, J.; Ahmad, I.; Javid, J.; Yadav, P.; Masroor, M.; Ahmad, S.; Ray, P.C.; Saxena, A. Erratum to: Expression of Serum MiR-200a, MiR-200b and MiR-200c as Candidate Biomarkers in Epithelial Ovarian Cancer and Their Association with Clinicopathological Features. *Clin. Transl. Oncol. Off. Publ. Fed. Span. Oncol. Soc. Natl. Cancer Inst. Mex.* **2015**, *17*, 840, doi:10.1007/s12094-015-1355-2.

57. Mongroo, P.S.; Rustgi, A.K. The Role of the MiR-200 Family in Epithelial-Mesenchymal Transition. *Cancer Biol. Ther.* **2010**, *10*, 219–222, doi:10.4161/cbt.10.3.12548.

58. Wu, H.; Wang, G.; Wang, Z.; An, S.; Ye, P.; Luo, S. A Negative Feedback Loop between MiR-200b and the NF-KB Pathway via IKBKB/IKK-β in Breast Cancer Cells. *FEBS J.* **2015**, doi:10.1111/febs.13543.

59. Zhang, H.-F.; Alshareef, A.; Wu, C.; Li, S.; Jiao, J.-W.; Cao, H.-H.; Lai, R.; Xu, L.-Y.; Li, E.-M. Loss of MiR-200b Promotes Invasion via Activating the Kindlin-2/Integrin B1/AKT Pathway in Esophageal Squamous Cell Carcinoma: An E-Cadherin-Independent Mechanism. *Oncotarget* **2015**, *6*, 28949–28960, doi:10.18632/oncotarget.5027.

60. Liu, X.-G.; Zhu, W.-Y.; Huang, Y.-Y.; Ma, L.-N.; Zhou, S.-Q.; Wang, Y.-K.; Zeng, F.; Zhou, J.-H.; Zhang, Y.-K. High Expression of Serum MiR-21 and Tumor MiR-200c Associated with Poor Prognosis in Patients with Lung Cancer. *Med. Oncol. Northwood Lond. Engl.* **2012**, *29*, 618–626, doi:10.1007/s12032-011-9923-y.

61. Lo, W.-L.; Yu, C.-C.; Chiou, G.-Y.; Chen, Y.-W.; Huang, P.-I.; Chien, C.-S.; Tseng, L.-M.; Chu, P.-Y.; Lu, K.-H.; Chang, K.-W.; et al. MicroRNA-200c Attenuates Tumour Growth and Metastasis of Presumptive Head and Neck Squamous Cell Carcinoma Stem Cells. *J. Pathol.* **2011**, *223*, 482–495, doi:10.1002/path.2826.

62. Song, C.; Liu, L.-Z.; Pei, X.-Q.; Liu, X.; Yang, L.; Ye, F.; Xie, X.; Chen, J.; Tang, H.; Xie, X. MiR-200c Inhibits Breast Cancer Proliferation by Targeting KRAS. *Oncotarget* **2015**, *6*, 34968–34978, doi:10.18632/oncotarget.5198.

63. Du, Y.; Xu, Y.; Ding, L.; Yao, H.; Yu, H.; Zhou, T.; Si, J. Down-Regulation of MiR-141 in Gastric Cancer and Its Involvement in Cell Growth. *J. Gastroenterol.* **2009**, *44*, 556–561, doi:10.1007/s00535-009-0037-7.

64. Miao, Y. Hsa-MiR-141 Downregulates TM4SF1 to Inhibit Pancreatic Cancer Cell Invasion and Migration. *Int. J. Oncol.* **2013**, doi:10.3892/ijo.2013.2189.

65. Vrba, L.; Jensen, T.J.; Garbe, J.C.; Heimark, R.L.; Cress, A.E.; Dickinson, S.; Stampfer, M.R.; Futscher, B.W. Role for DNA Methylation in the Regulation of MiR-200c and MiR-141 Expression in Normal and Cancer Cells. *PloS One* **2010**, *5*, e8697, doi:10.1371/journal.pone.0008697.

66. Chen, J.; Wang, L.; Matyunina, L.V.; Hill, C.G.; McDonald, J.F. Overexpression of MiR-429 Induces Mesenchymal-to-Epithelial Transition (MET) in Metastatic Ovarian Cancer Cells. *Gynecol. Oncol.* **2011**, *121*, 200–205, doi:10.1016/j.ygyno.2010.12.339.

67. Sun, T.; Wang, C.; Xing, J.; Wu, D. MiR-429 Modulates the Expression of c-Myc in Human Gastric Carcinoma Cells. *Eur. J. Cancer Oxf. Engl. 1990* **2011**, *47*, 2552–2559, doi:10.1016/j.ejca.2011.05.021.

68. Ma, L.; Deng, X.; Wu, M.; Zhang, G.; Huang, J. Down-Regulation of MiRNA-204 by LMP-1 Enhances CDC42 Activity and Facilitates Invasion of EBV-Associated Nasopharyngeal Carcinoma Cells. *FEBS Lett.* **2014**, *588*, 1562–1570, doi:10.1016/j.febslet.2014.02.039.

69. Wang, X.; Qiu, W.; Zhang, G.; Xu, S.; Gao, Q.; Yang, Z. MicroRNA-204 Targets JAK2 in Breast Cancer and Induces Cell Apoptosis through the STAT3/BCl-2/Survivin Pathway. *Int. J. Clin. Exp. Pathol.* **2015**, *8*, 5017–5025.

70. Wu, Z.Y.; Wang, S.M.; Chen, Z.H.; Huv, S.X.; Huang, K.; Huang, B.J.; Du, J.L.; Huang, C.M.; Peng, L.; Jian, Z.X.; et al. MiR-204 Regulates HMGA2 Expression and Inhibits Cell Proliferation in

Human Thyroid Cancer. *Cancer Biomark. Sect. Dis. Markers* **2015**, *15*, 535–542, doi:10.3233/CBM-150492.

71. Yang, W.; Lan, X.; Li, D.; Li, T.; Lu, S. MiR-223 Targeting MAFB Suppresses Proliferation and Migration of Nasopharyngeal Carcinoma Cells. *BMC Cancer* **2015**, *15*, doi:10.1186/s12885-015-1464-x.

72. Alajez, N.M.; Lenarduzzi, M.; Ito, E.; Hui, A.B.Y.; Shi, W.; Bruce, J.; Yue, S.; Huang, S.H.; Xu, W.; Waldron, J.; et al. MiR-218 Suppresses Nasopharyngeal Cancer Progression through Downregulation of Survivin and the SLIT2-ROBO1 Pathway. *Cancer Res.* **2011**, *71*, 2381–2391, doi:10.1158/0008-5472.CAN-10-2754.

73. Qi, X.; Li, J.; Zhou, C.; Lv, C.; Tian, M. MicroRNA-320a Inhibits Cell Proliferation, Migration and Invasion by Targeting BMI-1 in Nasopharyngeal Carcinoma. *FEBS Lett.* **2014**, *588*, 3732–3738, doi:10.1016/j.febslet.2014.08.021.

74. Shan, Y.; Li, X.; You, B.; Shi, S.; Zhang, Q.; You, Y. MicroRNA-338 Inhibits Migration and Proliferation by Targeting Hypoxia-Induced Factor 1α in Nasopharyngeal Carcinoma. *Oncol. Rep.* **2015**, *34*, 1943–1952, doi:10.3892/or.2015.4195.

75. Chapman, B.V.; Wald, A.I.; Akhtar, P.; Munko, A.C.; Xu, J.; Gibson, S.P.; Grandis, J.R.; Ferris, R.L.; Khan, S.A. MicroRNA-363 Targets Myosin 1B to Reduce Cellular Migration in Head and Neck Cancer. *BMC Cancer* **2015**, *15*, 861, doi:10.1186/s12885-015-1888-3.

76. Ou, Y.; Zhai, D.; Wu, N.; Li, X. Downregulation of MiR-363 Increases Drug Resistance in Cisplatin-Treated HepG2 by Dysregulating Mcl-1. *Gene* **2015**, *572*, 116–122, doi:10.1016/j.gene.2015.07.002.

77. Zhang, R.; Li, Y.; Dong, X.; Peng, L.; Nie, X. MiR-363 Sensitizes Cisplatin-Induced Apoptosis Targeting in Mcl-1 in Breast Cancer. *Med. Oncol. Northwood Lond. Engl.* **2014**, *31*, 347, doi:10.1007/s12032-014-0347-3.

78. Gu, X.; Li, J.-Y.; Guo, J.; Li, P.-S.; Zhang, W.-H. Influence of MiR-451 on Drug Resistances of Paclitaxel-Resistant Breast Cancer Cell Line. *Med. Sci. Monit. Int. Med. J. Exp. Clin. Res.* **2015**, *21*, 3291–3297.

79. Liu, N.; Jiang, N.; Guo, R.; Jiang, W.; He, Q.-M.; Xu, Y.-F.; Li, Y.-Q.; Tang, L.-L.; Mao, Y.-P.; Sun, Y.; et al. MiR-451 Inhibits Cell Growth and Invasion by Targeting MIF and Is Associated with Survival in Nasopharyngeal Carcinoma. *Mol. Cancer* **2013**, *12*, 123, doi:10.1186/1476-4598-12-123.

80. Zhang, T.; Sun, Q.; Liu, T.; Chen, J.; Du, S.; Ren, C.; Liao, G.; Yuan, Y. MiR-451 Increases Radiosensitivity of Nasopharyngeal Carcinoma Cells by Targeting Ras-Related Protein 14 (RAB14). *Tumour Biol. J. Int. Soc. Oncodevelopmental Biol. Med.* **2014**, *35*, 12593–12599, doi:10.1007/s13277-014-2581-x.

81. Dadpay, M.; Zarea, M.; Rabati, R.G.; Rezakhaniha, B.; Barari, B.; Behnod, V.; Ziari, K. Upregulation of MiR-21 and Downregulation of MiR-494 May Serve as Emerging Molecular Biomarkers for Prediagnostic Samples of Subjects Who Developed Nasopharyngeal Carcinoma Associates with Lymph Node Metastasis and Poor Prognosis. *Tumour Biol. J. Int. Soc. Oncodevelopmental Biol. Med.* **2015**, doi:10.1007/s13277-015-3905-1.

82. Duan, H.-F.; Li, X.-Q.; Hu, H.-Y.; Li, Y.-C.; Cai, Z.; Mei, X.-S.; Yu, P.; Nie, L.-P.; Zhang, W.; Yu, Z.-D.; et al. Functional Elucidation of MiR-494 in the Tumorigenesis of Nasopharyngeal Carcinoma. *Tumour Biol. J. Int. Soc. Oncodevelopmental Biol. Med.* **2015**, *36*, 6679–6689, doi:10.1007/s13277-015-3356-8.

83. Nie, G.-H.; Luo, L.; Duan, H.-F.; Li, X.-Q.; Yin, M.-J.; Li, Z.; Zhang, W. GALNT7, a Target of MiR-494, Participates in the Oncogenesis of Nasopharyngeal Carcinoma. *Tumour Biol. J. Int. Soc. Oncodevelopmental Biol. Med.* **2015**, doi:10.1007/s13277-015-4281-6.

84. Wang, S.; Mo, Y.; Midorikawa, K.; Zhang, Z.; Huang, G.; Ma, N.; Zhao, W.; Hiraku, Y.; Oikawa, S.; Murata, M. The Potent Tumor Suppressor MiR-497 Inhibits Cancer Phenotypes in Nasopharyngeal Carcinoma by Targeting ANLN and HSPA4L. *Oncotarget* **2015**, *6*, 35893–35907, doi:10.18632/oncotarget.5651.

85. Zhang, Z.; Ma, J.; Luan, G.; Kang, L.; Su, Y.; He, Y.; Luan, F. MiR-506 Suppresses Tumor Proliferation and Invasion by Targeting FOXQ1 in Nasopharyngeal Carcinoma. *PLOS ONE* **2015**, *10*, e0122851, doi:10.1371/journal.pone.0122851.
86. Jin, H.; Wang, W. MicroRNA-539 Suppresses Osteosarcoma Cell Invasion and Migration in Vitro and Targeting Matrix Metallopeptidase-8. *Int. J. Clin. Exp. Pathol.* **2015**, *8*, 8075–8082.
87. Lv, L.-Y.; Wang, Y.-Z.; Zhang, Q.; Zang, H.-R.; Wang, X.-J. MiR-539 Induces Cell Cycle Arrest in Nasopharyngeal Carcinoma by Targeting Cyclin-Dependent Kinase 4. *Cell Biochem. Funct.* **2015**, *33*, 534–540, doi:10.1002/cbf.3152.
88. Mirghasemi, A.; Taheriazam, A.; Karbasy, S.H.; Torkaman, A.; Shakeri, M.; Yahaghi, E.; Mokarizadeh, A. Down-Regulation of MiR-133a and MiR-539 Are Associated with Unfavorable Prognosis in Patients Suffering from Osteosarcoma. *Cancer Cell Int.* **2015**, *15*, doi:10.1186/s12935-015-0237-6.
89. Cui, Z.; Tang, J.; Chen, J.; Wang, Z. Hsa-MiR-574-5p Negatively Regulates MACC-1 Expression to Suppress Colorectal Cancer Liver Metastasis. *Cancer Cell Int.* **2014**, *14*, 47, doi:10.1186/1475-2867-14-47.
90. Su, Y.; Ni, Z.; Wang, G.; Cui, J.; Wei, C.; Wang, J.; Yang, Q.; Xu, Y.; Li, F. Aberrant Expression of MicroRNAs in Gastric Cancer and Biological Significance of MiR-574-3p. *Int. Immunopharmacol.* **2012**, *13*, 468–475, doi:10.1016/j.intimp.2012.05.016.
91. Peng, X.; Cao, P.; He, D.; Han, S.; Zhou, J.; Tan, G.; Li, W.; Yu, F.; Yu, J.; Li, Z.; et al. MiR-634 Sensitizes Nasopharyngeal Carcinoma Cells to Paclitaxel and Inhibits Cell Growth Both in Vitro and in Vivo. *Int. J. Clin. Exp. Pathol.* **2014**, *7*, 6784–6791.
92. Liu, Z.; Long, X.; Chao, C.; Yan, C.; Wu, Q.; Hua, S.; Zhang, Y.; Wu, A.; Fang, W. Knocking down CDK4 Mediates the Elevation of Let-7c Suppressing Cell Growth in Nasopharyngeal Carcinoma. *BMC Cancer* **2014**, *14*, 274, doi:10.1186/1471-2407-14-274.
93. Nadiminty, N.; Tummala, R.; Lou, W.; Zhu, Y.; Shi, X.-B.; Zou, J.X.; Chen, H.; Zhang, J.; Chen, X.; Luo, J.; et al. MicroRNA Let-7c Is Downregulated in Prostate Cancer and Suppresses Prostate Cancer Growth. *PloS One* **2012**, *7*, e32832, doi:10.1371/journal.pone.0032832.
94. Zhao, B.; Han, H.; Chen, J.; Zhang, Z.; Li, S.; Fang, F.; Zheng, Q.; Ma, Y.; Zhang, J.; Wu, N.; et al. MicroRNA Let-7c Inhibits Migration and Invasion of Human Non-Small Cell Lung Cancer by Targeting ITGB3 and MAP4K3. *Cancer Lett.* **2014**, *342*, 43–51, doi:10.1016/j.canlet.2013.08.030.
95. Kolenda, T.; Przybyła, W.; Teresiak, A.; Mackiewicz, A.; Lamperska, K.M. The Mystery of Let-7d - a Small RNA with Great Power. *Contemp. Oncol. Poznań Pol.* **2014**, *18*, 293–301, doi:10.5114/wo.2014.44467.
96. Su, B.; Zhao, W.; Shi, B.; Zhang, Z.; Yu, X.; Xie, F.; Guo, Z.; Zhang, X.; Liu, J.; Shen, Q.; et al. Let-7d Suppresses Growth, Metastasis, and Tumor Macrophage Infiltration in Renal Cell Carcinoma by Targeting COL3A1 and CCL7. *Mol. Cancer* **2014**, *13*, 206, doi:10.1186/1476-4598-13-206.
97. Wu, K.; Yang, Y.; Zhao, J.; Zhao, S. BAG3-Mediated MiRNA Let-7g and Let-7i Inhibit Proliferation and Enhance Apoptosis of Human Esophageal Carcinoma Cells by Targeting the Drug Transporter ABCC10. *Cancer Lett.* **2016**, *371*, 125–133, doi:10.1016/j.canlet.2015.11.031.
98. Allaya, N.; Khabir, A.; Sallemi-Boudawara, T.; Sellami, N.; Daoud, J.; Ghorbel, A.; Frikha, M.; Gargouri, A.; Mokdad-Gargouri, R.; Ayadi, W. Over-Expression of MiR-10b in NPC Patients: Correlation with LMP1 and Twist1. *Tumor Biol.* **2015**, *36*, 3807–3814, doi:10.1007/s13277-014-3022-6.
99. Shan, S.W.; Fang, L.; Shatseva, T.; Rutnam, Z.J.; Yang, X.; Du, W.; Lu, W.-Y.; Xuan, J.W.; Deng, Z.; Yang, B.B. Mature MiR-17-5p and Passenger MiR-17-3p Induce Hepatocellular Carcinoma by Targeting PTEN, GalNT7 and Vimentin in Different Signal Pathways. *J. Cell Sci.* **2013**, *126*, 1517–1530, doi:10.1242/jcs.122895.
100. Wang, M.; Gu, H.; Wang, S.; Qian, H.; Zhu, W.; Zhang, L.; Zhao, C.; Tao, Y.; Xu, W. Circulating MiR-17-5p and MiR-20a: Molecular Markers for Gastric Cancer. *Mol. Med. Rep.* **2012**, *5*, 1514–1520, doi:10.3892/mmr.2012.828.
101. Chen, X.; Wang, J.; Cheng, L.; Lu, M.-P. MiR-18a Downregulates DICER1 and Promotes Proliferation and Metastasis of Nasopharyngeal Carcinoma. *Int. J. Clin. Exp. Med.* **2014**, *7*, 847.

102. Luo, Z.; Dai, Y.; Zhang, L.; Jiang, C.; Li, Z.; Yang, J.; McCarthy, J.B.; She, X.; Zhang, W.; Ma, J.; et al. MiR-18a Promotes Malignant Progression by Impairing MicroRNA Biogenesis in Nasopharyngeal Carcinoma. *Carcinogenesis* **2013**, *34*, 415–425, doi:10.1093/carcin/bgs329.

103. Huang, L.; Wang, X.; Wen, C.; Yang, X.; Song, M.; Chen, J.; Wang, C.; Zhang, B.; Wang, L.; Iwamoto, A.; et al. Hsa-MiR-19a Is Associated with Lymph Metastasis and Mediates the TNF-α Induced Epithelial-to-Mesenchymal Transition in Colorectal Cancer. *Sci. Rep.* **2015**, *5*, 13350, doi:10.1038/srep13350.

104. Xiao, W.; Gao, Z.; Duan, Y.; Yuan, W.; Ke, Y. Downregulation of MiR-19a Exhibits Inhibitory Effects on Metastatic Renal Cell Carcinoma by Targeting PIK3CA and Inactivating Notch Signaling in Vitro. *Oncol. Rep.* **2015**, *34*, 739–746, doi:10.3892/or.2015.4041.

105. Tsuchida, A.; Ohno, S.; Wu, W.; Borjigin, N.; Fujita, K.; Aoki, T.; Ueda, S.; Takanashi, M.; Kuroda, M. MiR-92 Is a Key Oncogenic Component of the MiR-17-92 Cluster in Colon Cancer. *Cancer Sci.* **2011**, *102*, 2264–2271, doi:10.1111/j.1349-7006.2011.02081.x.

106. Fonseca-Sanchéz, M.A.; Pérez-Plasencia, C.; Fernández-Retana, J.; Arechaga-Ocampo, E.; Marchat, L.A.; Rodríguez-Cuevas, S.; Bautista-Piña, V.; Arellano-Anaya, Z.E.; Flores-Pérez, A.; Diaz-Chávez, J.; et al. MicroRNA-18b Is Upregulated in Breast Cancer and Modulates Genes Involved in Cell Migration. *Oncol. Rep.* **2013**, *30*, 2399–2410, doi:10.3892/or.2013.2691.

107. Murakami, Y.; Tamori, A.; Itami, S.; Tanahashi, T.; Toyoda, H.; Tanaka, M.; Wu, W.; Brojigin, N.; Kaneoka, Y.; Maeda, A.; et al. The Expression Level of MiR-18b in Hepatocellular Carcinoma Is Associated with the Grade of Malignancy and Prognosis. *BMC Cancer* **2013**, *13*, 99, doi:10.1186/1471-2407-13-99.

108. Yu, X.; Zhen, Y.; Yang, H.; Wang, H.; Zhou, Y.; Wang, E.; Marincola, F.M.; Mai, C.; Chen, Y.; Wei, H.; et al. Loss of Connective Tissue Growth Factor as an Unfavorable Prognosis Factor Activates MiR-18b by PI3K/AKT/C-Jun and C-Myc and Promotes Cell Growth in Nasopharyngeal Carcinoma. *Cell Death Dis.* **2013**, *4*, e634, doi:10.1038/cddis.2013.153.

109. Ou, H.; Li, Y.; Kang, M. Activation of MiR-21 by STAT3 Induces Proliferation and Suppresses Apoptosis in Nasopharyngeal Carcinoma by Targeting PTEN Gene. *PLoS ONE* **2014**, *9*, e109929, doi:10.1371/journal.pone.0109929.

110. Jin, A.-H.; Wei, Z.-L. Molecular Mechanism of Increased Sensitivity of Cisplatin to Ovarian Cancer by Inhibition of MicroRNA-23a Expression. *Int. J. Clin. Exp. Med.* **2015**, *8*, 13329–13334.

111. Tian, K.; Di, R.; Wang, L. MicroRNA-23a Enhances Migration and Invasion through PTEN in Osteosarcoma. *Cancer Gene Ther.* **2015**, *22*, 351–359, doi:10.1038/cgt.2015.27.

112. Huang, T.-T.; Ping, Y.-H.; Wang, A.-M.; Ke, C.-C.; Fang, W.-L.; Huang, K.-H.; Lee, H.-C.; Chi, C.-W.; Yeh, T.-S. The Reciprocal Regulation Loop of Notch2 Pathway and MiR-23b in Controlling Gastric Carcinogenesis. *Oncotarget* **2015**, *6*, 18012–18026, doi:10.18632/oncotarget.4000.

113. Ma, G.; Dai, W.; Sang, A.; Yang, X.; Gao, C. Upregulation of MicroRNA-23a/b Promotes Tumor Progression and Confers Poor Prognosis in Patients with Gastric Cancer. *Int. J. Clin. Exp. Pathol.* **2014**, *7*, 8833–8840.

114. Peng, H.; Wang, X.; Zhang, P.; Sun, T.; Ren, X.; Xia, Z. MiR-27a Promotes Cell Proliferation and Metastasis in Renal Cell Carcinoma. *Int. J. Clin. Exp. Pathol.* **2015**, *8*, 2259–2266.

115. Wu, X.-Z.; Wang, K.-P.; Song, H.-J.; Xia, J.-H.; Jiang, Y.; Wang, Y.-L. MiR-27a-3p Promotes Esophageal Cancer Cell Proliferation via F-Box and WD Repeat Domain-Containing 7 (FBXW7) Suppression. *Int. J. Clin. Exp. Med.* **2015**, *8*, 15556–15562.

116. Zhao, Q.; Li, Y.; Tan, B.-B.; Fan, L.-Q.; Yang, P.-G.; Tian, Y. HIF-1α Induces Multidrug Resistance in Gastric Cancer Cells by Inducing MiR-27a. *PloS One* **2015**, *10*, e0132746, doi:10.1371/journal.pone.0132746.

117. Qiu, F.; Sun, R.; Deng, N.; Guo, T.; Cao, Y.; Yu, Y.; Wang, X.; Zou, B.; Zhang, S.; Jing, T.; et al. MiR-29a/b Enhances Cell Migration and Invasion in Nasopharyngeal Carcinoma Progression by Regulating SPARC and COL3A1 Gene Expression. *PloS One* **2015**, *10*, e0120969, doi:10.1371/journal.pone.0120969.

118. Cheung, C.C.; Chung, G.T.; Lun, S.W.; To, K.-F.; Choy, K.-W.; Lau, K.-M.; Siu, S.P.-K.; Guan, X.-Y.; Ngan, R.K.-C.; Yip, T.T.-C.; et al. MiR-31 Is Consistently Inactivated in EBV-Associated Nasopharyngeal Carcinoma and Contributes to Its Tumorigenesis. *Mol. Cancer* **2014**, *13*, 184–184.

119. Lyu, X.; Fang, W.; Cai, L.; Zheng, H.; Ye, Y.; Zhang, L.; Li, J.; Peng, H.; Cho, W.C.S.; Wang, E.; et al. TGFβR2 Is a Major Target of MiR-93 in Nasopharyngeal Carcinoma Aggressiveness. *Mol. Cancer* **2014**, *13*, 51, doi:10.1186/1476-4598-13-51.

120. Xu, Y.-F.; Mao, Y.-P.; Li, Y.-Q.; Ren, X.-Y.; He, Q.-M.; Tang, X.-R.; Sun, Y.; Liu, N.; Ma, J. MicroRNA-93 Promotes Cell Growth and Invasion in Nasopharyngeal Carcinoma by Targeting Disabled Homolog-2. *Cancer Lett.* **2015**, *363*, 146–155, doi:10.1016/j.canlet.2015.04.006.

121. Qi, X.; Li, J.; Zhou, C.; Lv, C.; Tian, M. MiR-142-3p Suppresses SOCS6 Expression and Promotes Cell Proliferation in Nasopharyngeal Carcinoma. *Cell. Physiol. Biochem.* **2015**, *36*, 1743–1752, doi:10.1159/000430147.

122. Cameron, J.E.; Yin, Q.; Fewell, C.; Lacey, M.; McBride, J.; Wang, X.; Lin, Z.; Schaefer, B.C.; Flemington, E.K. Epstein-Barr Virus Latent Membrane Protein 1 Induces Cellular MicroRNA MiR-146a, a Modulator of Lymphocyte Signaling Pathways. *J. Virol.* **2008**, *82*, 1946–1958, doi:10.1128/JVI.02136-07.

123. Hung, P.-S.; Liu, C.-J.; Chou, C.-S.; Kao, S.-Y.; Yang, C.-C.; Chang, K.-W.; Chiu, T.-H.; Lin, S.-C. MiR-146a Enhances the Oncogenicity of Oral Carcinoma by Concomitant Targeting of the IRAK1, TRAF6 and NUMB Genes. *PloS One* **2013**, *8*, e79926, doi:10.1371/journal.pone.0079926.

124. Rosato, P.; Anastasiadou, E.; Garg, N.; Lenze, D.; Boccellato, F.; Vincenti, S.; Severa, M.; Coccia, E.M.; Bigi, R.; Cirone, M.; et al. Differential Regulation of MiR-21 and MiR-146a by Epstein – Barr. *Leukemia* **2012**, *26*, 2343–2352, doi:10.1038/leu.2012.108.

125. Luo, Z.; Zhang, L.; Li, Z.; Jiang, C.; Dai, Y.; Liu, X.; Zheng, Y.; Yu, H.; Xiang, J.; Li, G. MiR-149 Promotes Epithelial-Mesenchymal Transition and Invasion in Nasopharyngeal Carcinoma Cells. *Zhong Nan Da Xue Xue Bao Yi Xue Ban* **2011**, *36*, 604–609, doi:10.3969/j.issn.1672-7347.2011.07.004.

126. Ding, J.; Huang, S.; Wu, S.; Zhao, Y.; Liang, L.; Yan, M.; Ge, C.; Yao, J.; Chen, T.; Wan, D.; et al. Gain of MiR-151 on Chromosome 8q24.3 Facilitates Tumour Cell Migration and Spreading through Downregulating RhoGDIA. *Nat. Cell Biol.* **2010**, *12*, 390–399, doi:10.1038/ncb2039.

127. Liu, Y.; Cai, H.; Liu, J.; Fan, H.; Wang, Z.; Wang, Q.; Shao, M.; Sun, X.; Diao, J.; Liu, Y.; et al. A MiR-151 Binding Site Polymorphism in the 3′-Untranslated Region of the Cyclin E1 Gene Associated with Nasopharyngeal Carcinoma. *Biochem. Biophys. Res. Commun.* **2013**, *432*, 660–665, doi:10.1016/j.bbrc.2013.02.024.

128. Chen, H.-C.; Chen, G.-H.; Chen, Y.-H.; Liao, W.-L.; Liu, C.-Y.; Chang, K.-P.; Chang, Y.-S.; Chen, S.-J. MicroRNA Deregulation and Pathway Alterations in Nasopharyngeal Carcinoma. *Br. J. Cancer* **2009**, *100*, 1002–1011, doi:10.1038/sj.bjc.6604948.

129. Czochor, J.R.; Sulkowski, P.; Glazer, P.M. MiR-155 Over-Expression Promotes Genomic Instability by Reducing High-Fidelity Polymerase Delta Expression and Activating Error-Prone DSB Repair. *Mol. Cancer Res. MCR* **2016**, doi:10.1158/1541-7786.MCR-15-0399.

130. Kim, S.; Song, J.H.; Kim, S.; Qu, P.; Martin, B.K.; Sehareen, W.S.; Haines, D.C.; Lin, P.C.; Sharan, S.K.; Chang, S. Loss of Oncogenic MiR-155 in Tumor Cells Promotes Tumor Growth by Enhancing C/EBP-β-Mediated MDSC Infiltration. *Oncotarget* **2016**, doi:10.18632/oncotarget.7150.

131. Li, D.; Jian, W.; Wei, C.; Song, H.; Gu, Y.; Luo, Y.; Fang, L. Down-Regulation of MiR-181b Promotes Apoptosis by Targeting CYLD in Thyroid Papillary Cancer. *Int. J. Clin. Exp. Pathol.* **2014**, *7*, 7672–7680.

132. Xu, R.-X.; Liu, R.-Y.; Wu, C.-M.; Zhao, Y.-S.; Li, Y.; Yao, Y.-Q.; Xu, Y.-H. DNA Damage-Induced NF-KB Activation in Human Glioblastoma Cells Promotes MiR-181b Expression and Cell Proliferation. *Cell. Physiol. Biochem. Int. J. Exp. Cell. Physiol. Biochem. Pharmacol.* **2015**, *35*, 913–925, doi:10.1159/000369748.

133. Zheng, Y.; Lv, X.; Wang, X.; Wang, B.; Shao, X.; Huang, Y.; Shi, L.; Chen, Z.; Huang, J.; Huang, P. MiR-181b Promotes Chemoresistance in Breast Cancer by Regulating Bim Expression. *Oncol. Rep.* **2016**, *35*, 683–690, doi:10.3892/or.2015.4417.

134. Li, J.; Fu, H.; Xu, C.; Tie, Y.; Xing, R.; Zhu, J.; Qin, Y.; Sun, Z.; Zheng, X. MiR-183 Inhibits TGF-Beta1-Induced Apoptosis by Downregulation of PDCD4 Expression in Human Hepatocellular Carcinoma Cells. *BMC Cancer* **2010**, *10*, 354, doi:10.1186/1471-2407-10-354.

135. Sarver, A.L.; Li, L.; Subramanian, S. MicroRNA MiR-183 Functions as an Oncogene by Targeting the Transcription Factor EGR1 and Promoting Tumor Cell Migration. *Cancer Res.* **2010**, *70*, 9570–9580, doi:10.1158/0008-5472.CAN-10-2074.

136. Li, G.; Wang, Y.; Liu, Y.; Su, Z.; Liu, C.; Ren, S.; Deng, T.; Huang, D.; Tian, Y.; Qiu, Y. MiR-185-3p Regulates Nasopharyngeal Carcinoma Radioresistance by Targeting WNT2B in Vitro. *Cancer Sci.* **2014**, *105*, 1560–1568, doi:10.1111/cas.12555.

137. Xu, J.; Ai, Q.; Cao, H.; Liu, Q. MiR-185-3p and MiR-324-3p Predict Radiosensitivity of Nasopharyngeal Carcinoma and Modulate Cancer Cell Growth and Apoptosis by Targeting SMAD7. *Med. Sci. Monit. Int. Med. J. Exp. Clin. Res.* **2015**, *21*, 2828–2836, doi:10.12659/MSM.895660.

138. Nagpal, N.; Ahmad, H.M.; Chameettachal, S.; Sundar, D.; Ghosh, S.; Kulshreshtha, R. HIF-Inducible MiR-191 Promotes Migration in Breast Cancer through Complex Regulation of TGFβ-Signaling in Hypoxic Microenvironment. *Sci. Rep.* **2015**, *5*, 9650, doi:10.1038/srep09650.

139. Li, S.; Li, F.; Niu, R.; Zhang, H.; Cui, A.; An, W.; Wang, X. Mir-192 Suppresses Apoptosis and Promotes Proliferation in Esophageal Aquamous Cell Caicinoma by Targeting Bim. *Int. J. Clin. Exp. Pathol.* **2015**, *8*, 8048–8056.

140. Yan-Chun, L.; Hong-Mei, Y.; Zhi-Hong, C.; Qing, H.; Yan-Hong, Z.; Ji-Fang, W. MicroRNA-192-5p Promote the Proliferation and Metastasis of Hepatocellular Carcinoma Cell by Targeting SEMA3A. *Appl. Immunohistochem. Mol. Morphol. AIMM Off. Publ. Soc. Appl. Immunohistochem.* **2015**, doi:10.1097/PAI.0000000000000296.

141. Yeh, D.-W.; Chen, Y.-S.; Lai, C.-Y.; Liu, Y.-L.; Lu, C.-H.; Lo, J.-F.; Chen, L.; Hsu, L.-C.; Luo, Y.; Xiang, R.; et al. Downregulation of COMMD1 by MiR-205 Promotes a Positive Feedback Loop for Amplifying Inflammatory- and Stemness-Associated Properties of Cancer Cells. *Cell Death Differ.* **2015**, doi:10.1038/cdd.2015.147.

142. Tang, X.; Chen, L.; Yan, X.; Li, Y.; Xiong, Y.; Zhou, X. Overexpression of MiR-210 Is Associated with Poor Prognosis of Acute Myeloid Leukemia. *Med. Sci. Monit. Int. Med. J. Exp. Clin. Res.* **2015**, *21*, 3427–3433.

143. Zhang, C.; Tian, W.; Meng, L.; Qu, L.; Shou, C. PRL-3 Promotes Gastric Cancer Migration and Invasion through a NF-KB-HIF-1α-MiR-210 Axis. *J. Mol. Med. Berl. Ger.* **2015**, doi:10.1007/s00109-015-1350-7.

144. Deng, M.; Ye, Q.; Qin, Z.; Zheng, Y.; He, W.; Tang, H.; Zhou, Y.; Xiong, W.; Zhou, M.; Li, X.; et al. MiR-214 Promotes Tumorigenesis by Targeting Lactotransferrin in Nasopharyngeal Carcinoma. *Tumour Biol. J. Int. Soc. Oncodevelopmental Biol. Med.* **2013**, *34*, 1793–1800, doi:10.1007/s13277-013-0718-y.

145. Phatak, P.; Byrnes, K.A.; Mansour, D.; Liu, L.; Cao, S.; Li, R.; Rao, J.N.; Turner, D.J.; Wang, J.-Y.; Donahue, J.M. Overexpression of MiR-214-3p in Esophageal Squamous Cancer Cells Enhances Sensitivity to Cisplatin by Targeting Survivin Directly and Indirectly through CUG-BP1. *Oncogene* **2015**, doi:10.1038/onc.2015.271.

146. Wang, F.; Lv, P.; Liu, X.; Zhu, M.; Qiu, X. MicroRNA-214 Enhances the Invasion Ability of Breast Cancer Cells by Targeting P53. *Int. J. Mol. Med.* **2015**, *35*, 1395–1402, doi:10.3892/ijmm.2015.2123.

147. Zhang, Z.-C.; Li, Y.-Y.; Wang, H.-Y.; Fu, S.; Wang, X.-P.; Zeng, M.-S.; Zeng, Y.-X.; Shao, J.-Y. Knockdown of MiR-214 Promotes Apoptosis and Inhibits Cell Proliferation in Nasopharyngeal Carcinoma. *PloS One* **2014**, *9*, e86149, doi:10.1371/journal.pone.0086149.

148. Liu, W.; Song, N.; Yao, H.; Zhao, L.; Liu, H.; Li, G. MiR-221 and MiR-222 Simultaneously Target RECK and Regulate Growth and Invasion of Gastric Cancer Cells. *Med. Sci. Monit. Int. Med. J. Exp. Clin. Res.* **2015**, *21*, 2718–2725, doi:10.12659/MSM.894324.

149. le Sage, C.; Nagel, R.; Egan, D.A.; Schrier, M.; Mesman, E.; Mangiola, A.; Anile, C.; Maira, G.; Mercatelli, N.; Ciafrè, S.A.; et al. Regulation of the P27(Kip1) Tumor Suppressor by MiR-221 and

MiR-222 Promotes Cancer Cell Proliferation. *EMBO J.* **2007**, *26*, 3699–3708, doi:10.1038/sj.emboj.7601790.

150. Zhong, C.; Ding, S.; Xu, Y.; Huang, H. MicroRNA-222 Promotes Human Non-Small Cell Lung Cancer H460 Growth by Targeting P27. *Int. J. Clin. Exp. Med.* **2015**, *8*, 5534–5540.

151. Cui, R.; Kim, T.; Fassan, M.; Meng, W.; Sun, H.-L.; Jeon, Y.-J.; Vicentini, C.; Tili, E.; Peng, Y.; Scarpa, A.; et al. MicroRNA-224 Is Implicated in Lung Cancer Pathogenesis through Targeting Caspase-3 and Caspase-7. *Oncotarget* **2015**, *6*, 21802–21815, doi:10.18632/oncotarget.5224.

152. Cui, R.; Meng, W.; Sun, H.-L.; Kim, T.; Ye, Z.; Fassan, M.; Jeon, Y.-J.; Li, B.; Vicentini, C.; Peng, Y.; et al. MicroRNA-224 Promotes Tumor Progression in Nonsmall Cell Lung Cancer. *Proc. Natl. Acad. Sci. U. S. A.* **2015**, *112*, E4288-4297, doi:10.1073/pnas.1502068112.

153. Ding, W.; Fan, X.-L.; Xu, X.; Huang, J.-Z.; Xu, S.-H.; Geng, Q.; Li, R.; Chen, D.; Yan, G.-R. Epigenetic Silencing of ITGA2 by MiR-373 Promotes Cell Migration in Breast Cancer. *PloS One* **2015**, *10*, e0135128, doi:10.1371/journal.pone.0135128.

154. Cai, J.; Guan, H.; Fang, L.; Yang, Y.; Zhu, X.; Yuan, J.; Wu, J.; Li, M. MicroRNA-374a Activates Wnt/β-Catenin Signaling to Promote Breast Cancer Metastasis. *J. Clin. Invest.* **2013**, *123*, 566–579, doi:10.1172/JCI65871.

155. He, W.; Feng, L.; Xia, D.; Han, N. MiR-374a Promotes the Proliferation of Human Osteosarcoma by Downregulating FOXO1 Expression. *Int. J. Clin. Exp. Med.* **2015**, *8*, 3482–3489.

156. Wang, Y.; Xin, H.; Han, Z.; Sun, H.; Gao, N.; Yu, H. MicroRNA-374a Promotes Esophageal Cancer Cell Proliferation via Axin2 Suppression. *Oncol. Rep.* **2015**, *34*, 1988–1994, doi:10.3892/or.2015.4182.

157. Xu, X.; Wang, W.; Su, N.; Zhu, X.; Yao, J.; Gao, W.; Hu, Z.; Sun, Y. MiR-374a Promotes Cell Proliferation, Migration and Invasion by Targeting SRCIN1 in Gastric Cancer. *FEBS Lett.* **2015**, *589*, 407–413, doi:10.1016/j.febslet.2014.12.027.

158. Chen, L.; Tang, Y.; Wang, J.; Yan, Z.; Xu, R. MiR-421 Induces Cell Proliferation and Apoptosis Resistance in Human Nasopharyngeal Carcinoma via Downregulation of FOXO4. *Biochem. Biophys. Res. Commun.* **2013**, *435*, 745–750, doi:10.1016/j.bbrc.2013.05.056.

159. Yi, C.; Wang, Q.; Wang, L.; Huang, Y.; Li, L.; Liu, L.; Zhou, X.; Xie, G.; Kang, T.; Wang, H.; et al. MiR-663, a MicroRNA Targeting P21(WAF1/CIP1), Promotes the Proliferation and Tumorigenesis of Nasopharyngeal Carcinoma. *Oncogene* **2012**, *31*, 4421–4433, doi:10.1038/onc.2011.629.

160. Fang, Y.; Zhu, X.; Wang, J.; Li, N.; Li, D.; Sakib, N.; Sha, Z.; Song, W. MiR-744 Functions as a Proto-Oncogene in Nasopharyngeal Carcinoma Progression and Metastasis via Transcriptional Control of ARHGAP5. *Oncotarget* **2015**, *6*, 13164–13175, doi:10.18632/oncotarget.3754.

161. Yu, Q.; Zhang, F.; Du, Z.; Xiang, Y. Up-Regulation of Serum MiR-744 Predicts Poor Prognosis in Patients with Nasopharyngeal Carcinoma. *Int. J. Clin. Exp. Med.* **2015**, *8*, 13296–13302.

162. Zhou, W.; Li, Y.; Gou, S.; Xiong, J.; Wu, H.; Wang, C.; Yan, H.; Liu, T. MiR-744 Increases Tumorigenicity of Pancreatic Cancer by Activating Wnt/β-Catenin Pathway. *Oncotarget* **2015**, *6*, 37557–37569, doi:10.18632/oncotarget.5317.

163. Li, Y.; Chen, X. MiR-4792 Inhibits Epithelial-Mesenchymal Transition and Invasion in Nasopharyngeal Carcinoma by Targeting FOXC1. *Biochem. Biophys. Res. Commun.* **2015**, *468*, 863–869, doi:10.1016/j.bbrc.2015.11.045.

164. Cai, J.; Yang, C.; Yang, Q.; Ding, H.; Jia, J.; Guo, J.; Wang, J.; Wang, Z. Deregulation of Let-7e in Epithelial Ovarian Cancer Promotes the Development of Resistance to Cisplatin. *Oncogenesis* **2013**, *2*, e75, doi:10.1038/oncsis.2013.39.

165. Mitra, D.; Das, P.M.; Huynh, F.C.; Jones, F.E. Jumonji/ARID1 B (JARID1B) Protein Promotes Breast Tumor Cell Cycle Progression through Epigenetic Repression of MicroRNA Let-7e. *J. Biol. Chem.* **2011**, *286*, 40531–40535, doi:10.1074/jbc.M111.304865.

166. Jia, H.; Zhang, Z.; Zou, D.; Wang, B.; Yan, Y.; Luo, M.; Dong, L.; Yin, H.; Gong, B.; Li, Z.; et al. MicroRNA-10a Is down-Regulated by DNA Methylation and Functions as a Tumor Suppressor in Gastric Cancer Cells. *PloS One* **2014**, *9*, e88057, doi:10.1371/journal.pone.0088057.

167. Zeng, T.; Li, G. MicroRNA-10a Enhances the Metastatic Potential of Cervical Cancer Cells by Targeting Phosphatase and Tensin Homologue. *Mol. Med. Rep.* **2014**, *10*, 1377–1382, doi:10.3892/mmr.2014.2370.

168. Liu, B.; Wu, X.; Liu, B.; Wang, C.; Liu, Y.; Zhou, Q.; Xu, K. MiR-26a Enhances Metastasis Potential of Lung Cancer Cells via AKT Pathway by Targeting PTEN. *Biochim. Biophys. Acta* **2012**, *1822*, 1692–1704, doi:10.1016/j.bbadis.2012.07.019.

169. Lu, J.; He, M.-L.; Wang, L.; Chen, Y.; Liu, X.; Dong, Q.; Chen, Y.-C.; Peng, Y.; Yao, K.-T.; Kung, H.-F.; et al. MiR-26a Inhibits Cell Growth and Tumorigenesis of Nasopharyngeal Carcinoma through Repression of EZH2. *Cancer Res.* **2011**, *71*, 225–233, doi:10.1158/0008-5472.CAN-10-1850.

170. Yu, L.; Lu, J.; Zhang, B.; Liu, X.; Wang, L.; Li, S.-Y.; Peng, X.-H.; Xu, X.; Tian, W.-D.; Li, X.-P. MiR-26a Inhibits Invasion and Metastasis of Nasopharyngeal Cancer by Targeting EZH2. *Oncol. Lett.* **2013**, *5*, 1223–1228, doi:10.3892/ol.2013.1173.

171. Lei, C.-J.; Li, L.; Gao, X.; Zhang, J.; Pan, Q.-Y.; Long, H.-C.; Chen, C.-Z.; Ren, D.-F.; Zheng, G. Hsa-MiR-132 Inhibits Proliferation of Hepatic Carcinoma Cells by Targeting YAP. *Cell Biochem. Funct.* **2015**, *33*, 326–333, doi:10.1002/cbf.3119.

172. Li, W.; Zhang, J.; Chen, T.; Yin, P.; Yang, J.; Cao, Y. MiR-132 Upregulation Promotes Gastric Cancer Cell Growth through Suppression of FoxO1 Translation. *Tumour Biol. J. Int. Soc. Oncodevelopmental Biol. Med.* **2015**, doi:10.1007/s13277-015-3924-y.

173. Li, Y.; Zu, L.; Wang, Y.; Wang, M.; Chen, P.; Zhou, Q. MiR-132 Inhibits Lung Cancer Cell Migration and Invasion by Targeting SOX4. *J. Thorac. Dis.* **2015**, *7*, 1563–1569, doi:10.3978/j.issn.2072-1439.2015.09.06.

174. Liu, F.; Wang, J.; Fu, Q.; Zhang, X.; Wang, Y.; Liu, J.; Huang, J.; Lv, X. VEGF-Activated MiR-144 Regulates Autophagic Survival of Prostate Cancer Cells against Cisplatin. *Tumour Biol. J. Int. Soc. Oncodevelopmental Biol. Med.* **2015**, doi:10.1007/s13277-015-4383-1.

175. Sun, L.; Bian, G.; Meng, Z.; Dang, G.; Shi, D.; Mi, S. MiR-144 Inhibits Uveal Melanoma Cell Proliferation and Invasion by Regulating c-Met Expression. *PloS One* **2015**, *10*, e0124428, doi:10.1371/journal.pone.0124428.

176. Zhang, L.-Y.; Ho-Fun Lee, V.; Wong, A.M.G.; Kwong, D.L.-W.; Zhu, Y.-H.; Dong, S.-S.; Kong, K.-L.; Chen, J.; Tsao, S.-W.; Guan, X.-Y.; et al. MicroRNA-144 Promotes Cell Proliferation, Migration and Invasion in Nasopharyngeal Carcinoma through Repression of PTEN. *Carcinogenesis* **2013**, *34*, 454–463, doi:10.1093/carcin/bgs346.

177. Li, Y.; Kuscu, C.; Banach, A.; Zhang, Q.; Pulkoski-Gross, A.; Kim, D.; Liu, J.; Roth, E.; Li, E.; Shroyer, K.R.; et al. MiR-181a-5p Inhibits Cancer Cell Migration and Angiogenesis via Downregulation of Matrix Metalloproteinase-14. *Cancer Res.* **2015**, *75*, 2674–2685, doi:10.1158/0008-5472.CAN-14-2875.

178. Ma, Z.; Qiu, X.; Wang, D.; Li, Y.; Zhang, B.; Yuan, T.; Wei, J.; Zhao, B.; Zhao, X.; Lou, J.; et al. MiR-181a-5p Inhibits Cell Proliferation and Migration by Targeting Kras in Non-Small Cell Lung Cancer A549 Cells. *Acta Biochim. Biophys. Sin.* **2015**, *47*, 630–638, doi:10.1093/abbs/gmv054.

179. Sun, X.; Charbonneau, C.; Wei, L.; Chen, Q.; Terek, R.M. MiR-181a Targets RGS16 to Promote Chondrosarcoma Growth, Angiogenesis, and Metastasis. *Mol. Cancer Res. MCR* **2015**, *13*, 1347–1357, doi:10.1158/1541-7786.MCR-14-0697.

180. Zhao, J.; Nie, Y.; Wang, H.; Lin, Y. MiR-181a Suppresses Autophagy and Sensitizes Gastric Cancer Cells to Cisplatin. *Gene* **2016**, *576*, 828–833, doi:10.1016/j.gene.2015.11.013.

181. Qin, C.-Z.; Lou, X.-Y.; Lv, Q.-L.; Cheng, L.; Wu, N.-Y.; Hu, L.; Zhou, H.-H. MicroRNA-184 Acts as a Potential Diagnostic and Prognostic Marker in Epithelial Ovarian Cancer and Regulates Cell Proliferation, Apoptosis and Inflammation. *Pharm.* **2015**, *70*, 668–673.

182. Su, Z.; Chen, D.; Li, Y.; Zhang, E.; Yu, Z.; Chen, T.; Jiang, Z.; Ni, L.; Yang, S.; Gui, Y.; et al. MicroRNA-184 Functions as Tumor Suppressor in Renal Cell Carcinoma. *Exp. Ther. Med.* **2015**, *9*, 961–966, doi:10.3892/etm.2015.2199.

183. Yuan, Q.; Gao, W.; Liu, B.; Ye, W. Upregulation of MiR-184 Enhances the Malignant Biological Behavior of Human Glioma Cell Line A172 by Targeting FIH-1. *Cell. Physiol. Biochem. Int. J. Exp. Cell. Physiol. Biochem. Pharmacol.* **2014**, *34*, 1125–1136, doi:10.1159/000366326.

184. Zhen, Y.; Liu, Z.; Yang, H.; Yu, X.; Wu, Q.; Hua, S.; Long, X.; Jiang, Q.; Song, Y.; Cheng, C.; et al. Tumor Suppressor PDCD4 Modulates MiR-184-Mediated Direct Suppression of C-MYC and BCL2 Blocking Cell Growth and Survival in Nasopharyngeal Carcinoma. *Cell Death Dis.* **2013**, *4*, e872, doi:10.1038/cddis.2013.376.

185. Qu, J.-Q.; Yi, H.-M.; Ye, X.; Zhu, J.-F.; Yi, H.; Li, L.-N.; Xiao, T.; Yuan, L.; Li, J.-Y.; Wang, Y.-Y.; et al. MiRNA-203 Reduces Nasopharyngeal Carcinoma Radioresistance by Targeting IL8/AKT Signaling. *Mol. Cancer Ther.* **2015**, *14*, 2653–2664, doi:10.1158/1535-7163.MCT-15-0461.

186. Yu, H.; Lu, J.; Zuo, L.; Yan, Q.; Yu, Z.; Li, X.; Huang, J.; Zhao, L.; Tang, H.; Luo, Z.; et al. Epstein-Barr Virus Downregulates MicroRNA 203 through the Oncoprotein Latent Membrane Protein 1: A Contribution to Increased Tumor Incidence in Epithelial Cells. *J. Virol.* **2012**, *86*, 3088–3099, doi:10.1128/JVI.05901-11.

187. Huang, X.; Huang, M.; Kong, L.; Li, Y. MiR-372 Suppresses Tumour Proliferation and Invasion by Targeting IGF2BP1 in Renal Cell Carcinoma. *Cell Prolif.* **2015**, *48*, 593–599, doi:10.1111/cpr.12207.

188. Tan, J.-K.; Tan, E.-L.; Gan, S.-Y. Elucidating the Roles of MiR-372 in Cell Proliferation and Apoptosis of Nasopharyngeal Carcinoma TW01 Cells. *Exp. Oncol.* **2014**, *36*, 170–173.

189. Tu, H.-F.; Chang, K.-W.; Cheng, H.-W.; Liu, C.-J. Upregulation of MiR-372 and -373 Associates with Lymph Node Metastasis and Poor Prognosis of Oral Carcinomas. *The Laryngoscope* **2015**, *125*, E365-370, doi:10.1002/lary.25464.

190. Li, H.; Dai, S.; Zhen, T.; Shi, H.; Zhang, F.; Yang, Y.; Kang, L.; Liang, Y.; Han, A. Clinical and Biological Significance of MiR-378a-3p and MiR-378a-5p in Colorectal Cancer. *Eur. J. Cancer Oxf. Engl. 1990* **2014**, *50*, 1207–1221, doi:10.1016/j.ejca.2013.12.010.

191. Skrzypek, K.; Tertil, M.; Golda, S.; Ciesla, M.; Weglarczyk, K.; Collet, G.; Guichard, A.; Kozakowska, M.; Boczkowski, J.; Was, H.; et al. Interplay between Heme Oxygenase-1 and MiR-378 Affects Non-Small Cell Lung Carcinoma Growth, Vascularization, and Metastasis. *Antioxid. Redox Signal.* **2013**, *19*, 644–660, doi:10.1089/ars.2013.5184.

192. Yu, B.-L.; Peng, X.-H.; Zhao, F.-P.; Liu, X.; Lu, J.; Wang, L.; Li, G.; Chen, H.-H.; Li, X.-P. MicroRNA-378 Functions as an Onco-MiR in Nasopharyngeal Carcinoma by Repressing TOB2 Expression. *Int. J. Oncol.* **2014**, *44*, 1215–1222, doi:10.3892/ijo.2014.2283.

193. Popov, A.; Szabo, A.; Mandys, V. Small Nucleolar RNA U91 Is a New Internal Control for Accurate MicroRNAs Quantification in Pancreatic Cancer. *BMC Cancer* **2015**, *15*, 774, doi:10.1186/s12885-015-1785-9.

194. Peltier, H.J.; Latham, G.J. Normalization of MicroRNA Expression Levels in Quantitative RT-PCR Assays: Identification of Suitable Reference RNA Targets in Normal and Cancerous Human Solid Tissues. *RNA N. Y. N* **2008**, *14*, 844–852, doi:10.1261/rna.939908.

195. Tokar, T.; Pastrello, C.; Rossos, A.E.M.; Abovsky, M.; Hauschild, A.-C.; Tsay, M.; Lu, R.; Jurisica, I. MirDIP 4.1—Integrative Database of Human MicroRNA Target Predictions. *Nucleic Acids Res.* **2018**, *46*, D360–D370, doi:10.1093/nar/gkx1144.

196. Shirdel, E.A.; Xie, W.; Mak, T.W.; Jurisica, I. NAViGaTing the Micronome – Using Multiple MicroRNA Prediction Databases to Identify Signalling Pathway-Associated MicroRNAs. *PLOS ONE* **2011**, *6*, e17429, doi:10.1371/journal.pone.0017429.

197. Croft, D.; Mundo, A.F.; Haw, R.; Milacic, M.; Weiser, J.; Wu, G.; Caudy, M.; Garapati, P.; Gillespie, M.; Kamdar, M.R.; et al. The Reactome Pathway Knowledgebase. *Nucleic Acids Res.* **2014**, *42*, D472–D477, doi:10.1093/nar/gkt1102.

198. Shannon, P.; Markiel, A.; Ozier, O.; Baliga, N.S.; Wang, J.T.; Ramage, D.; Amin, N.; Schwikowski, B.; Ideker, T. Cytoscape: A Software Environment for Integrated Models of Biomolecular Interaction Networks. *Genome Res.* **2003**, *13*, 2498–2504, doi:10.1101/gr.1239303.

199. Fabregat, A.; Jupe, S.; Matthews, L.; Sidiropoulos, K.; Gillespie, M.; Garapati, P.; Haw, R.; Jassal, B.; Korninger, F.; May, B.; et al. The Reactome Pathway Knowledgebase. *Nucleic Acids Res.* **2018**, *46*, D649–D655, doi:10.1093/nar/gkx1132.
